# Liver biopsy derived induced pluripotent stem cells provide unlimited supply for the generation of hepatocyte-like cells

**DOI:** 10.1371/journal.pone.0221762

**Published:** 2019-08-29

**Authors:** Diego Calabrese, Guglielmo Roma, Sebastian Bergling, Walter Carbone, Valentina Mele, Sandro Nuciforo, Isabel Fofana, Benedetta Campana, Dagmara Szkolnicka, David C. Hay, Jan Tchorz, Tewis Bouwmeester, Stefan Wieland, Markus H. Heim

**Affiliations:** 1 Department of Biomedicine, University Hospital Basel, University of Basel, Basel, Switzerland; 2 Novartis Institutes for BioMedical Research, Novartis Pharma AG, Forum 1 Novartis Campus, Basel, Switzerland; 3 Division of Gastroenterology and Hepatology, University Hospital Basel, Basel, Switzerland; 4 MRC Centre for Regenerative Medicine, 5 Little France Drive, Edinburgh, United Kingdom; University of Pécs Medical School, HUNGARY

## Abstract

**Background & aims:**

Hepatocyte-like cells (HLCs) differentiated from induced pluripotent stem cells (iPSCs) have emerged as a promising cell culture model to study metabolism, biotransformation, viral infections and inherited liver diseases. iPSCs provide an unlimited supply for the generation of HLCs, but incomplete HLC differentiation remains a major challenge. iPSC may carry-on a tissue of origin dependent expression memory influencing iPSC differentiation into different cell types. Whether liver derived iPSCs (Li-iPSCs) would allow the generation of more fully differentiated HLCs is not known.

**Methods:**

In the current study, we used primary liver cells (PLCs) expanded from liver needle biopsies and reprogrammed them into Li-iPSCs using a non-integrative Sendai virus-based system. Li-iPSCs were differentiated into HLCs using established differentiation protocols. The HLC phenotype was characterized at the protein, functional and transcriptional level. RNA sequencing data were generated from the originating liver biopsies, the Li-iPSCs, fibroblast derived iPSCs, and differentiated HLCs, and used to characterize and compare their transcriptome profiles.

**Results:**

Li-iPSCs indeed retain a liver specific transcriptional footprint. Li-iPSCs can be propagated to provide an unlimited supply of cells for differentiation into Li-HLCs. Similar to HLCs derived from fibroblasts, Li-HLCs could not be fully differentiated into hepatocytes. Relative to the originating liver, Li-HLCs showed lower expression of liver specific transcription factors and increased expression of genes involved in the differentiation of other tissues.

**Conclusions:**

PLCs and Li-iPSCs obtained from small pieces of human needle liver biopsies constitute a novel unlimited source for the production of HLCs. Despite the preservation of a liver specific gene expression footprint in Li-iPSCs, the generation of fully differentiated hepatocytes cannot be achieved with the current differentiation protocols.

## Introduction

Current *in vitro* models for the study of liver functions and pathology predominantly rely on hepatoma cell lines and primary human hepatocyte (PHH) cultures. Although hepatoma cell lines are characterized by a stable phenotype, they show limited expression of liver enzymes [1]. Furthermore, since these cells are clonal in origin, they poorly represent the intra- and inter-patient cell heterogeneity. PHHs are considered the gold standard for toxicology studies because they maintain numerous hepatocyte functions. However, their hepatic phenotype is unstable over long term culturing, and their availability is limited (reviewed in [2]).

More recently, stem cell-derived hepatocyte-like cells (HLCs) have emerged as an alternative *in vitro* hepatocyte culture system [3]. HLCs derived from human induced pluripotent stem cells (iPSCs) have the potential to overcome the limitations of current models, as they can be produced in unlimited numbers, and they more closely resemble authentic hepatocytes [4]. Indeed, HLCs have been shown to display liver functions, such as inducible Cytochromes P450 (CYPs), liver specific protein expression, bile and lipid production, and glycogen storage capacity [5]. In addition, iPSC-derived HLCs maintain the patient genetic background, allowing for the study of liver diseases in a personalized setting, where the pathology by itself or the outcome to treatment are linked to a specific genotype ([6] and reviewed in [7]). However, derivation of iPSCs and the usage of HLCs for personalized medicine is restricted by ethical and procedural constrains [8]. Therefore, derivation of patient-specific iPSC lines would ideally start from surplus material of samples collected for diagnostic purposes, such as human liver needle biopsies. Primary liver cell derivation from needle biopsies has not yet been described, even though protocols starting from surgically resected liver tissue have been reported [9, 10]. Also, reprogramming of liver-derived cells into iPSC is not very well defined, and so far has only been done starting from PHH [11, 12]. Furthermore, whether liver-derived iPSCs retain residual liver specific transcriptional profiles that facilitate their differentiation into HLCs is currently not known. Although residual transcriptional memory in iPSCs of the tissue of origin has been described (reviewed in [13]), its influence on the differentiation of these cells towards different cell lineages is still controversial [13].

In this study we aimed to establish patient-specific HLCs starting from human liver needle biopsies. We developed a protocol for generating a large number of human PLCs from small pieces of human liver needle biopsy tissue, and successfully reprogrammed them into pluripotent liver (Li-)iPSCs. Using previously established protocols [3], we then differentiated the Li-iPSCs into hepatocyte-like cells (Li-HLCs). The workflow allowed us to compare gene expression profiles of Li-iPSCs and Li-HLCs to the originating liver biopsies. Li-iPSCs indeed conserved a subtle liver gene expression profile. However, compared to fibroblast-derived iPSCs, Li-iPSCs did not give rise to HLCs with improved liver-specific gene expression. The incomplete differentiation of iPSCs to HLCs was associated with a suboptimal expression of liver-specific transcription factors, and with the expression of genes involved in the development of organs other than the liver. These observations provide starting points for strategies aimed at improving differentiation of iPSCs towards hepatocytes.

## Methods

### Liver biopsies

Eight human liver needle biopsies were obtained at the University Hospital Basel, Switzerland from eight patients undergoing diagnostic liver biopsy (S1 Table). The study was carried out in accordance with The Code of Ethics of the World Medical Association (Declaration of Helsinki) and was approved by the Ethics Committee of North Western Switzerland (Authorization number EKNZ 2014–362). Written informed consent was obtained from all patients enrolled in this study (S1 Table). Three to five millimeters of liver biopsy cylinders of 1mm diameter were transferred immediately after collection (*ex vivo*) into complete culturing medium (S1 File) and transported at room temperature to the laboratory for further processing. Additionally, five to ten millimeters of the liver biopsy cylinder were snap frozen by immersion in liquid nitrogen and stored in liquid nitrogen vapors until RNA extraction.

Patients enrolled in this study were affected by mild liver diseases. Histological and molecular analysis showed none or mild steatosis or steatohepatitis with different etiology, limited immune infiltration and no hepatotropic virus infections.

### Derivation of human primary liver cells (PLCs)

Immediately after collection, the surplus liver biopsy was washed three times in 1X Dulbecco’s PBS (DPBS) and cut in pieces of 0.3–0.5 mm. The tissue samples were then partially digested by incubation with trypsin-EDTA (0.05%, ref. 25300054 Thermo Fisher Scientific) at 37°C for 15 minutes and vortexing every 5 minutes. The trypsin-EDTA solution was carefully removed by aspiration, and the liver biopsy pieces were washed twice with complete medium (S1 File). The samples were then transferred to 6-well culture dishes coated with reduced growth factor basement membrane matrix (ref. A1413301, Geltrex LDEV-Free, Thermo Fisher Scientific) and covered by complete medium (S1 File). Twenty-four hours later, complete medium was replaced by basal medium (S1 File). Tissue samples were left to adhere to the culture dishes for 48 hours before changing the medium for the first time, and non-adherent biopsy pieces were discarded. PLC outgrowth from the adherent biopsy pieces was observed after 7 to 10 days in culture. After 15–20 days of culturing, when the PLC monolayer encompassed about 300–500 cells, the biopsy pieces were removed and discarded. The adherent PLCs (passage 1 (P1)) were then trypsinized and either frozen or passaged into a new coated 6-well culture dish. In the P2 culture, the PLCs were expanded to 80%-90% confluence (~50,000 cells/well).

### Phenotypic characterization of PLCs

PLCs were immunostained for the following cell lineage markers: hepatocyte nuclear factor 4 alpha (HNF4A), Cytokeratin 19 (KRT19), GATA Binding Protein 4 (GATA4), Hepatocyte Specific Antigen (HSA), SRY (Sex Determining Region Y)-Box 17 (SOX17), Forkhead Box A2 (FOXA2), T-Box Transcription Factor (TBX3), Wilms Tumor 1 (WT1), Mesoderm posterior bHLH transcription factor 1 (MESP1), Desmin (DES), C-X-C motif chemokine receptor 4 (CXCR4), alpha 2 actin (ACTA2), vimentin (VIM) and the mesoderm specific antigen (TE-7), as described in S1 File.

### Reprogramming of PLCs into induced pluripotent stem cells

PLCs were reprogrammed into iPSCs with a Sendai virus reprogramming system as described in S1 File.

### Teratoma formation assay

The pluripotency of Li-iPSCs was assessed *in-vivo* by a teratoma formation assay as described in S1 File.

### iPSC differentiation into hepatocyte-like cells (HLC)

Differentiation of iPSC into HLC was performed as previously described [3] with the following modifications: hepatocyte maturation basal medium (L15, 10% fetal calf serum) was replaced by HepatoZYME-SFM medium (ref. 17705–021, Thermo Fisher Scientific).

### RNA-seq and gene expression profiling

RNA-seq and gene expression profiling were performed as described in S1 File.

## Results

### Generation and expansion of human primary liver cells (PLC)

**Fig 1:**
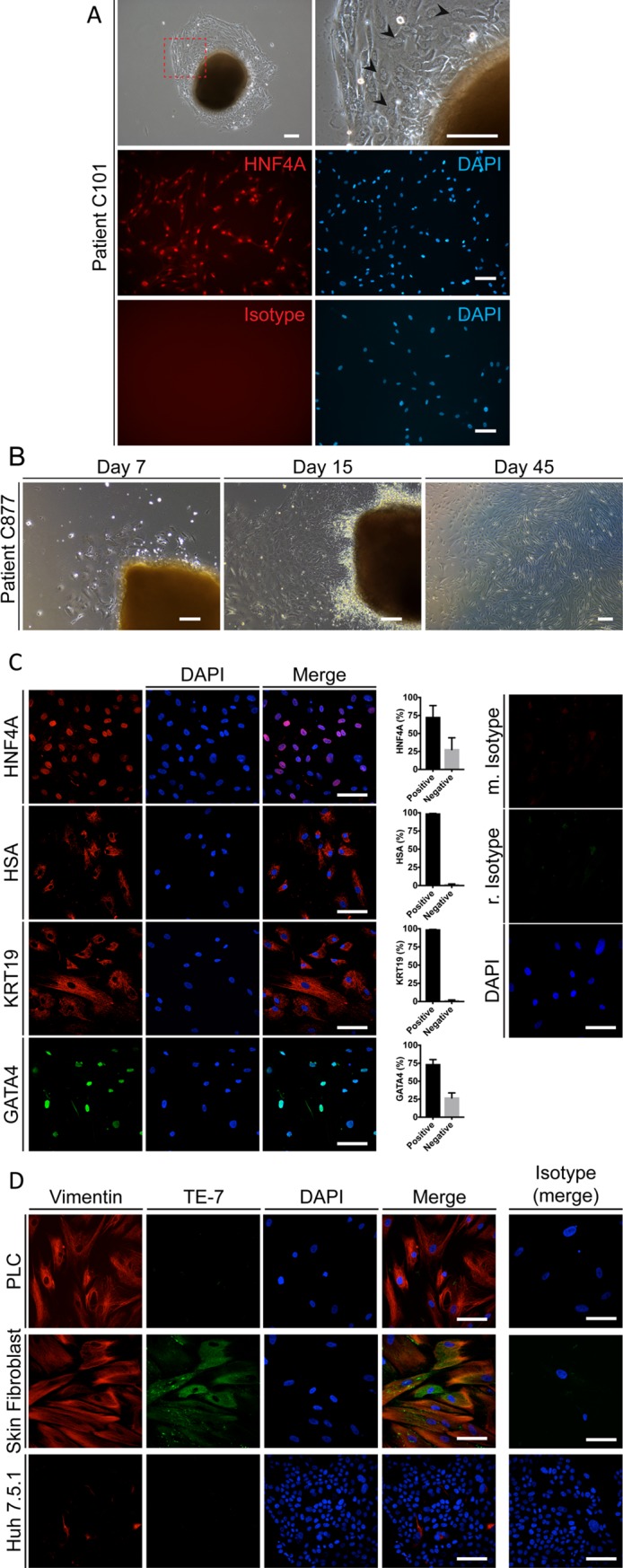
Human primary liver cell (PLC) outgrowth from liver needle biopsy tissue. (A top panel) PLCs surrounding a small piece of a liver biopsy after 15 days in culture (patient C101). Black arrows indicate binucleated cells (scale bars 150μm). (A middle panel) PLCs (patient C101) express HNF4A after 15 days in culture. (A lower panel) Isotype control. Outgrown PLCs were trypsinized and replated on a thin layer-coated culture dish, fixed 24 hours later and subjected to HNF4A specific immunofluorescence staining. Red, HNF4A; blue, nuclear stain DAPI (scale bar 150μm). (B) Bright field images of outgrowing PLCs at day 7, day 15 and day 45 of tissue culture (scale bar 150μm). (C) Endoderm derived cell marker expression in PLCs (patient C101) after 45 days of in vitro culture. Red, HNF4A, HSA and KRT19 from top to bottom, respectively; Green, GATA4; Blue, DAPI nuclear staining; scale bar 100μm. Isotype controls are shown on the right panel; scale bar 100μm. The frequency of the cells positive and negative for the corresponding marker is shown as the percentage of the total number of cells analyzed. (D) TE-7 mesoderm antigen- and vimentin-specific immunofluorescence in PLCs (patient C101), human skin derived fibroblasts and the hepatoma cell line Huh 7.5.1 after 45 days in culture. Red, VIM; Green, TE-7; Blue, DAPI nuclear staining. Right Panel, Isotype control. Scale bars, 100μm.

To obtain enough human primary liver cells (PLCs) from small pieces of liver needle biopsies for subsequent reprogramming into iPSCs, we developed a primary liver cell expansion procedure. Needle biopsy tissue was cut into small pieces that were placed in a culture dish. Over the first week in culture, a growing number of PLCs were observed outside the liver biopsies. These cells attached to the culture dishes and formed a cell halo around the biopsy piece (Fig 1A top panel). During the first 15 days, PLC cultures were mostly composed of epithelial-like, often binucleated cells (Fig 1A top panel) expressing the hepatocyte specific transcription factor HNF4A (Fig 1A middle panel). After 15–20 days of culture, the cell halo was comprised of up to 300 cells and the biopsy piece was removed and discarded. The adherent PLCs were trypsinized and frozen for long-term storage or further expanded as described in the material and methods.

Upon further passaging the morphology of the PLCs progressively changed from an epithelial- to a mesenchymal-like shape (Fig 1B). Nevertheless, after 50 days in culture, more than 70% of the PLCs stained positive for the transcription factors HNF4A and GATA4 (Fig 1C), and virtually all the PLCs were positive for HSA and KRT19 (Fig 1C).

These results suggested that the expanded PLCs were of the endoderm lineage. Interestingly however, co-staining of PLCs for the endoderm markers and mesenchymal markers, such as Vimentin and ACTA2 (S2 Fig) showed that these PLCs were also positive for these mesenchymal markers. To exclude that PLCs were derived from the mesoderm lineage (i.e. fibroblasts), the cells were analyzed for the expression of TE-7 which has been previously used to identify mesoderm-derived cells [14]. As expected, skin fibroblasts were strongly positive for TE-7 while the hepatoma cell line Huh-7 did not show any expression of TE-7 (Fig 1D). Importantly, PLCs did not show any TE-7 signal, although they were positive for vimentin (Fig 1D).

Additionally, PLCs stained positive for the transcription factors SOX17, FOXA2 and TBX3 (i.e. endoderm and hepato-pancreatic progenitors markers [15, 16]) (S1A Fig); and were negative for the mesoderm markers WT1, MESP1, DES and CXCR4 [17–20] (S1B Fig).

Taken together, these results suggest that PLCs were not derived from the liver fibroblasts but rather from cells of endodermic origin, and that these cells undergo an epithelial to mesenchymal change during *in vitro* culture and expansion. This hypothesis is further supported by transcriptomic analysis showing a strong enrichment of an EMT gene signature in PLCs compared to the originating liver samples (S3 Table).

Taken together, these results suggest that PLCs of endoderm origin could be derived from small pieces of liver needle biopsies.

### Reprogramming of PLCs into liver-derived induced Pluripotent Stem Cells (Li-iPSCs)

We next investigated whether PLCs derived from liver biopsies could be reprogrammed into induced pluripotent stem cells (Li-iPSCs). Cell reprogramming requires transient expression of the OCT3/4, SOX2, KLF4 and cMYC factors [21]. For cell reprogramming we choose to explore the non-integrative Sendai virus (SeV) based expression system since it allows for transient expression in the absence of any modifications of the host cell genetic background [22]. We first confirmed that PLCs can be infected with SeV expression vectors using a GFP expression vector (EmGFP SeV, S3 Fig). We then transduced PLCs from 8 different donors (S1 Table) with SeV vectors expressing the reprogramming factors. Twelve to eighteen days post transduction, we observed growing colonies from 4 transduced PLC lines (S4A and S4B Fig). Emerging colonies stained positive for the surface pluripotency marker TRA-1-60 [23], whereas surrounding PLCs did not (S4C and S4D Fig), suggesting that the emerging colonies were indeed formed by iPSCs. Three of the lines could be propagated, whereas one line stopped growing for unknown reasons.

**Fig 2:**
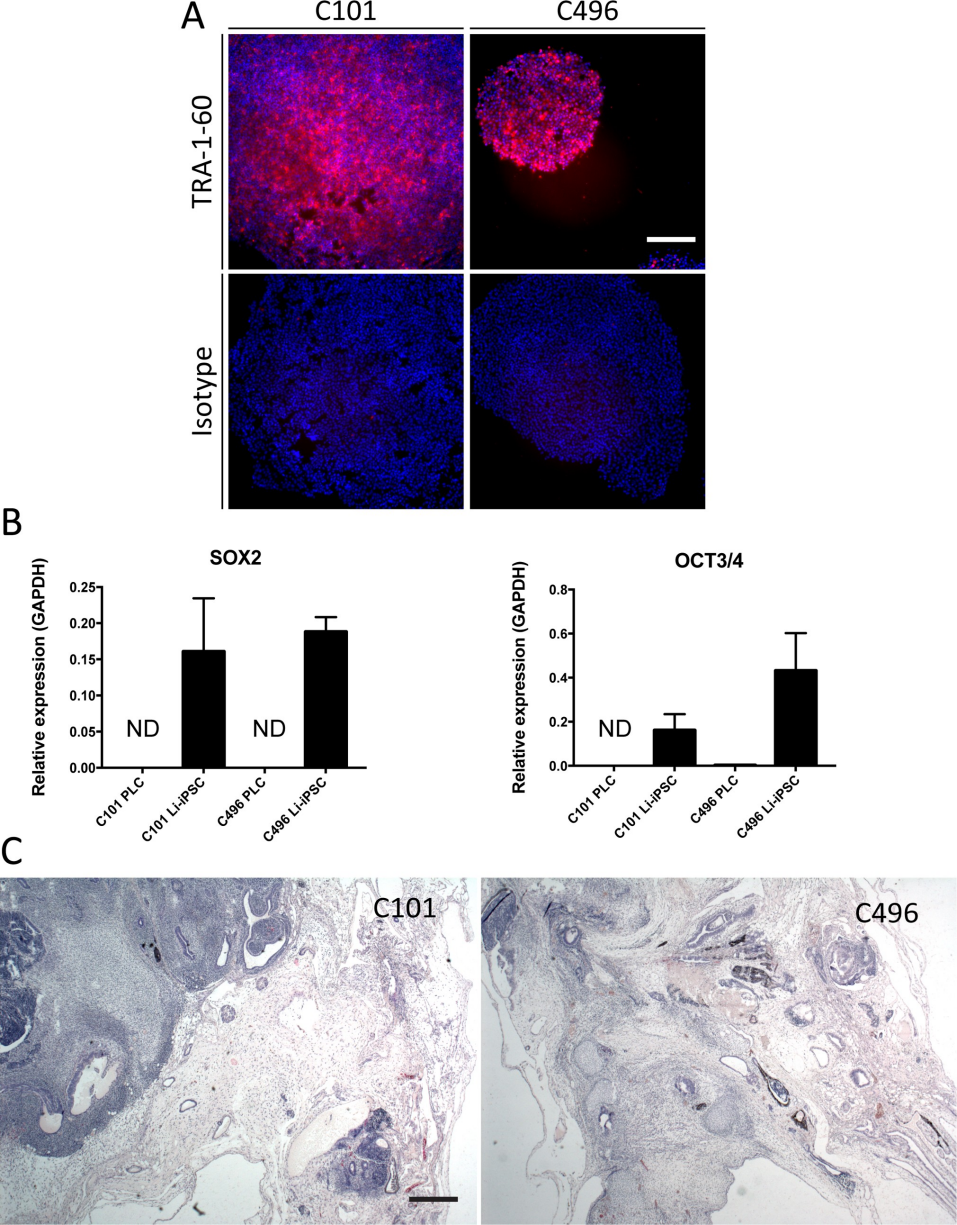
Pluripotency of Li-iPSCs. (A) Surface pluripotency marker TRA-1-60 specific or isotype IgG life immunofluorescence analysis of Li-iPSC colonies derived from patients C101 and C496 (scale bar 250μm). (B) Pluripotency gene (SOX2 and OCT3/4) expression in PLCs and Li-iPSCs derived from patients C101 and C496 was quantified by RT-qPCR using total cellular RNA. Shown is the relative expression of the transcripts compared to GAPDH (mean +/- SD; ND = not detectable). (C) Hematoxylin and eosin staining of formalin fixed and paraffin embedded sections from teratomas isolated from NOD-SCID mice 50 days after subcutaneous injection of Li-iPSCs from patients C101 or C496 (scale bar 500μm).

Subsequently, iPSC colonies were selectively dissociated from the PLCs, purified by anti-TRA-1-60 magnetic bead selection and cultured in maintenance medium for at least 20 passages. The expression of the virally transduced xenogenes was transient in 2 iPSC lines (C101 and C496), but persisted in one line (C877). The 2 xenogene-free lines (C101 and C496) were used for further experiments. Successful purification of the iPSC colonies was confirmed by TRA-1-60 expression (Fig 2A) and by expression of the pluripotent cell marker genes SOX2 and OCT3/4 [24] in Li-iPSCs (Fig 2B).

Finally, we determined the functional pluripotency of our Li-iPSC lines using teratoma forming assays in mice [25, 26]. Immunodeficient NOD-SCID mice were subcutaneously injected with 10^6^ Li-iPSCs and the growth of tumors was monitored over 7 weeks (S5A Fig). Tumors harvested at week 7 showed the morphological hallmarks of teratomas since they contained tissue derived from all the three germinal layers (Fig 2C, S5B and S5C Fig). Taken together, these results demonstrate that our methodology allows the generation, purification and maintenance of Li-iPSC lines from patient-derived PLCs.

### Li-iPSC differentiation into hepatocyte-like cells (HLCs)

**Fig 3:**
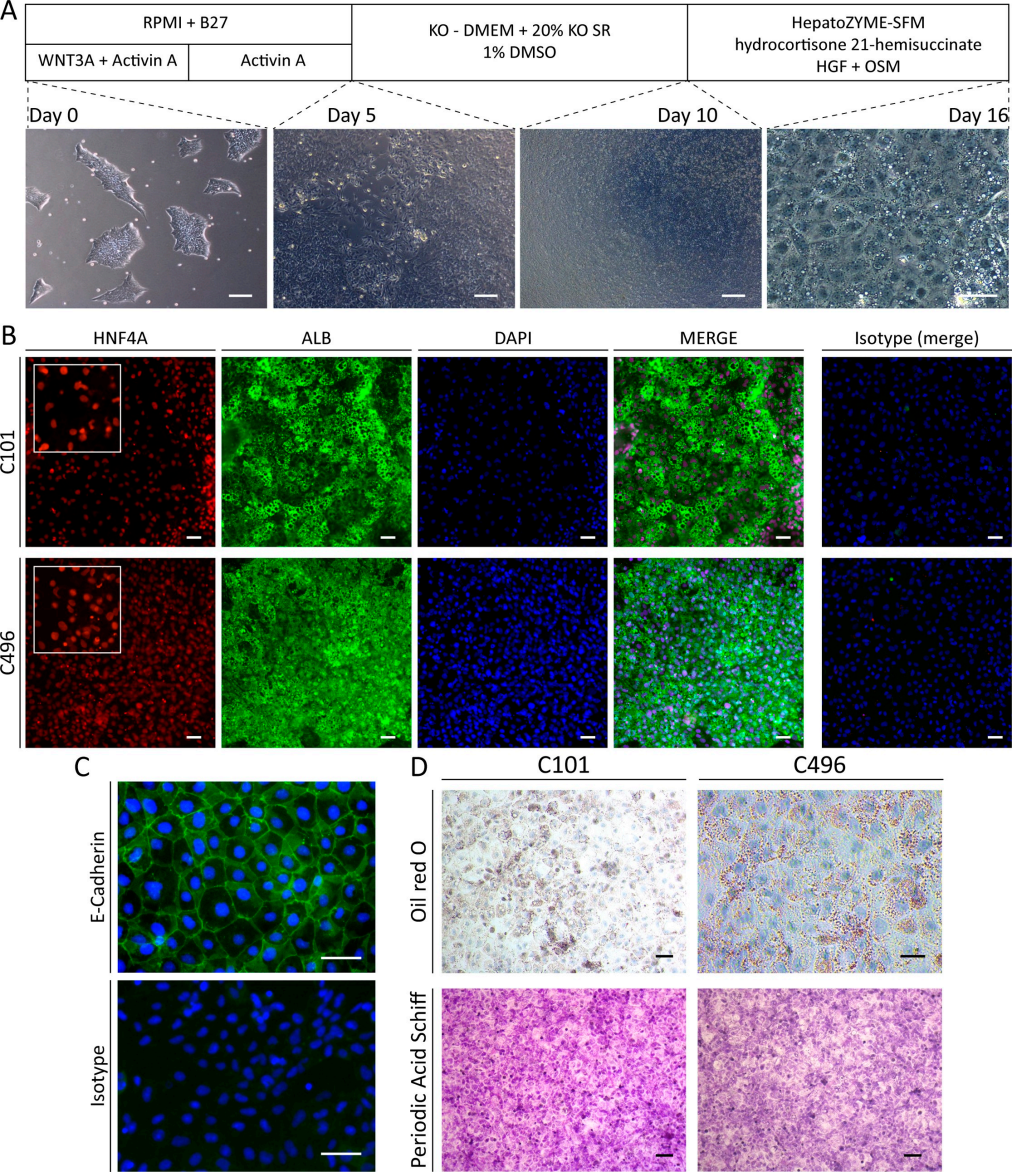
Li-iPSC differentiation into functional Li-HLCs. (A) Schematic illustration of the HLC differentiation procedure together with representative brightfield images taken at day 0, 5, 10 (scale bars = 250 μm) and 16 (scale bar 50 μm) during Li-HLC differentiation. (B) Li-HLCs at the end of differentiation protocol (day 16) co-stained positive for the hepatocyte specific markers HNF4A and Albumin, and for the pan epithelial marker E-Cadherin (C) by immunofluorescence analysis compared to isotype IgG control (scale bar 50 μm). (D) Fully differentiated Li-HLCs are functional with respect to the accumulation of lipids and glycogen as determined by Oil red O and Periodic Acid Schiff (PAS) staining (scale Bars 50 μm). Image quantification is described in S1 File.

We next assessed whether Li-iPSC can be differentiated into HLCs (thereinafter called Li-HLCs) and applied a well-described stepwise differentiation protocol as pioneered by Sullivan et al. [3] and outlined in Fig 3A. Loss of iPSC morphology and acquisition of hepatocyte morphology (Fig 3A) was comparable to previously published work using fibroblast derived iPSCs [3]. Importantly, up to 80% of the cells expressed the hepatocyte markers HNF4A[27] and Albumin at the end of the differentiation procedure (day 16) (Fig 3B). Furthermore, Li-HLCs also expressed E-Cadherin (Fig 3C), whose expression in the liver is restricted to hepatocytes.

To functionally characterize the Li-HLCs, we assessed lipid production and storage, and the production of glycoproteins and glycogen in Li-HLCs by Oil-red-O and PAS staining respectively (Fig 3D).

**Fig 4:**
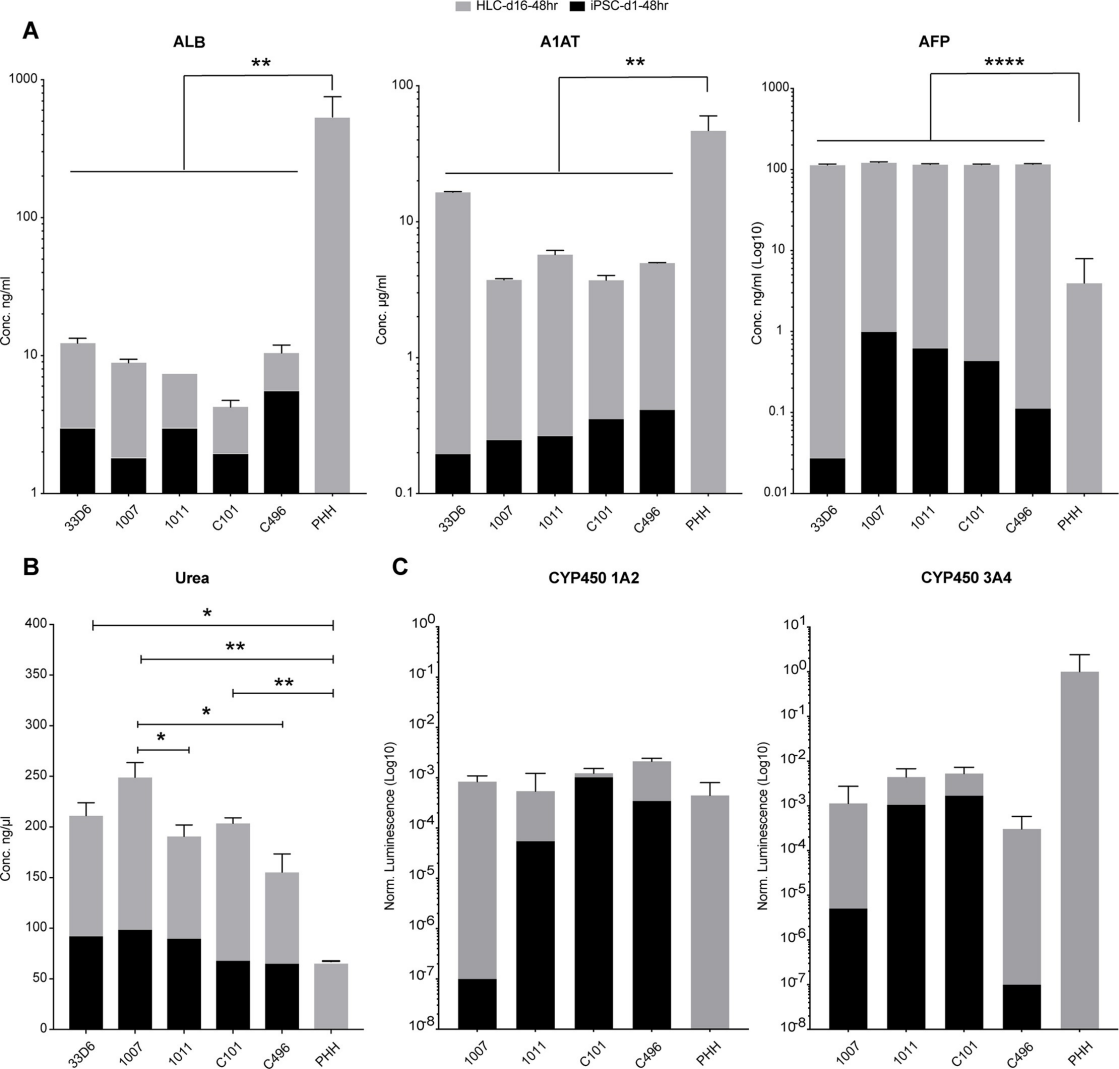
Albumin, Alpha-1 antitrypsin, Alpha-fetoprotein, Urea secretion and Cytochrome P450 activity by iPSCs, HLCs and PHH. iPSCs were seeded on thin layer coated multi-well plates. After 48 hours the medium was collected for analysis. HLCs were differentiated for 16 days, and supernatants were collected 48 hours after the last medium change. PHH isolated from partial hepatectomy tissue were seeded on collagen coated multi-well plates. Fresh medium was added 12 hours after seeding and collected for analysis 48 hours later. All assays were performed following the manufacturer's instructions. Albumin, Alpha-1 antitrypsin and Alpha-fetoprotein were quantified using the included protein standards. Cytochromes P450 1A2 and 3A4 activities were quantified by incubating the HLCs with cytochrome-specific substrates and measured by a luminogenic assay. The values were normalized by the cell number and visualized on a Log10 scale. Differences between HLCs and PHH were analyzed by one-way ANOVA and statistically significant differences are indicated. * = p ≤ 0.05, ** = p ≤ 0.01, **** = p 0.0001.

In addition, we quantified the secretion of Albumin, Alpha-fetoprotein (AFP), Alpha 1-antitrypsin (A1AT) from iPSCs, HLCs after 16 days of differentiation and primary human hepatocytes (PHH) as described in S1 File. As expected, albumin, AFP and A1AT secretion increased upon differentiation of both Li-iPSCs and Fi-iPSCs (Fig 4A). Secretion of albumin and A1AT varied among donors, but compared to the more than 10-fold higher secretion levels in PHH, there was no significant difference between fibroblast and liver derived HLCs. AFP secretion was virtually the same for all HLCs and, as expected, significantly higher than in PHH.

Furthermore, HLCs obtained from differentiation of Li-iPSCs and Fi-iPSCs showed a >2-fold increase in urea secretion reaching levels that were higher than that observed in PHH (Fig 4B). For urea secretion, we did observe small but statistically significant differences between two Fi-iPSC derived HLCs (1007 and 1011) and between the Fi-iPSC and Li-iPSC derived HLCs 1007 and C496, respectively (Fig 4B).

Finally, we evaluated cytochrome P450 1A2 and 3A4 activities in Fi-HLCs and Li-HLCs after 16 days of differentiation and in PHHs as described in the S1 File. For both the cytochromes, we could observe an increased activity in the HLC lines compared with the originating iPSC lines. However, we could not find any significant differences between the HLCs or between HLCs and PHHs (Fig 4C).

Taken together, these results demonstrate the successful differentiation of Li-iPSCs into Li-HLCs and that at the functional level, there are only marginal differences between cell lines derived from different tissues.

### Transcriptional profiles of liver biopsies, PLC, Li-iPSCs and Li-HLCs

**Fig 5:**
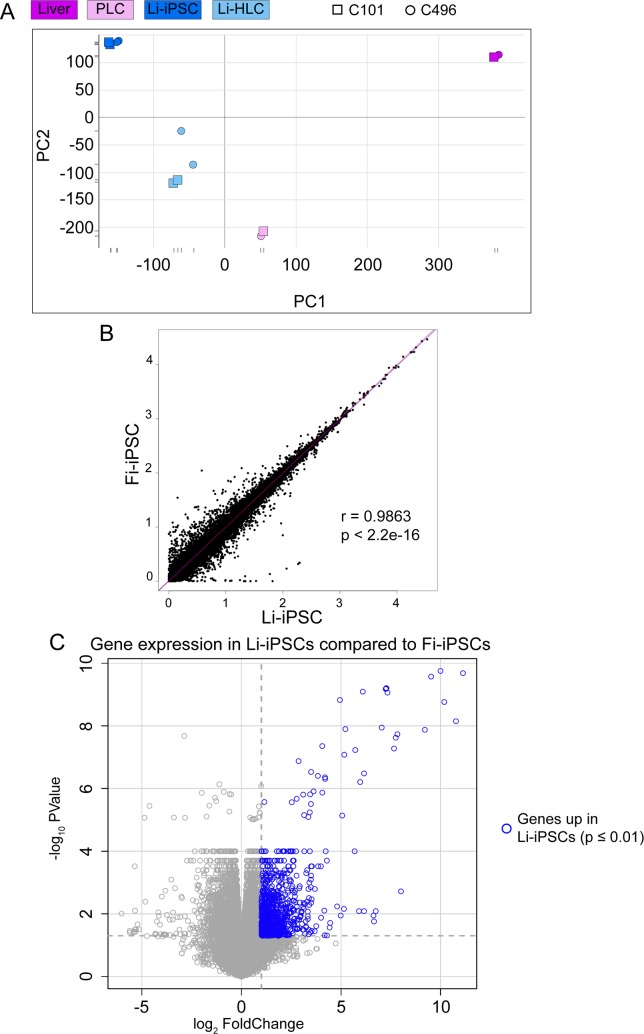
Principal Component Analysis, correlation analysis and differential gene expression analysis in Li-iPSC and Fi-iPSC samples. (A) Principal Component Analysis using the liver, PLC, Li-iPSC and Li-HLC transcriptomes (S3 Table). Graphical representation of PC1 and PC2 based sample clustering. (B) High correlation between the Li-iPSC and Fi-iPSC transcriptional profiles. Pearson's correlation (r- and p-value are indicated) was calculated between the average of all Li-iPSC and all Fi-iPSC transcriptomes. (C) Volcano plot representing the differential gene expression analysis of Li-iPSCs vs. Fi-iPSCs (Limma contrast for grouped Li-iPSCs vs. grouped Li-HLCs, FC ≥ 2, p-value ≤ 0.01). Genes significantly upregulated in Li-iPSCs compared to Fi-iPSCs are shown in blue.

To assess similarities and differences in gene expression between PLCs, Li-iPSCs, Li-HLCs and the originating liver biopsies, we performed RNAseq analysis on polyA-enriched mRNA (S4 Table). To account for culture variations, RNAseq was performed on duplicated Li-iPSC cultures and two Li-HLC differentiations thereof for each patient. The overall relationship between the samples was determined by principal component analysis (PCA). Liver, PLC, Li-iPSC and Li-HLC samples separated into distinct clusters (Fig 5A). As expected, the liver and the Li-iPSC clusters were separated by the largest distance, while Li-HLCs and PLCs were positioned in between. The Li-HLC cluster was less compact than the others, suggesting some variability between individual samples and differentiation conditions. The PLC cluster was located between the liver and the Li-iPSCs based on PC1 and relatively close to the Li-HLCs based on PC1 and PC2 suggesting some transcriptional similarities between PLCs and Li-HLCs. Of note, the liver cluster was clearly set apart from the other clusters in PC1, demonstrating that all cultured cells have significantly changed gene expression profiles compared to human liver cells *in vivo*.

### Li-iPSC retain a subtle liver gene expression profile

To determine whether Li-iPSCs would carry a liver transcriptional memory, we compared the Li-iPSC transcriptome with that of fibroblast-derived (Fi)-iPSCs that is not expected to carry any liver specific gene expression. Although a strong correlation at the transcriptomic level could be observed between Li-iPSCs and Fi-iPSCs (Fig 5B), differential gene expression analysis (Limma contrast on grouped Li-iPSCs vs. grouped Fi-iPSCs, FC ≥ 2, p-value ≤ 0.05) revealed 867 unique transcripts, representing 3% of the Li-iPSC transcriptome, that were expressed at higher levels in the Li-iPSCs compared to the Fi-iPSCs (Fig 5C). Pathway analysis revealed that the corresponding genes represented a number of liver-specific pathways (S5 Table) including coagulation, xenobiotic metabolism and bile acid metabolism. Noteworthy, this liver transcriptional memory was detectable despite a massive down-regulation of genes (3645 down-regulated transcripts; Limma contrast in grouped Li-iPSCs vs. grouped livers, FC ≥ 16, p-value ≤ 0.01) associated with liver-specific functions (S6 Table) in Li-iPSCs compared to parental livers. These results suggest that Li-iPSCs still retain a residual transcriptional memory from the parental liver tissues, although they have lost large parts of the parental liver gene expression profile.

### Li-HLCs are not more closely related to liver samples and PLCs compared to fibroblast-derived (Fi-) HLCs

Transcription memory has been shown to influence the differentiation of iPSCs into different cell lineages [13, 28, 29]. Having found that Li-iPSCs retain a limited liver transcriptional memory, we wanted to investigate whether this would translate in a more “hepatocyte-like” phenotype in Li-HLCs. To this end, we performed a transcriptome-wide pairwise correlation analysis (S6 Fig, Spearman’s ranks correlation) between livers, PLCs, Li-HLCs and fibroblast-derived (Fi)-HLCs (S4 Table). As expected, all correlations were highly significant (p ≤ 0.001). The “within class” correlation coefficients ρ between the two liver biopsy samples, the two PLC samples, the two Li-HLC samples and the three Fi-HLC samples were 0.97, 0.97, 0.95 and 0.88–0.97, respectively. Correlation coefficients dropped to 0.6–0.7 between liver samples and all other samples. Of note, the correlation between liver and Li-HLCs was not better compared to Fi-HLCs. The same was true when comparing PLCs with either Li-HLCs or Fi-HLCs, where ρ values ranged from 0.81 to 0.84 (S6 Fig).

**Fig 6:**
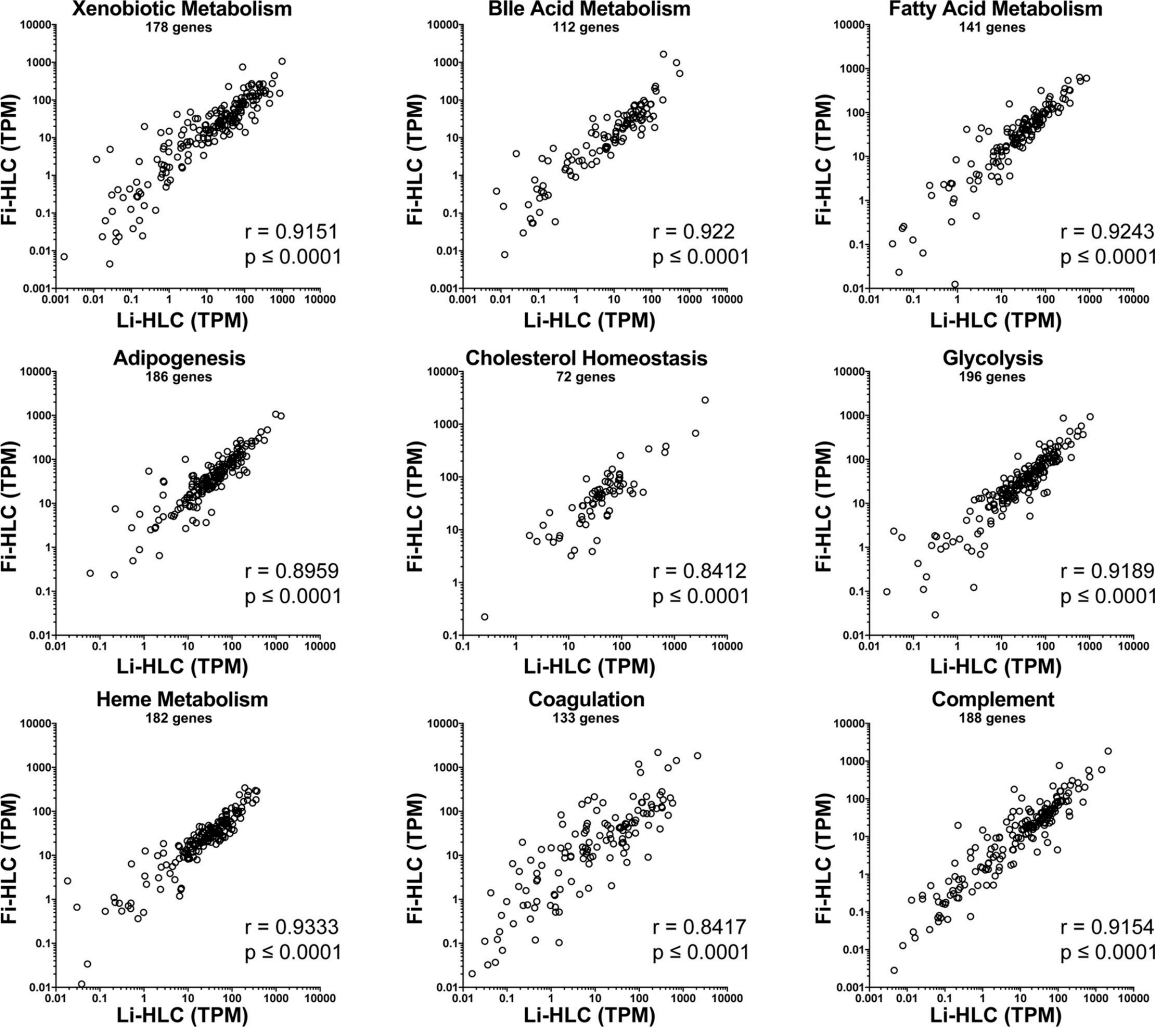
Comparison of liver specific pathway gene expression between Li-HLCs and Fi-HLCs. Shown is the expression of genes included in 9 liver-specific pathways [30] as transcripts per million (TPM). Spearman's ranks correlation (r- and p-value are indicated) was calculated between the average of all Li-HLCs and all Fi-HLCs.

To exclude the possibility that true differences in important liver specific functions would be masked by a majority of non-liver specific genes, we limited the comparison between Li-HLCs and Fi-HLCs on the expression of genes representing nine liver-specific pathways derived from the Molecular Signatures Database (MSigDB 3.0) [30] (Fig 6). For all the pathways we observed a high correlation between Li-HLCs and Fi-HLCs, ranging between 0.84 and 0.93. Together, the gene expression correlation analysis does not provide evidence that the subtle transcriptional memory of Li-iPSCs translate into a more hepatocyte-like transcription profile in Li-HLCs compared to Fi-HLCs.

### Related transcriptional profiles in Li-HLCs and liver

**Fig 7:**
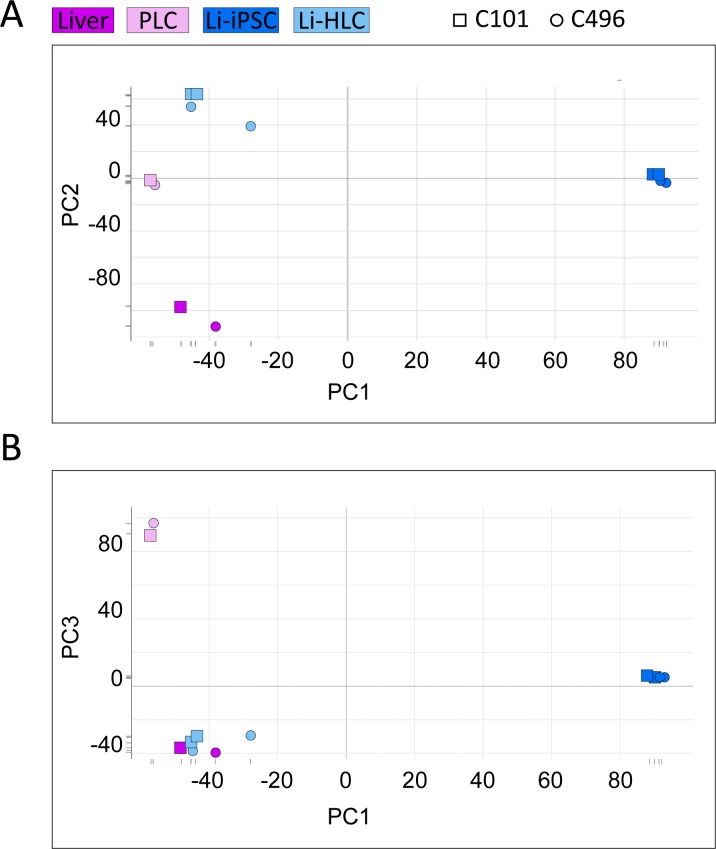
PCA analysis of all samples (S3 Table) based on 3114 genes differentially expressed in Li-HLCs compared to Li-iPSCs (Limma contrast for grouped Li-HLCs vs. grouped Li-iPSCs, FC ≥ 2, p-value ≤ 0.01).

As expected and described above, transcription of many genes associated with liver specific pathways was reduced or absent in Li-iPSCs (S6 Table). Therefore, HLC differentiation is aimed at inducing expression of these genes, to restore the corresponding liver functions. To investigate the transcriptomic changes induced by HLC differentiation, we determined the genes whose expression changed during Li-HLC differentiation. Specifically, we identified 3114 genes differentially expressed in Li-HLCs compared to Li-iPSCs (Limma contrast in grouped Li-HLCs vs. Li-iPSCs, FC ≥ 2, p-value ≤ 0.01). We then performed a PCA using the expression values of these 3114 genes from all cell populations and from the liver samples (Fig 7). In the first dimension of the PCA (PC1), PLCs, Li-HLCs and originating liver samples were together on the left side, and clearly separated from Li-iPSCs. In the second dimension (PC2), Li-HLCs were set apart from the originating liver biopsies, with PLCs and Li-iPSCs in between. Interestingly, Li-HLCs were grouped together with the originating liver biopsies in the third dimension (PC3). Together, these results demonstrate that the gene expression changes induced by the differentiation procedure indeed move the cells towards a “liver-like” expression profile.

### Partial restoration of a liver transcriptional profile in Li-HLCs

**Fig 8:**
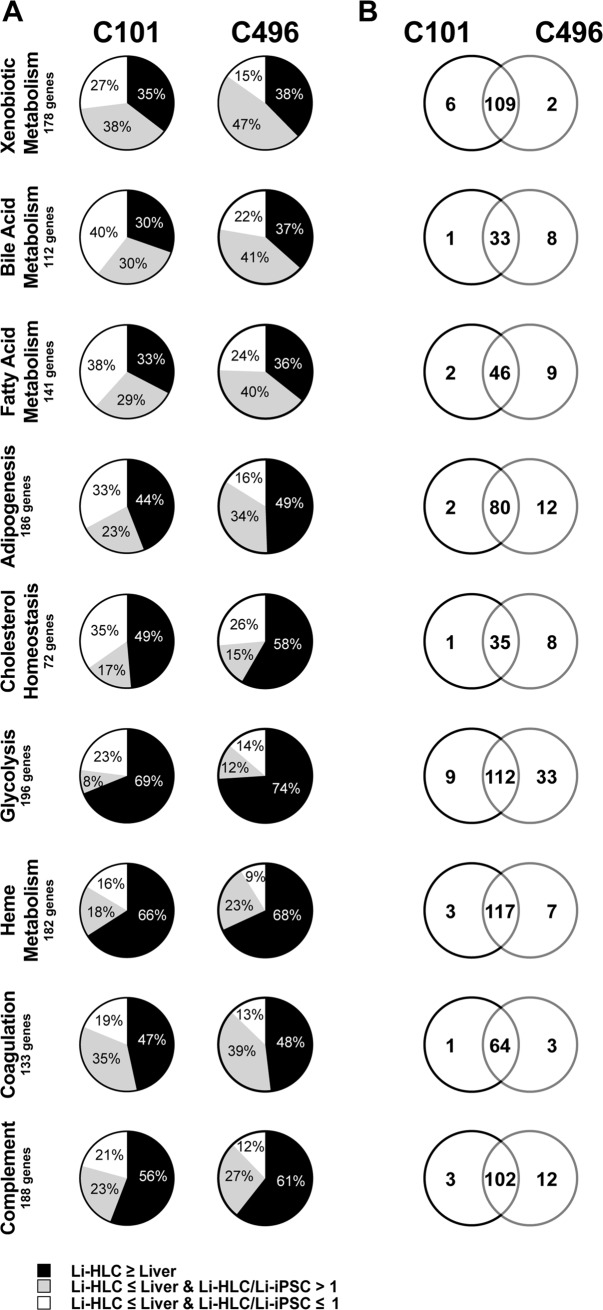
Relative gene expression between Li-HLCs and their originating liver biopsies. (A) Black, gray and white sectors show the percentage of genes whose expression in Li-HLCs relative to biopsies was equal or higher, lower but still induced compared to Li-iPSCs, or lower and not induced, respectively. Genes included in 9 liver-enriched pathways analyzed. (B) Venn diagrams showing the overlap between patients C101 and C496 for genes whose expression in Li-HLCs relative to biopsies was equal or higher.

To specifically determine the efficiency of Li-HLC differentiation, we compared the expression levels of genes representing the nine liver specific pathways (Fig 8 and S7 Table) between Li-HLCs and the corresponding parental livers. Between 30% and 74% of the genes in any given pathway were expressed in Li-HLCs at similar or higher levels compared to the originating liver samples (Fig 8A, S7 Table). These values were not only very similar between the patient C101 and C496 derived samples (Fig 8A), but to a large extent also represented the identical genes (Fig 8B) even though their expression levels varied between patients (S7 Fig). For the genes in each pathway whose expression was significantly lower (Limma contrast in grouped Li-HLCs vs. livers, FC ≤ -2, p-value ≤ 0.01) in Li-HLC compared to liver, we wanted to determine whether their expression was affected at all by the Li-HLC differentiation process. We compared their expression in Li-HLCs with that in the corresponding Li-iPSCs. Between 8% and 47% of the genes in a given pathway were induced in Li-HLCs, but remained significantly lower compared to parental liver (Fig 8A). Finally, the remainder of the genes (between 9% and 40%) in a given pathway were not induced or even down-regulated in the Li-HLCs compared to the Li-iPSCs (Fig 8A).

**Fig 9:**
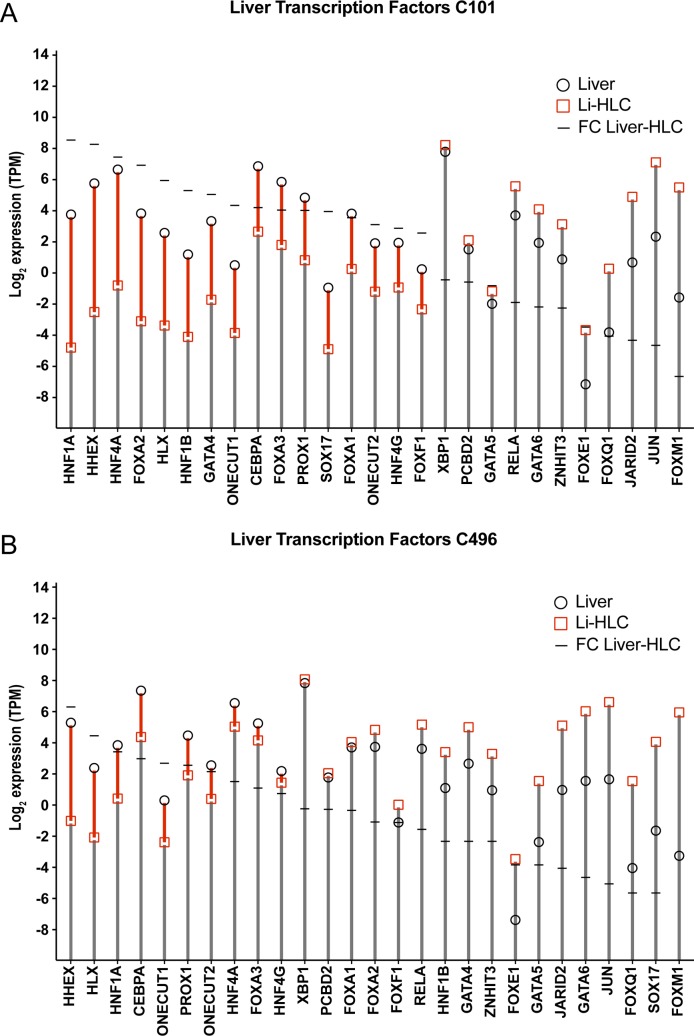
**Comparison of liver-enriched transcription factor gene expression between Li-HLCs and parental liver** for patient C101 (A) and C496 (B). Expression levels of the transcription factor genes in transcripts per million (TPM) are shown for the Li-HLCs in red squares and for the liver in black circles.

This partial restoration of liver gene expression in Li-HLCs could be caused by an insufficient expression of liver-specific transcription factors. We therefore assessed the expression of a set of transcription factors that are involved in liver organogenesis and homeostasis [31] (Fig 9 and S8 Table). Indeed, a number of these transcription factors were expressed at significantly lower levels compared to the corresponding liver sample in both Li-HLC lines (Limma contrast in grouped livers vs. Li-HLCs, FC ≥ 2, p-value ≤ 0.01).

### Liver unrelated genes are induced during Li-HLC differentiation

**Fig 10:**
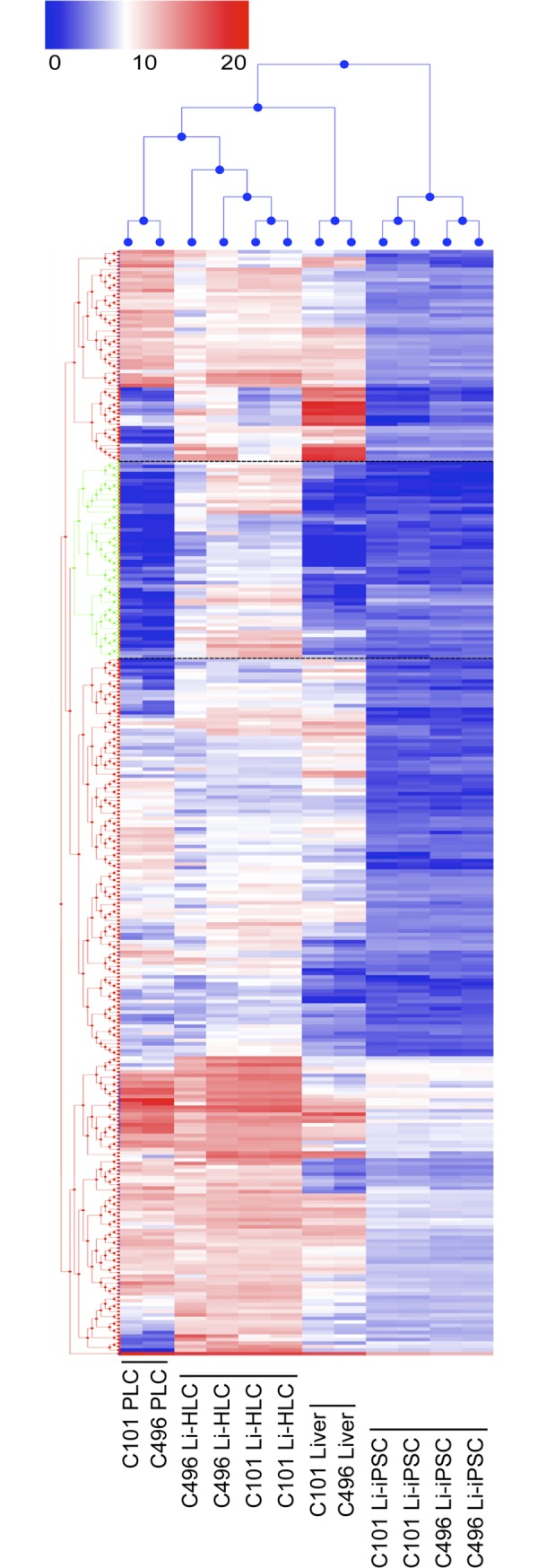
Hierarchical clustering analysis (Manhattan distance metric and average linkage algorithm) of the 10% top ranking (greatest fold change) genes induced during differentiation of Li-iPSCs into Li-HLCs (Limma contrast for grouped Li-HLCs vs. grouped Li-iPSCs, FC ≥ 4, p-value ≤ 0.01). Coloring reflects relative gene expression from low to high (log_2_ expression, 0–21). A cluster of genes overexpressed predominantly in Li-HLCs is framed by dotted black lines.

In addition to an incomplete induction of liver genes in HLCs, the current *in vitro* differentiation protocols could also “accidentally” induce liver-unrelated genes that could in turn inhibit a further differentiation towards bona fide hepatocytes. To explore this possibility, we selected from the 3114 genes that were up-regulated during the differentiation procedure from Li-iPSCs to Li-HLCs the top 10% with the strongest fold change induction, and analyzed their expression in the different samples by unsupervised hierarchical clustering (Fig 10). As expected, samples from the different categories (liver, PLCs, Li-iPSCs and Li-HLCs) cluster together. The analysis also revealed the existence of a group of genes that were overexpressed in Li-HLCs compared to all other samples (Fig 10, genes between dotted lines; listed in S9 Table). A pathway analysis of these genes (S10 Table) revealed that they are associated with tissue and organ morphogenesis, including transcription factors and genes (e.g. MSX2, GATA3, HAND1, and MYH6) apparently not involved in liver organogenesis but rather with craniofacial morphogenesis [32] and development of hematopoietic and cardiac tissue [33–35]. Importantly, induction of these genes was not limited to Li-HLCs, but also occurred in Fi-HLCs (S9 Table). These results show that current hepatic differentiation protocols induce not only liver specific functions, but also liver unrelated pathways, independently of the origin of the iPSCs, which might contribute to the incomplete differentiation into hepatocytes.

## Discussion

The study of liver pathologies *in vitro* still mainly relies on PHH cultures considered to best represent authentic hepatocyte functions [36]. However, batch-to-batch variability of PHHs, their tendency for dedifferentiation in culture over time, and the limited supply severely restrict their usability [37]. Recently, ESC- and iPSC-derived HLCs have emerged as a promising alternative *in vitro* model for hepatocytes [38, 39]. With the current study we wanted to extend the iPSC/HLC system to the usage of surplus needle biopsy tissue obtained during routine liver biopsy in the clinical setting, with the aim to establish a personalized *in vitro* hepatocyte model system.

Since the perfusion-based methods to produce primary liver cells (i.e. PHH) from big resection pieces [40] cannot be applied to needle biopsies, we established a method that relies on spontaneous outgrowth of human PLCs from small pieces of liver tissue. Given the limited number of cells in the early cell outgrowth, this method did not allow for a specific cell type selection (e.g. hepatocyte cell sorting). Furthermore, due to methodological constraints, it was also very difficult to perform a lineage tracing experiment targeting only the hepatocytes within the liver biopsy tissue. However, although we were not able to univocally identify the cell of origin of PLCs used to produce Li-iPSCs, we presume they derived from endoderm, based on the following evidences. Outgrowing PLCs initially were of epithelial-like morphology and expressed the hepatic transcription factor HNF4A. At this stage we could not detect any morphological difference, nor differences related to proliferation, cell cycle, outgrowth etc. between PLCs derived from different patients. During *in vitro* culture however, PLCs increasingly displayed a mesenchymal-like morphology. This is most likely not due to an overgrowth of the cultures by rare mesenchymal cells originating from the liver biopsies, but rather results from epithelial-to-mesenchymal transition (EMT). Indeed, the expanded PLCs, although being positive for the fibroblast markers Vimentin and Smooth Muscle Actin (ACTA2), were negative for the mesoderm markers WT1, MESP1, DES, CXCR4 and for the mesoderm-specific antigen TE-7, and positive for the endoderm markers HNF4A, GATA4, KRT19, HSA, SOX17, FOXA2 and TBX3. Together, these results demonstrate that the PLCs are derived from endoderm. Furthermore, the expression of HNF4A and TBX3 also suggests a commitment of these cells towards the hepatocyte lineage because their expression is fundamental for the hepatocyte differentiation during organogenesis [16, 41].

Furthermore, a mesenchymal to epithelial transition of outgrowing cells seems to be very unlikely since our culturing conditions do not provide any of the factors known to be strictly required for MET differentiation *in vitro* (i.e. growth factors nor insulin) [42]. The cell shape changes from epithelial- to mesenchymal-like over time and the co-expression of endoderm specific markers and Smooth Muscle Actin / Vimentin rather suggest that the PLCs undergo an EMT during *in vitro* culture, a hypothesis that is further supported by the enrichment of a transcriptional EMT signature in the PLCs compared to the originating liver samples. Such a scenario would be consistent with previous reports suggesting that liver cells can undergo EMT under specific conditions such as during organogenesis or regeneration (reviewed in [43, 44]). Specifically, Pinzani et al. [45] proposed that liver hepatocytes could undergo EMT to escape from a hostile microenvironment. Thus, it seems conceivable that transition and expansion in a culture dish would constitute such a "hostile environment" triggering an EMT in the outgrowing PLCs. Future studies might reveal the precise changes that occur during expansion of PLCs *in vitro*.

Using a non-integrative Sendai virus-based system [22] for reprogramming, we could derive Li-iPSCs from PLCs devoid of any modifications of the patient genetic background due to integration of viral vectors. This is important with respect to the development of personalized medicine approaches, as it has been well described that the patient genetic background plays an important role in many liver diseases (e.g. alpha-1 antitrypsin deficiency, hemochromatosis, Wilson’s disease and response to HCV treatment; [46] and reviewed in [7, 47, 48]. The reprogramming efficiency observed for C101 and C496 PLCs was comparable with the efficiency observed for the reprogramming of other cell types (e.g. skin or blood) using the same methodology [49]. The overall low success rate of 3 out of 8 iPSC derivations is consistent with the observations reported for reprogramming PHHs into iPSCs by Sendai vectors [12, 50]. Future studies will be required to explore the possibility that variations in culture conditions might increase the reprogramming efficiency as has been suggested by Heslop et al. [12].

The availability of the originating liver tissue allowed for the first time to directly compare the transcriptome of the originating tissue (i.e. liver) with that of the Li-iPSCs and Li-HLCs. We found that reprogramming of liver cells into Li-iPSCs leads to a widespread loss of liver-specific gene expression. This is not surprising, considering that the gain of pluripotency has been described to be associated with a drastic chromatin rearrangement towards an open chromatin stage (reviewed in [51]) and a down-regulation of differentiation genes [52]. Nevertheless, we could identify genes expressed in Li-iPSCs associated with liver-specific pathways that were absent in Fi-iPSCs, suggesting that the Li-iPSCs maintained a subtle liver transcriptional memory. This finding is consistent with previous reports of iPSCs reprogrammed from other tissues [28, 29]. However, the impact of transcriptional and epigenetic memory on the differentiation of iPSCs into different cell types remains controversial as another study observed a loss of epigenetic memory after repeated passaging of the iPSCs [53]. We demonstrate here that Li-HLCs do not show a better correlation with the originating liver than Fi-HLCs. This suggests that with the current differentiation protocols, the tissue of origin of the iPSCs has little impact on the transcriptional profile of the differentiated HLCs. Together with recent reports [12, 54], one of which showing that genetically matched iPSCs, derived from fibroblasts or PHHs, yielded very similar HLCs [12], our results support the hypothesis that factors other than the tissue of origin of the iPSCs determine HLC differentiation efficiency.

It has been reported previously that iPSC derived HLCs do not completely recover the gene expression and functions of hepatocytes in the liver [55]. However, these conclusions were so far hampered by the fact that HLCs could not be directly compared to the liver tissue of the same genetic background (i.e. patient). In the present study we could for the first time directly compare individual gene expression in Li-HLCs and their corresponding originating liver tissue. This comparison revealed that the expression of 30% to 74% of nine typical liver pathways and/or functions was restored in Li-HLCs to levels similar to those in the parental liver, suggesting that they could fulfill the same functions in HLCs as they do in the liver. For a number of genes in the same pathways (8%-47%), expression was at least partially restored in the Li-HLCs, while a minor fraction of genes (9%-40%) were not induced at all during HLC differentiation, confirming at the individual patient level that HLCs recover only a partial liver-specific gene expression pattern, consistent with previous reports showing an immature hepatic phenotype of HLCs [56]. Looking for a possible cause for this incomplete differentiation, we identified a number of liver-specific transcription factors, whose expression was significantly reduced in HLCs compared to their originating livers. It is noteworthy that these factors are known for their role in liver organogenesis, homeostasis and regeneration (reviewed by [31, 57]), and their absence is associated with severe liver diseases (reviewed by [31]). Hence, we hypothesize that the reduced expression of these transcription factors might be an important reason behind the immature liver phenotype of HLCs. Along those lines, it is intriguing that the reduced expression of these transcription factors in Li-HLCs of patient C101 was apparently reflected in the expression of fewer genes of the liver specific pathways. Thus, it is conceivable that modifications to the currently used HLC differentiation protocols aimed at restoring expression of these transcription factors might improve the functionality of HLCs.

Our analysis revealed another important mechanism responsible for incomplete differentiation of Li-iPSCs into hepatocytes. Cluster analysis of the genes most highly up regulated during differentiation from Li-iPSC to Li-HLCs revealed 56 genes expressed in HLCs, but not in their parental livers. Pathway analysis showed that most of these genes were associated with embryogenesis and organogenesis and even encompassed transcription factors involved in the development of organs other than the liver. Interestingly, this observation also extended to Fi-iPSC derived HLCs suggesting that the induction of these genes is a result of the differentiation procedure.

In conclusion, we describe a new method to derive an unlimited supply of patient- and organ-specific HLCs, starting from a very limited amount of liver needle biopsy tissue. A major challenge for the future use of these cells in pre-clinical and clinical applications is their incomplete differentiation into hepatocytes. In the present study we identify two mechanisms that prevent a full hepatocyte differentiation, (i) weak expression of several transcription factors important for hepatocyte specific gene expression, and (ii) aberrant expression of genes and transcription factors involved in the development of other organs. These novel insights into the gene expression profiles of HLCs form a basis for future development and improvement of iPSC derived HLC model systems.

## Supporting information

S1 Fig Localization of endoderm-derived and mesoderm-derived cell markers in PLCs (patient C101) after 45 days in culture. Frozen PLCs were thawed and cultured for a total of 45 days, trypsinized, replated on thin layer-coated culture dishes, and fixed 24 hours later for immunofluorescence staining. Staining for (A) SRY (Sex Determining Region Y)-Box 17 (SOX17), Forkhead Box A2 (FOXA2), T-Box Transcription Factor (TBX3); (B) Wilms Tumor 1 (WT1), Mesoderm posterior bHLH transcription factor 1 (MESP1), Desmin (DES), C-X-C motif chemokine receptor 4 (CXCR4). Blue, DAPI nuclear staining. (C) Isotype controls. Scale bars 50μm.
(PDF)

S2 Fig Co-localization of endoderm-derived and mesoderm-derived cell markers in PLCs (patient C101) after 45 days in culture. Frozen PLCs were thawed and cultured for a total of 45 days, trypsinized, replated on a thin layer-coated culture dish, fixed 24 hours later and subjected to immunofluorescence staining. (A) Simultaneous staining of Hepatocyte Nuclear Factor 4 Alpha (HNF4A) and Vimentin (VIM), (B) GATA binding protein 4 (GATA4) and Actin Alpha 2 (ACTA2), (C) Cytokeratin 19 (KRT19) and Vimentin (VIM), and (D) Hepatocyte Specific Antigen (HSA) and Vimentin (VIM). Blue, DAPI nuclear staining. (E) Isotype controls. Scale bars 100μm.
(PDF)

S3 Fig EmGFP Sendai reporter virus infection of PLCs. (A) EmGFP signal (green) in uninfected (left panels) PLCs and PLCs infected with EmGFP Sendai virus at MOI of 3 (upper panels) and MOI of 6 (lower panels) for 24 hours (middle panels) and 48 hours (right panels). (B) The frequency of EmGFP positive PLCs before (control) and 48 hours after EmGFP-SeV infection at a MOI of 1, 3 and 6 was determined by FACS analysis. Scale bars = 150μm.
(PDF)

S4 Fig Pluripotent Li-iPSCs colonies derived from reprogramming of PLCs. (A) Cell colonies reminiscent of iPSCs (arrows) emerged in the PLC cultures 12–18 days after infection with the Sendai reprogramming vectors. (B) Bigger magnification of one representative iPSC colony at day 12–18. (C, D) iPSC colonies (encircled by dashed line) were positive for the surface pluripotency marker TRA-1-60 (red) during live staining. TRA-1-60 staining (red) is shown before (C) and afteroverlaying onto the corresponding phase contrast image (D). Scale bars = 250μm.
(PDF)

S5 Fig Li-iPSCs form teratoma in NOD-SCID mice. Teratoma formation assay. NOD-SCID mice (two animals per cell line) have been injected subcutaneous with roughly 10^6^ iPSCs and monitored by palpation to evaluate the teratoma growth. (A) Teratoma growth curve in NOD-SCID mice after subcutaneous injection of two independent Li-iPSC lines (1E6 cells per mouse) each from two different patients. (B) Cystic teratoma collected from the mice shown in (A) at 50 days after Li-iPSC injection. (C) Histologically (H&E staining), Li-iPSC-induced teratoma display tissues derived from the three germinal layers. Ectoderm, pigmented epithelium containing melanin granules (top panels). Endoderm, columnar glandular epithelium (middle panels). Mesoderm, adipose and stromal cells surrounding other tissues (bottom panels) (scale bars 50 μm).
(PDF)

S6 Fig Comparison of liver, PLC, Li-iPSC and HLC transcriptomes. Pairwise correlation analysis (Spearman’s ranks correlation) was performed between each of 9 transcriptome data sets derived from two originating liver biopsy samples, two derived PLC samples, two Li-HLC samples (C101-HLC and C496-HLC) and 3 Fi-HLC samples. The gene expression frequency distribution for each sample is shown as histograms together with the sample name in the panels along the diagonal from the top left to bottom right of the figure. Gene expression correlations plots and Spearman’s correlation coefficient ρ for all sample pairs are shown to the left and right of the frequency distribution histograms, respectively. All correlations were statistically significant with a p-value ≤ 0.001. X- and Y-axes indicate scaled gene expression ((Log_10_TPM)+1).
(PDF)

S7 Fig Comparison of the hallmark xenobiotic metabolism gene set expression pattern between Li-HLCs and parental livers. Expression levels (log_2_) of all the genes in the gene in the Li-HLCs (red squares) and liver (black circles) in patients C101 (top panel) and C496 (bottom panel). The horizontal lines reflect the log_2_-fold change of expression level between the liver and the Li-HLCs. Expression levels are indicated as transcripts per million (TPM).
(PDF)

S1 Table Patient liver primary cell expansion and iPSC reprogramming. (PDF)

S2 Table q-PCR primer list. (PDF)

S3 Table Gene sets significantly enriched in PLCs vs. liver. (PDF)

S4 Table Summary of samples included in the RNAseq experiment for transcriptomic profiling. (PDF)

S5 Table Gene sets significantly enriched in Li-iPSCs vs. Fi-iPSCs. (PDF)

S6 Table Gene sets significantly down-regulated in Li-iPSCs vs. originating livers. (PDF)

S7 Table Liver-specific gene sets (Molecular Signatures Database) expression in Li-HLCs compared to parental livers. (PDF)

S8 Table Gene expression comparison for 27 liver-specific transcription factors between Li-HLCs and parental livers. (PDF)

S9 Table Genes identified by gene and sample clustering as cluster 2. (PDF)

S10 Table GO gene sets significantly enriched in the gene with a divergent expression compared to all other samples analyzed. (PDF)

S1 File Materials and methods. (PDF)
