# Supplementary figures and images for: Liver biopsy derived induced pluripotent stem cells provide unlimited supply for the generation of hepatocyte-like cells
Source: PLoS One. 2019 Aug 29;14(8):e0221762. doi: 10.1371/journal.pone.0221762 (PMC6715171; doi:10.1371/journal.pone.0221762)

S1 Fig.

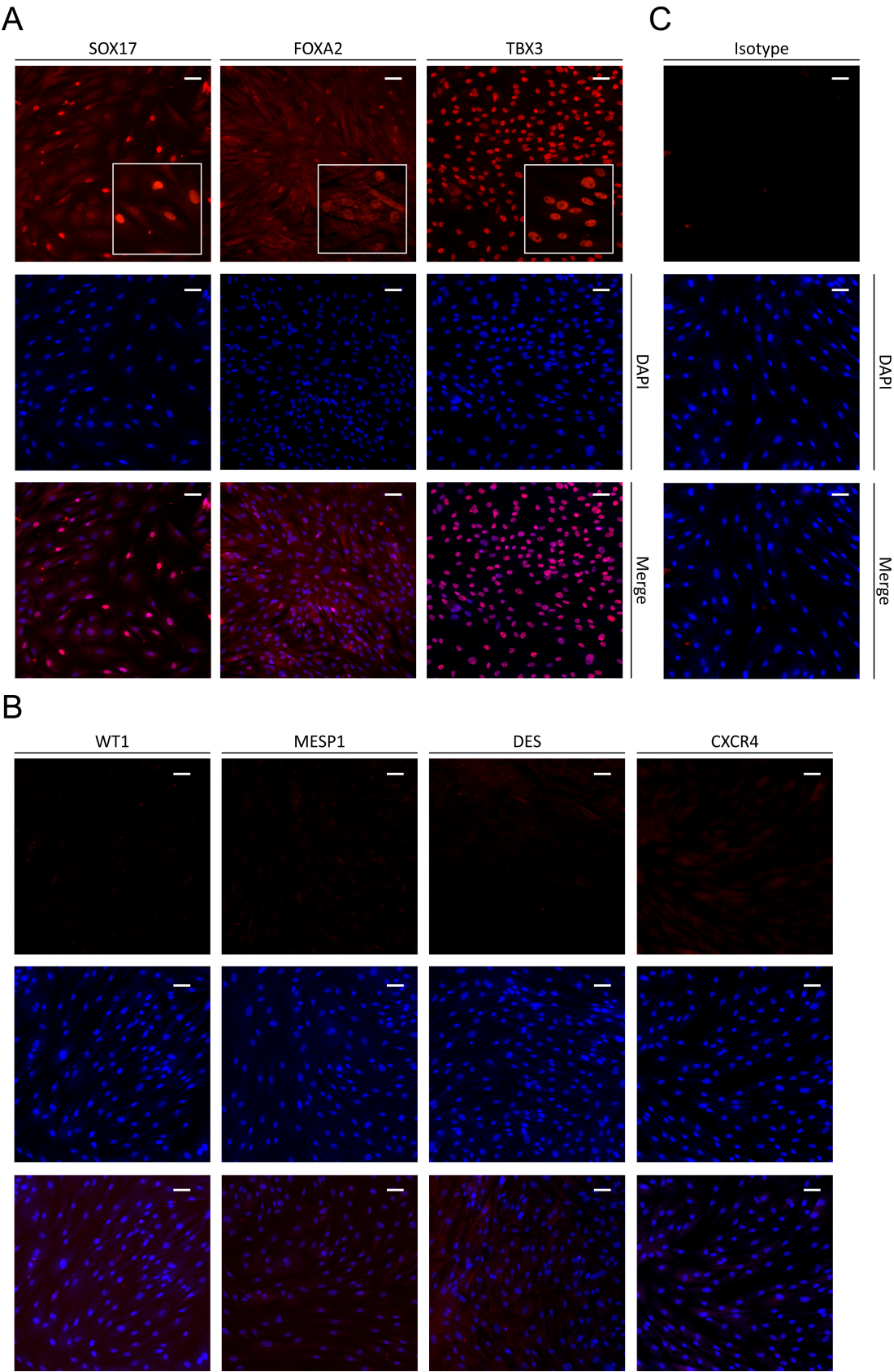

Supplement: S1 Fig — Frozen PLCs were thawed and cultured for a total of 45 days, trypsinized, replated on thin layer-coated culture dishes, and fixed 24 hours later for immunofluorescence staining. Staining for (A) SRY (Sex Determining Region Y)-Box 17 (SOX17), Forkhead Box A2 (FOXA2), T-Box Transcription Factor (TBX3); (B) Wilms Tumor 1 (WT1), Mesoderm posterior bHLH transcription factor 1 (MESP1), Desmin (DES), C-X-C motif chemokine receptor 4 (CXCR4). Blue, DAPI nuclear staining. (C) Isotype controls. Scale bars 50μm. (PDF) [file pone.0221762.s001.pdf]

**S2 Fig.**

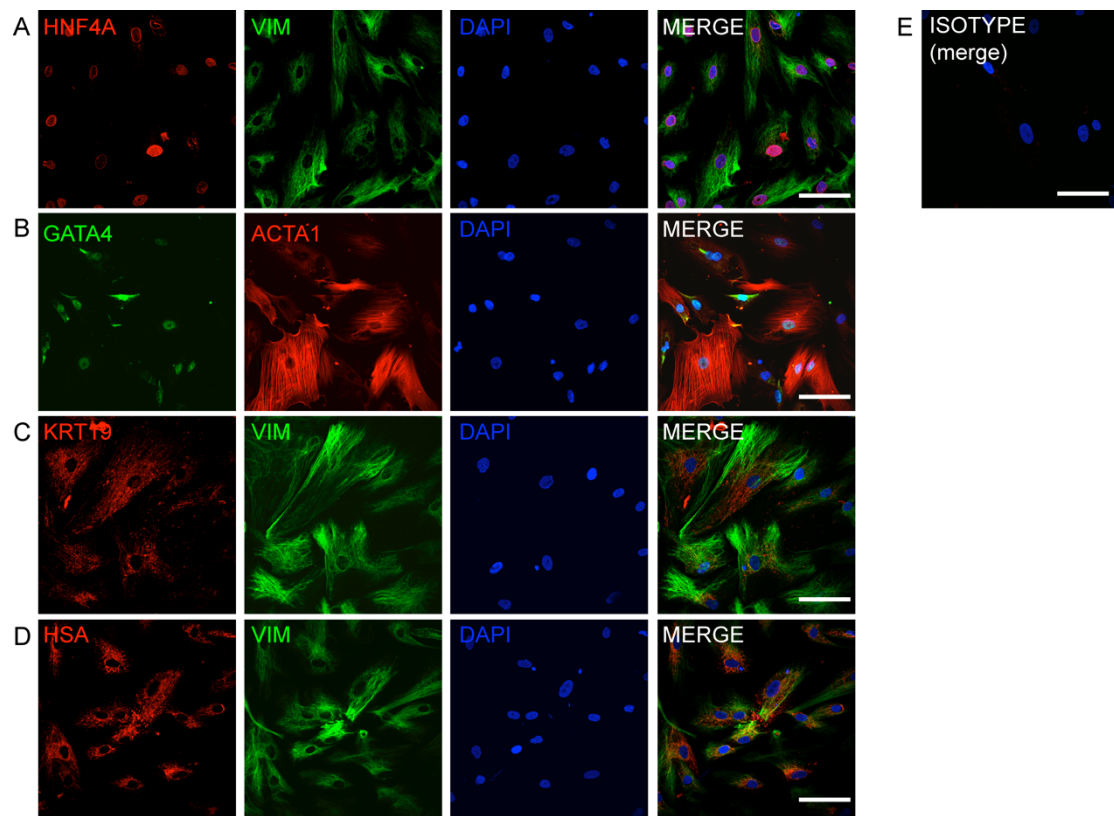

Supplement: S2 Fig — Frozen PLCs were thawed and cultured for a total of 45 days, trypsinized, replated on a thin layer-coated culture dish, fixed 24 hours later and subjected to immunofluorescence staining. (A) Simultaneous staining of Hepatocyte Nuclear Factor 4 Alpha (HNF4A) and Vimentin (VIM), (B) GATA binding protein 4 (GATA4) and Actin Alpha 2 (ACTA2), (C) Cytokeratin 19 (KRT19) and Vimentin (VIM), and (D) Hepatocyte Specific Antigen (HSA) and Vimentin (VIM). Blue, DAPI nuclear staining. (E) Isotype controls. Scale bars 100μm. (PDF) [file pone.0221762.s002.pdf]

S3 Fig.

A

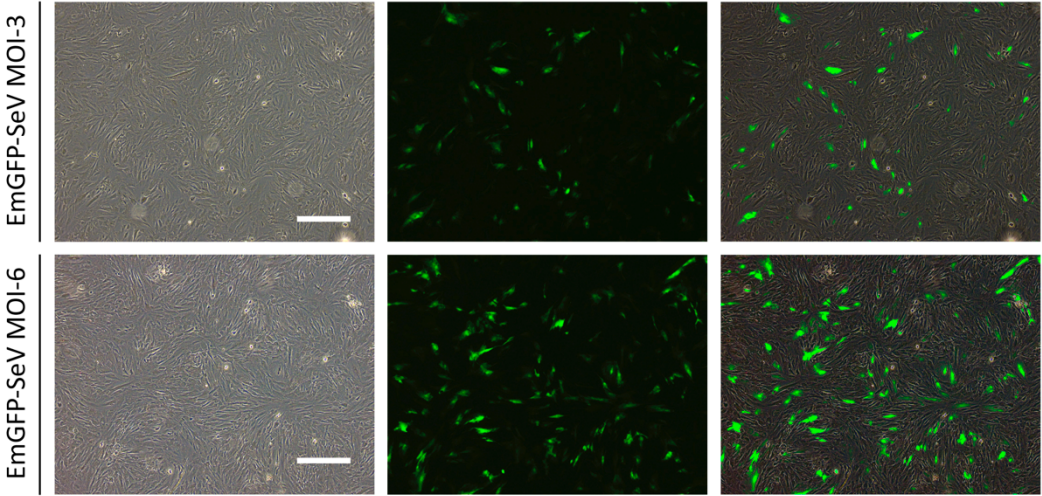

B

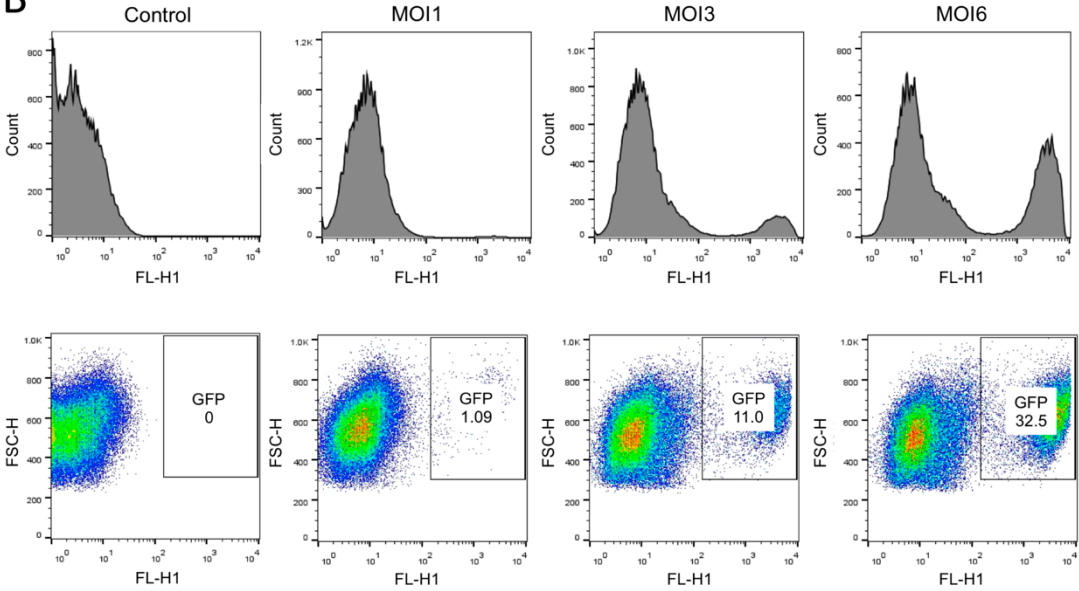

Supplement: S3 Fig — (A) EmGFP signal (green) in uninfected (left panels) PLCs and PLCs infected with EmGFP Sendai virus at MOI of 3 (upper panels) and MOI of 6 (lower panels) for 24 hours (middle panels) and 48 hours (right panels). (B) The frequency of EmGFP positive PLCs before (control) and 48 hours after EmGFP-SeV infection at a MOI of 1, 3 and 6 was determined by FACS analysis. Scale bars = 150μm. (PDF) [file pone.0221762.s003.pdf]

**S4 Fig.**

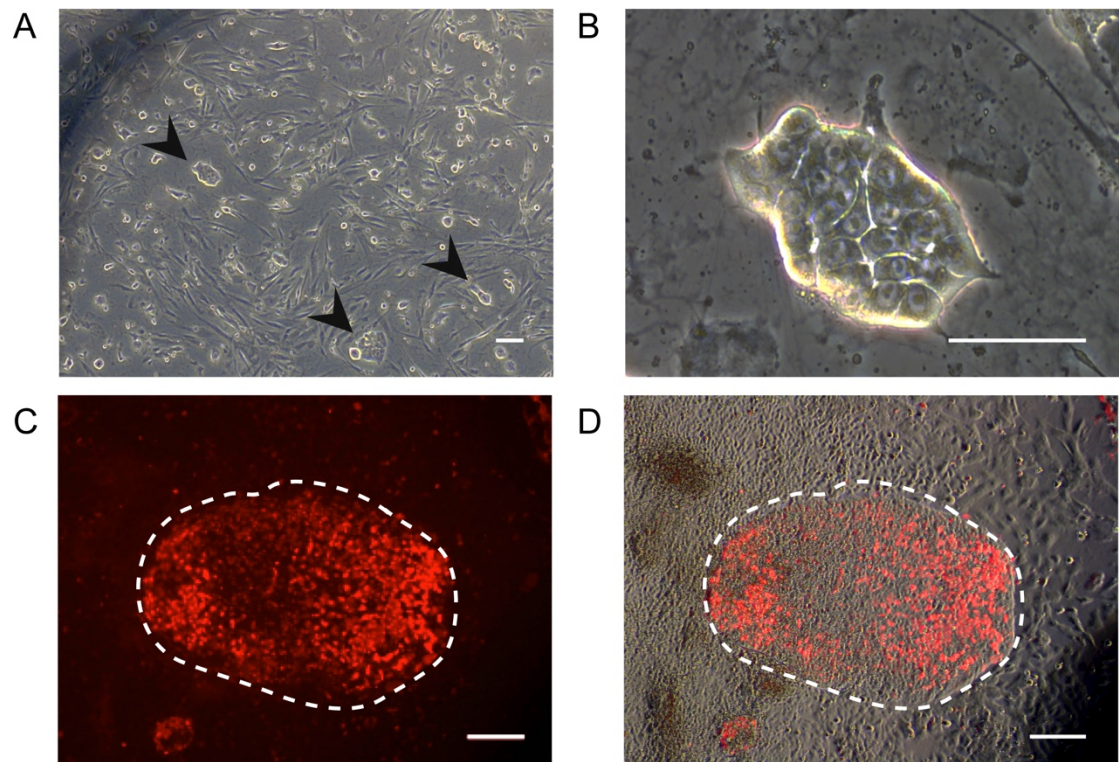

Supplement: S4 Fig — (A) Cell colonies reminiscent of iPSCs (arrows) emerged in the PLC cultures 12–18 days after infection with the Sendai reprogramming vectors. (B) Bigger magnification of one representative iPSC colony at day 12–18. (C, D) iPSC colonies (encircled by dashed line) were positive for the surface pluripotency marker TRA-1-60 (red) during live staining. TRA-1-60 staining (red) is shown before (C) and afteroverlaying onto the corresponding phase contrast image (D). Scale bars = 250μm. (PDF) [file pone.0221762.s004.pdf]

S5 Fig.

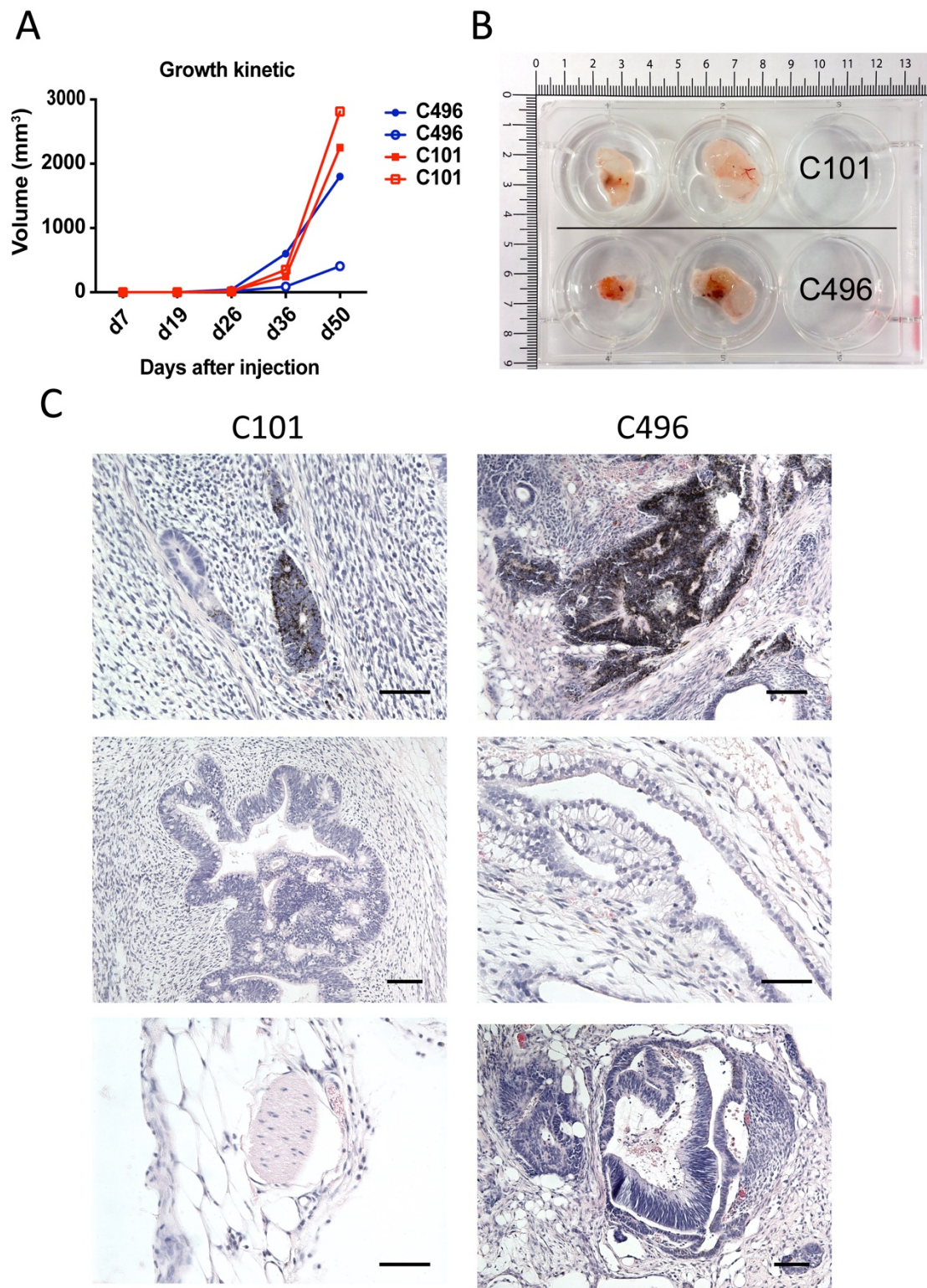

Supplement: S5 Fig — Teratoma formation assay. NOD-SCID mice (two animals per cell line) have been injected subcutaneous with roughly 106 iPSCs and monitored by palpation to evaluate the teratoma growth. (A) Teratoma growth curve in NOD-SCID mice after subcutaneous injection of two independent Li-iPSC lines (1E6 cells per mouse) each from two different patients. (B) Cystic teratoma collected from the mice shown in (A) at 50 days after Li-iPSC injection. (C) Histologically (H&E staining), Li-iPSC-induced teratoma display tissues derived from the three germinal layers. Ectoderm, pigmented epithelium containing melanin granules (top panels). Endoderm, columnar glandular epithelium (middle panels). Mesoderm, adipose and stromal cells surrounding other tissues (bottom panels) (scale bars 50 μm). (PDF) [file pone.0221762.s005.pdf]

S6 Fig.

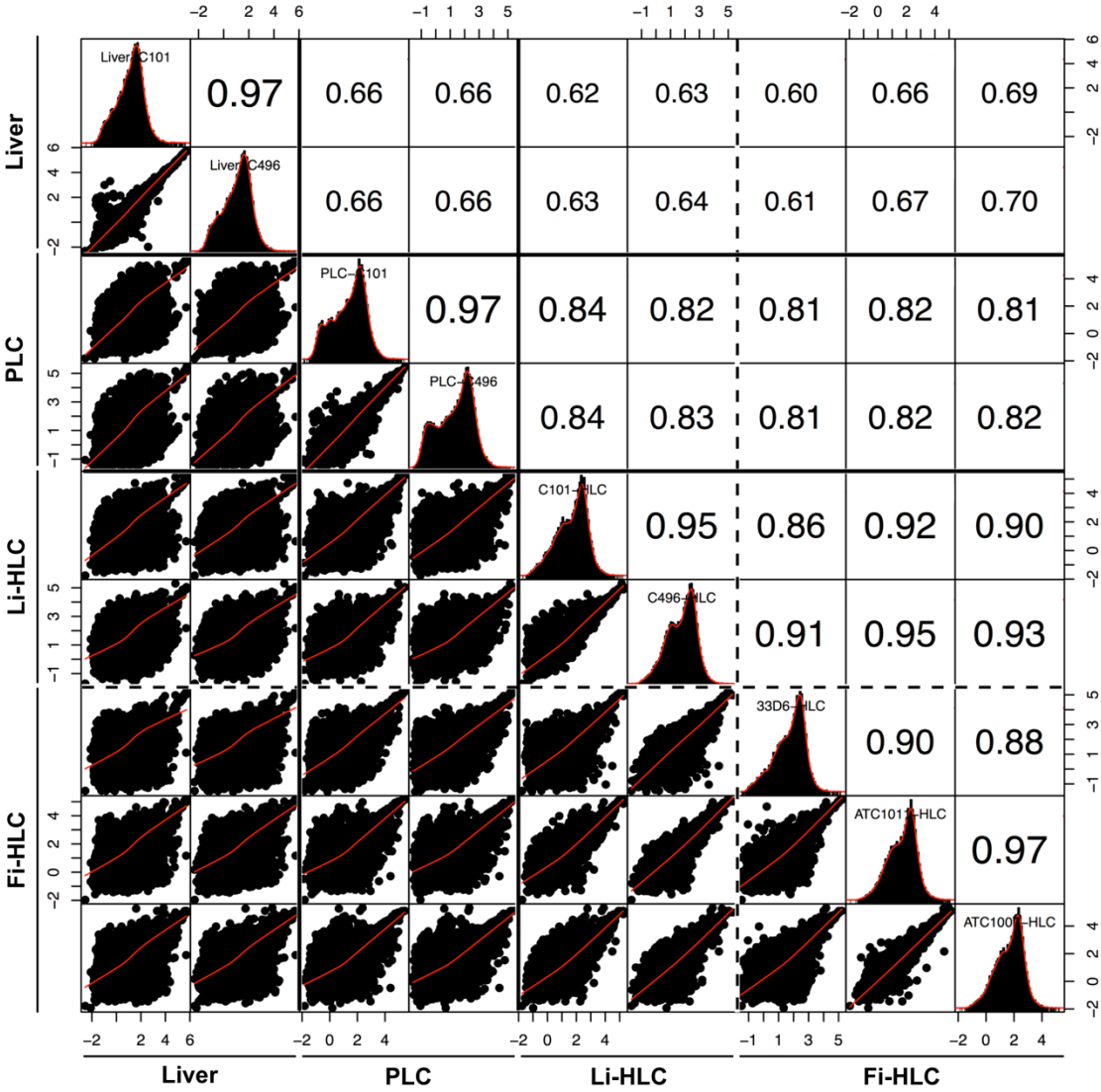

Supplement: S6 Fig — Pairwise correlation analysis (Spearman’s ranks correlation) was performed between each of 9 transcriptome data sets derived from two originating liver biopsy samples, two derived PLC samples, two Li-HLC samples (C101-HLC and C496-HLC) and 3 Fi-HLC samples. The gene expression frequency distribution for each sample is shown as histograms together with the sample name in the panels along the diagonal from the top left to bottom right of the figure. Gene expression correlations plots and Spearman’s correlation coefficient ρ for all sample pairs are shown to the left and right of the frequency distribution histograms, respectively. All correlations were statistically significant with a p-value ≤ 0.001. X- and Y-axes indicate scaled gene expression ((Log10TPM)+1). (PDF) [file pone.0221762.s006.pdf]

S7 Fig.

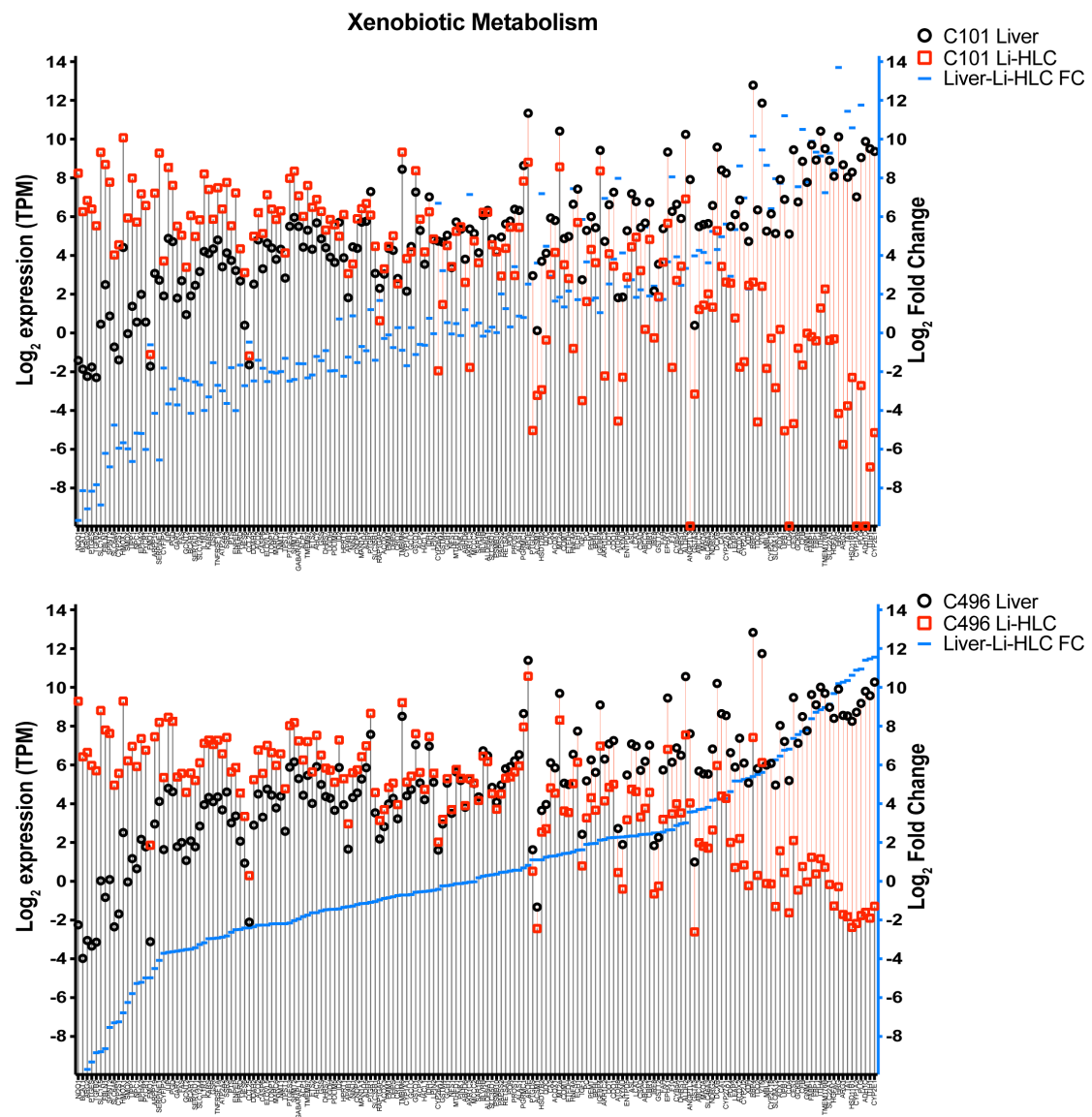

Supplement: S7 Fig — Expression levels (log2) of all the genes in the gene in the Li-HLCs (red squares) and liver (black circles) in patients C101 (top panel) and C496 (bottom panel). The horizontal lines reflect the log2-fold change of expression level between the liver and the Li-HLCs. Expression levels are indicated as transcripts per million (TPM). (PDF) [file pone.0221762.s007.pdf]
