# Supplementary material for: Liver biopsy derived induced pluripotent stem cells provide unlimited supply for the generation of hepatocyte-like cells
Source: PLoS One. 2019 Aug 29;14(8):e0221762. doi: 10.1371/journal.pone.0221762 (PMC6715171; doi:10.1371/journal.pone.0221762)
Supplement: S1 Table — (PDF) [file pone.0221762.s008.pdf]

S1 Table. Patient liver primary cell expansion and iPSC reprogramming

Biopsy ID	Disease	Age at biopsy	hPLCs expansion	Colony formation (# colonies)	Reprogramming efficiency	iPSC maintenance	Reprogramming xenogene free	Note
C086a	Alcoholic liver disease (ALD)	79	+	- (0)	NA	-	-	No emerging colonies
C091	Autoimmune hepatitis (AIH) in remission	66	+	+ (1)	0.001%	-	-	iPSC colony lost before passage 1
C101	Non-alcoholic liver disease (NAFLD)	76	+	+ (65)	0.040%	+	Yes	Ready to use after 20 passages
C496	Alcoholic liver disease (ALD)	32	+	+ (32)	0.019%	+	Yes	Ready to use after 20 passages
C824	Alcoholic liver disease (ALD)	45	+	- (0)	NA	-	-	No emerging colonies
C876	Normal liver	37	+	- (0)	NA	-	-	No emerging colonies
C877	Alcoholic liver disease (ALD)	60	+	+ (24)	0.016%	+	No	Reprogramming vectors detectable
C885	Alcoholic liver disease (ALD)	51	+	- (0)	NA	-	-	No emerging colonies
