# Supplementary material for: Liver biopsy derived induced pluripotent stem cells provide unlimited supply for the generation of hepatocyte-like cells
Source: PLoS One. 2019 Aug 29;14(8):e0221762. doi: 10.1371/journal.pone.0221762 (PMC6715171; doi:10.1371/journal.pone.0221762)
Supplement: S2 Table — (PDF) [file pone.0221762.s009.pdf]

S2 Table. q-PCR primer list

Target	Forward primer	Reverse primer	Product
SeV	GGA TCA CTA GGT GAT ATC GAG C	ACC AGA CAA GAG TTT AAG AGA TAT GTA TC	181 bp
SeV-KOS	ATG CAC CGC TAC GAC GTG AGC GC	ACC TTG ACA ATC CTG ATG TGG	528 bp
SeV-Klf4	TTC CTG CAT GCC AGA GGA GCC C	AAT GTA TCG AAG GTG CTC AA	410 bp
SeV-c-Myc	TAA CTG ACT AGC AGG CTT GTC G	TCC ACA TAC AGT CCT GGA TGA TGA TG	532 bp
