# Supplementary material for: Liver biopsy derived induced pluripotent stem cells provide unlimited supply for the generation of hepatocyte-like cells
Source: PLoS One. 2019 Aug 29;14(8):e0221762. doi: 10.1371/journal.pone.0221762 (PMC6715171; doi:10.1371/journal.pone.0221762)
Supplement: S3 Table — (PDF) [file pone.0221762.s010.pdf]

S3 Table. Gene sets significantly enriched in PLCs vs. Liver

Gene Set Name	# Genes[a]	% of Gene set[b]	p-value	FDR q-value
**EPITHELIAL TO MESENCHYMAL TRANSITION**	137	69%	8.79E-118	4.40E-116
G2M CHECKPOINT	100	50%	5.90E-67	1.47E-65
E2F TARGETS	84	42%	8.93E-49	1.49E-47
MITOTIC SPINDLE	82	41%	1.16E-46	1.44E-45
UV RESPONSE DOWN	65	45%	2.00E-40	2.00E-39
APICAL JUNCTION	72	36%	1.18E-36	9.83E-36
HYPOXIA	71	36%	1.05E-35	7.50E-35
TNFA SIGNALING VIA NFKB	67	34%	5.28E-32	3.30E-31
GLYCOLYSIS	65	33%	3.27E-30	1.82E-29
MYOGENESIS	64	32%	2.49E-29	1.24E-28

a. Number of significant genes overlapping with the Molecular Signatures Database (MSigDB) hallmark gene sets
b. Percentage of the hallmark gene set represented by the genes in overlap
