# Supplementary material for: Liver biopsy derived induced pluripotent stem cells provide unlimited supply for the generation of hepatocyte-like cells
Source: PLoS One. 2019 Aug 29;14(8):e0221762. doi: 10.1371/journal.pone.0221762 (PMC6715171; doi:10.1371/journal.pone.0221762)
Supplement: S4 Table — (PDF) [file pone.0221762.s011.pdf]

S4 Table. Summary of samples included in the RNAseq experiment for transcriptomic profiling (numbers indicate the number of replicates processed).

	**Source**				
	**Patient**		**Sullivan G.J. *et al.* [a]**	**ATCC[b]**	
	**C101**	**C496**	**33D6**	**ACS-1011**	**ACS-1007**
Liver	1	1			
hPLC	1	1			
Li-iPSC[c-d]	2	2			
Li-HLC[e]	2	2			
Fi-iPSC[c-f]			2	2	2
Fi-HLC[e]			2	2	2

a. 33D6 foreskin fibroblast derived iPSCs. Sullivan GJ, Hay DC, Park IH, Fletcher J, Hannoun Z, Payne CM, Dalgetty D, Black JR, Ross JA, Samuel K *et al*: **Generation of functional human hepatic endoderm from human induced pluripotent stem cells.** *Hepatology (Baltimore, Md)* 2010, **51**(1):329-335.
b. Commercial validated fibroblast derived iPSC lines acquired from ATCC, Manassas, Virginia, US.
c. iPSC culture replicates.
d. Sendai virus reprogrammed iPSCs.
e. HLCs differentiated from independent iPSC cultures (**c**.).
f. Retro virus reprogrammed iPSCs.
