# Supplementary material for: Liver biopsy derived induced pluripotent stem cells provide unlimited supply for the generation of hepatocyte-like cells
Source: PLoS One. 2019 Aug 29;14(8):e0221762. doi: 10.1371/journal.pone.0221762 (PMC6715171; doi:10.1371/journal.pone.0221762)
Supplement: S5 Table — (PDF) [file pone.0221762.s012.pdf]

S5 Table. Gene sets significantly enriched in Li-iPSCs vs. Fi-iPSCs

Gene Set Name	# Genes[a]	% of Gene set[b]	p-value	FDR q-value
EPITHELIAL MESENCHYMAL TRANSITION	23	12%	1.85E-16	9.27E-15
**COAGULATION**	17	12%	5.17E-13	1.29E-11
MYOGENESIS	19	10%	2.49E-12	4.15E-11
**XENOBIOTIC METABOLISM**	15	8%	1.31E-08	1.64E-07
APOPTOSIS	12	7%	3.97E-07	3.97E-06
APICAL JUNCTION	13	7%	6.32E-07	4.52E-06
ESTROGEN RESPONSE LATE	13	7%	6.32E-07	4.52E-06
INFLAMMATORY RESPONSE	12	6%	3.90E-06	1.95E-05
INTERFERON GAMMA RESPONSE	12	6%	3.90E-06	1.95E-05
KRAS SIGNALING UP	12	6%	3.90E-06	1.95E-05
ALLOGRAFT REJECTION	11	6%	2.22E-05	9.23E-05
**COMPLEMENT**	11	6%	2.22E-05	9.23E-05
**BILE ACID METABOLISM**	8	7%	4.69E-05	1.80E-04
**GLYCOLYSIS**	10	5%	1.15E-04	3.83E-04
TNFA SIGNALING VIA NFKB	10	5%	1.15E-04	3.83E-04
INTERFERON ALPHA RESPONSE	7	7%	1.31E-04	4.09E-04
HYPOXIA	9	5%	5.39E-04	1.59E-03
ANGIOGENESIS	4	11%	7.48E-04	2.08E-03
PEROXISOME	5	5%	7.01E-03	1.85E-02
IL6 JAK STAT3 SIGNALING	4	5%	1.79E-02	4.48E-02

a. Number of significant genes overlapping with the Molecular Signatures Database (MSigDB) hallmark gene sets
b. Percentage of the hallmark gene set represented by the genes in overlap
