# Supplementary material for: Liver biopsy derived induced pluripotent stem cells provide unlimited supply for the generation of hepatocyte-like cells
Source: PLoS One. 2019 Aug 29;14(8):e0221762. doi: 10.1371/journal.pone.0221762 (PMC6715171; doi:10.1371/journal.pone.0221762)
Supplement: S6 Table — (PDF) [file pone.0221762.s013.pdf]

S6 Table. Gene sets significantly down-regulated in Li-iPSCs vs. originating livers.

Gene Set Name	# Genes[a]	% of Gene set[b]	p-value	FDR q-value
**XENOBIOTIC METABOLISM**	115	58%	2.09E-90	1.05E-88
**FATTY ACID METABOLISM**	77	49%	1.36E-53	3.40E-52
INTERFERON GAMMA RESPONSE	83	42%	1.05E-50	1.75E-49
**COAGULATION**	67	49%	1.16E-46	1.44E-45
**BILE ACID METABOLISM**	61	54%	1.56E-46	1.56E-45
**ADIPOGENESIS**	72	36%	3.51E-39	2.92E-38
INTERFERON ALPHA RESPONSE	45	46%	1.06E-30	7.54E-30
**COMPLEMENT**	61	31%	8.20E-29	5.12E-28
KRAS SIGNALING UP	59	30%	4.66E-27	2.59E-26
PEROXISOME	43	41%	7.36E-27	3.68E-26
ESTROGEN RESPONSE EARLY	55	28%	1.12E-23	5.10E-23
HYPOXIA	54	27%	7.38E-23	3.08E-22
ESTROGEN RESPONSE LATE	53	27%	4.74E-22	1.82E-21
TNFA SIGNALING VIA NFKB	52	26%	2.96E-21	1.06E-20
APOPTOSIS	46	29%	8.69E-21	2.90E-20
**HEME METABOLISM**	51	26%	1.81E-20	5.65E-20
IL2 STAT5 SIGNALING	49	25%	6.20E-19	1.82E-18
EPITHELIAL MESENCHYMAL TRANSITION	47	24%	1.91E-17	5.31E-17
**GLYCOLYSIS**	46	23%	1.02E-16	2.54E-16
INFLAMMATORY RESPONSE	46	23%	1.02E-16	2.54E-16
UV RESPONSE UP	39	25%	1.73E-15	4.11E-15
OXIDATIVE PHOSPHORYLATION	42	21%	6.15E-14	1.34E-13
P53 PATHWAY	42	21%	6.15E-14	1.34E-13
UV RESPONSE DN	34	24%	4.31E-13	8.97E-13
**CHOLESTEROL HOMEOSTASIS**	24	32%	7.11E-13	1.42E-12
MYOGENESIS	39	20%	5.49E-12	1.06E-11

ALLOGRAFT REJECTION	38	19%	2.31E-11	4.27E-11
IL6 JAK STAT3 SIGNALING	24	28%	3.45E-11	6.16E-11
REACTIVE OXIGEN SPECIES PATHWAY	15	31%	3.48E-08	6.00E-08
ANDROGEN RESPONSE	20	20%	5.87E-07	9.78E-07
APICAL JUNCTION	30	15%	6.70E-07	1.08E-06
KRAS SIGNALING DN	27	14%	1.73E-05	2.68E-05
ANGIOGENESIS	10	28%	1.77E-05	2.68E-05
MTORC1 SIGNALING	25	13%	1.24E-04	1.82E-04
PANCREAS BETA CELLS	8	20%	1.36E-03	1.94E-03
DNA REPAIR	15	10%	1.94E-02	2.69E-02
UNFOLDED PROTEIN RESPONSE	12	11%	2.25E-02	3.05E-02
TGF BETA SIGNALING	7	13%	2.85E-02	3.75E-02
NOTCH SIGNALING	5	16%	2.98E-02	3.82E-02

a. Number of significant genes overlapping with the Molecular Signatures Database (MSigDB) hallmark gene sets

b. Percentage of the hallmark gene set represented by the genes in overlap
