# Supplementary material for: Liver biopsy derived induced pluripotent stem cells provide unlimited supply for the generation of hepatocyte-like cells
Source: PLoS One. 2019 Aug 29;14(8):e0221762. doi: 10.1371/journal.pone.0221762 (PMC6715171; doi:10.1371/journal.pone.0221762)
Supplement: S7 Table — (PDF) [file pone.0221762.s014.pdf]

S7 Table. liver-specific gene sets (Molecular Signatures Database) expression in Li-HLCs compared to parental livers.

Ensembl Transcript ID	Gene Symbol	C101					C496				
		Liver	Li-HLC	Li-HLC/Liver[a]	p-value	Li-HLC/Li-iPSC[b]	Liver	Li-HLC	Li-HLC/Liver[a]	p-value	Li-HLC/Li-iPSC[b]
**Hallmark Xenobiotic Metabolism**											
ENSG00000023839	**ABCC2**	50.99	1.14	0.02	1.56E-07	1.4	73.08	13.59	0.19	1.00E-04	24.7
ENSG00000108846	**ABCC3**	44.93	2.33	0.05	8.83E-07	39.1	51.69	3.97	0.08	3.45E-06	26.3
ENSG00000163686	**ABHD6**	14.00	6.10	0.44	0.00E+00	1.9	14.14	14.98	1.06	2.20E-03	5.0
ENSG00000100412	**ACO2**	27.97	74.05	2.65	8.80E-03	1.2	22.70	108.94	4.80	NS	1.6
ENSG00000161533	**ACOX1**	55.87	17.86	0.32	7.51E-07	1.9	57.78	23.32	0.40	2.60E-06	2.8
ENSG00000168306	**ACOX2**	116.27	0.29	0.00	1.50E-08	0.1	166.28	4.61	0.03	3.85E-06	1.6
ENSG00000087008	**ACOX3**	8.23	9.97	1.21	1.00E-04	1.5	7.06	13.00	1.84	8.00E-04	2.0
ENSG00000143727	**ACP1**	21.52	64.73	3.01	NS	0.7	21.69	76.61	3.53	NS	0.8
ENSG00000134575	**ACP2**	39.18	58.75	1.50	9.00E-04	3.6	33.60	48.93	1.46	8.00E-04	2.7
ENSG00000166743	**ACSM1**	1.09	0.11	0.10	2.00E-04	1.2	0.40	0.18	0.46	7.00E-04	0.5
ENSG00000248144	**ADH1C**	944.48	0.00	0.00	1.11E-10	#DIV/0!	892.47	0.33	0.00	3.44E-09	2.3
ENSG00000197894	**ADH5**	54.27	102.56	1.89	9.00E-04	0.8	57.95	127.08	2.19	3.00E-03	0.9
ENSG00000101444	**AHCY**	51.43	119.04	2.31	2.40E-03	0.4	60.39	184.61	3.06	NS	0.5
ENSG00000151632	**AKR1C2**	26.54	0.22	0.01	8.37E-07	0.9	78.90	17.72	0.22	4.00E-04	29.5
ENSG00000196139	**AKR1C3**	41.27	0.29	0.01	5.06E-08	0.3	38.08	39.21	1.03	NS	14.8
ENSG00000023330	**ALAS1**	80.30	43.64	0.54	0.00E+00	1.0	92.31	61.93	0.67	1.00E-04	1.5
ENSG00000111275	**ALDH2**	1213.55	120.45	0.10	1.65E-07	2.0	1511.06	187.48	0.12	7.77E-07	3.2
ENSG00000143149	**ALDH9A1**	80.16	75.13	0.94	0.00E+00	0.9	89.10	72.28	0.81	0.00E+00	0.8
ENSG00000132855	**ANGPTL3**	242.72	0.00	0.00	5.64E-09	#DIV/0!	195.47	16.45	0.08	7.00E-04	269.0
ENSG00000138356	**AOX1**	241.67	1.14	0.00	3.63E-07	0.2	262.38	2.98	0.01	1.76E-06	0.4
ENSG00000134262	**AP4B1**	3.54	8.37	2.36	NS	1.2	3.15	7.79	2.47	NS	1.1
ENSG00000130203	**APOE**	2589.24	449.37	0.17	0.00E+00	1.3	2690.60	1522.84	0.57	6.00E-04	4.1
ENSG00000118520	**ARG1**	410.57	0.02	0.00	3.15E-09	0.2	376.90	0.31	0.00	2.61E-09	1.1
ENSG00000081181	**ARG2**	0.21	114.22	538.65	4.13E-06	2.0	0.12	100.17	829.42	5.57E-06	1.5
ENSG00000128989	**ARPP19**	8.43	148.54	17.62	1.50E-03	1.2	7.77	175.45	22.59	1.90E-03	1.4
ENSG00000126522	**ASL**	145.69	21.73	0.15	5.71E-06	1.1	135.89	26.98	0.20	0.00E+00	1.1
ENSG00000168874	**ATOH8**	3.54	0.04	0.01	1.59E-06	0.2	6.62	1.37	0.21	1.30E-03	3.6
ENSG00000174437	**ATP2A2**	10.67	84.29	7.90	NS	1.0	12.85	94.95	7.39	NS	1.0

ENSG00000050820	**BCAR1**	3.78	66.83	17.68	3.00E-04	2.7	4.24	47.86	11.28	4.80E-03	2.0
ENSG00000060982	**BCAT1**	0.27	76.99	281.31	0.00E+00	0.6	0.06	85.84	1346.09	0.00E+00	0.5
ENSG00000090013	**BLVRB**	66.94	74.93	1.12	4.00E-04	2.9	106.09	87.90	0.83	7.00E-04	3.0
ENSG00000137274	**BPHL**	43.37	12.37	0.29	0.00E+00	0.9	49.15	12.70	0.26	0.00E+00	0.9
ENSG00000104267	**CA2**	61.14	8.06	0.13	2.07E-06	0.7	69.17	28.19	0.41	1.00E-04	3.4
ENSG00000138794	**CASP6**	9.96	35.10	3.52	NS	1.5	9.91	47.46	4.79	NS	1.6
ENSG00000121691	**CAT**	341.00	10.86	0.03	1.54E-07	0.9	400.58	21.17	0.05	5.49E-07	1.5
ENSG00000159228	**CBR1**	106.87	28.48	0.27	4.13E-06	0.3	130.30	24.05	0.18	2.25E-06	0.3
ENSG00000131142	**CCL25**	0.32	0.44	1.38	NS	1.1	0.23	1.23	5.29	NS	1.5
ENSG00000158825	**CDA**	34.51	0.00	0.00	1.04E-08	0.0	36.58	0.32	0.01	1.99E-06	0.0
ENSG00000129596	**CDO1**	153.87	10.95	0.07	7.80E-08	0.5	153.12	32.11	0.21	0.00E+00	1.5
ENSG00000198848	**CES1**	701.00	0.04	0.00	4.74E-10	0.3	713.75	4.30	0.01	9.05E-09	1.1
ENSG00000243649	**CFB**	463.52	0.32	0.00	7.08E-08	9.3	359.88	1.70	0.00	3.80E-07	11.7
ENSG00000133313	**CNDP2**	20.68	59.50	2.88	5.10E-03	1.2	23.53	53.88	2.29	5.10E-03	1.1
ENSG00000093010	**COMT**	29.29	11.49	0.39	0.00E+00	3.0	33.39	12.35	0.37	0.00E+00	2.0
ENSG00000005469	**CROT**	7.03	5.81	0.83	1.00E-04	1.8	9.36	15.57	1.66	5.00E-03	5.3
ENSG00000139631	**CSAD**	43.59	9.29	0.21	1.00E-04	3.4	52.84	9.93	0.19	2.00E-04	3.2
ENSG00000166347	**CYB5A**	99.96	6.56	0.07	1.59E-07	0.4	117.69	16.00	0.14	3.65E-06	0.9
ENSG00000055163	**CYFIP2**	3.76	13.14	3.49	NS	0.5	3.12	40.83	13.10	NS	1.6
ENSG00000140465	**CYP1A1**	4.46	14.33	3.21	NS	25.6	21.25	34.58	1.63	NS	42.8
ENSG00000140505	**CYP1A2**	130.03	0.00	0.00	9.11E-08	0.0	421.27	0.22	0.00	3.28E-07	1.5
ENSG00000095596	**CYP26A1**	26.85	0.26	0.01	1.00E-04	0.1	3.05	4.05	1.33	7.10E-03	0.5
ENSG00000135929	**CYP27A1**	303.83	6.18	0.02	2.50E-08	1.0	374.56	19.40	0.05	8.89E-07	4.8
ENSG00000108242	**CYP2C18**	70.80	0.83	0.01	1.21E-07	5.4	67.77	0.91	0.01	3.77E-07	2.9
ENSG00000130649	**CYP2E1**	661.76	0.03	0.00	3.87E-09	2.0	1238.74	0.41	0.00	1.73E-08	1.9
ENSG00000134716	**CYP2J2**	44.90	0.36	0.01	3.03E-08	0.3	68.68	1.80	0.03	1.20E-06	0.9
ENSG00000167600	**CYP2S1**	0.38	23.44	61.32	4.20E-03	0.1	0.31	47.37	151.80	1.20E-03	0.2
ENSG00000169738	**DCXR**	770.67	38.77	0.05	8.46E-06	1.0	1180.46	62.79	0.05	0.00E+00	1.6
ENSG00000213722	**DDAH2**	5.73	31.78	5.54	NS	1.9	7.46	37.94	5.08	NS	1.7
ENSG00000132437	**DDC**	17.34	0.78	0.04	4.10E-07	7.9	15.58	6.56	0.42	0.00E+00	44.5
ENSG00000099977	**DDT**	98.30	17.01	0.17	4.12E-06	0.7	135.98	28.72	0.21	0.00E+00	1.2
ENSG00000095059	**DHPS**	19.24	32.46	1.69	4.10E-03	1.0	19.44	33.57	1.73	5.20E-03	0.9
ENSG00000157379	**DHRS1**	59.95	10.86	0.18	1.00E-04	2.1	90.41	11.52	0.13	1.00E-04	1.7

ENSG00000100612	**DHRS7**	21.15	39.71	1.88	1.00E-03	2.9	20.77	57.34	2.76	8.50E-03	2.7
ENSG00000104823	**ECH1**	172.35	52.06	0.30	4.18E-06	0.7	215.23	70.43	0.33	0.00E+00	0.8
ENSG00000012660	**ELOVL5**	24.80	140.67	5.67	NS	1.0	27.04	128.98	4.77	NS	0.9
ENSG00000138792	**ENPEP**	9.37	149.95	16.01	NS	229.8	10.35	58.28	5.63	NS	83.3
ENSG00000187097	**ENTPD5**	38.81	7.41	0.19	6.09E-06	3.0	44.70	9.00	0.20	8.88E-06	2.2
ENSG00000142627	**EPHA2**	3.98	144.97	36.47	8.09E-06	4.4	4.47	164.93	36.87	0.00E+00	6.0
ENSG00000143819	**EPHX1**	646.92	50.60	0.08	6.73E-08	0.9	700.07	111.99	0.16	4.84E-06	1.9
ENSG00000171503	**ETFDH**	31.63	7.04	0.22	0.00E+00	1.1	31.97	11.69	0.37	0.00E+00	1.6
ENSG00000157557	**ETS2**	19.99	89.75	4.49	NS	2.7	16.21	49.83	3.07	NS	1.9
ENSG00000126218	**F10**	77.41	0.29	0.00	3.90E-08	0.4	70.46	11.16	0.16	0.00E+00	4.3
ENSG00000163586	**FABP1**	833.54	0.87	0.00	3.11E-08	32.2	792.69	2.37	0.00	2.21E-06	16.0
ENSG00000103876	**FAH**	41.82	12.60	0.30	1.83E-06	2.3	53.48	9.19	0.17	0.00E+00	1.5
ENSG00000026103	**FAS**	6.49	32.96	5.07	NS	3.3	4.01	47.32	11.80	NS	9.9
ENSG00000077942	**FBLN1**	5.62	413.78	73.61	5.00E-04	6.6	0.56	223.88	396.42	3.00E-03	3.6
ENSG00000165140	**FBP1**	486.35	0.76	0.00	7.12E-09	0.2	549.27	1.30	0.00	1.53E-08	0.4
ENSG00000010932	**FMO1**	0.31	0.47	1.53	NS	25.9	0.12	3.63	31.37	4.10E-03	97.7
ENSG00000139112	**GABARAPL 1**	45.19	134.94	2.99	NS	2.4	39.32	151.39	3.85	NS	3.1
ENSG00000159131	**GART**	3.49	45.64	13.07	NS	0.6	3.45	41.67	12.09	NS	0.6
ENSG00000131979	**GCH1**	32.99	22.78	0.69	0.00E+00	2.1	33.57	39.22	1.17	9.00E-04	2.6
ENSG00000084734	**GCKR**	108.84	0.58	0.01	3.55E-07	2.6	139.02	0.73	0.01	2.39E-06	2.2
ENSG00000001084	**GCLC**	22.08	18.19	0.82	0.00E+00	0.8	26.65	42.87	1.61	1.40E-03	1.8
ENSG00000111846	**GCNT2**	1.93	10.50	5.45	NS	0.4	2.10	24.03	11.42	NS	1.1
ENSG00000124713	**GNMT**	219.19	0.99	0.00	3.64E-08	0.4	218.39	0.98	0.00	8.88E-08	0.4
ENSG00000104687	**GSR**	20.26	58.68	2.90	NS	0.7	17.26	133.39	7.73	NS	1.7
ENSG00000100983	**GSS**	28.99	77.48	2.67	NS	2.0	31.72	91.31	2.88	NS	2.1
ENSG00000174156	**GSTA3**	1.31	0.11	0.09	2.53E-06	#DIV/0!	1.99	0.16	0.08	0.00E+00	#DIV/0!
ENSG00000168765	**GSTM4**	25.82	2.77	0.11	5.00E-04	0.4	7.78	9.16	1.18	5.60E-03	1.3
ENSG00000148834	**GSTO1**	154.17	332.41	2.16	7.20E-03	8.3	132.14	195.18	1.48	4.00E-04	8.7
ENSG00000099984	**GSTT2**	11.74	3.65	0.31	NS	0.4	4.79	0.85	0.18	8.00E-04	0.2
ENSG00000131373	**HACL1**	11.69	18.16	1.55	7.00E-04	0.5	18.49	26.67	1.44	2.90E-03	0.5
ENSG00000144485	**HES6**	1.32	8.65	6.56	NS	0.4	1.91	10.17	5.31	NS	0.4
ENSG00000109758	**HGFAC**	273.15	0.81	0.00	3.33E-06	6.9	339.75	0.41	0.00	1.21E-06	2.5
ENSG00000100292	**HMOX1**	21.36	1079.95	50.56	9.30E-03	33.2	5.71	628.24	110.09	NS	15.6

ENSG00000101076	**HNF4A**	100.42	0.57	0.01	3.22E-08	92.0	93.59	32.79	0.35	4.00E-04	553.1
ENSG00000165704	**HPRT1**	14.75	69.16	4.69	NS	0.9	15.46	39.13	2.53	NS	0.5
ENSG00000113905	**HRG**	1116.51	0.06	0.00	3.54E-09	0.7	965.07	0.82	0.00	1.09E-07	2.9
ENSG00000117594	**HSD11B1**	315.05	0.20	0.00	1.14E-08	#DIV/0!	305.94	0.19	0.00	1.94E-07	3.0
ENSG00000086696	**HSD17B2**	13.01	0.13	0.01	1.40E-07	#DIV/0!	12.59	5.82	0.46	5.00E-04	1642.7
ENSG00000115738	**ID2**	52.26	31.75	0.61	0.00E+00	1.0	58.39	156.29	2.68	NS	6.2
ENSG00000138413	**IDH1**	130.12	76.64	0.59	8.11E-06	0.3	125.10	176.61	1.41	1.20E-03	0.5
ENSG00000017427	**IGF1**	6.70	0.09	0.01	0.00E+00	11.2	5.37	1.74	0.32	7.00E-04	142.5
ENSG00000141753	**IGFBP4**	686.62	330.07	0.48	0.00E+00	2.5	546.56	125.63	0.23	1.00E-04	0.7
ENSG00000115594	**IL1R1**	18.48	295.17	15.97	2.00E-03	847.9	15.47	139.16	8.99	NS	371.3
ENSG00000140968	**IRF8**	4.51	0.84	0.19	9.66E-07	2.9	3.59	0.64	0.18	1.47E-06	2.3
ENSG00000055957	**ITIH1**	727.41	0.01	0.00	1.29E-09	#DIV/0!	764.58	0.27	0.00	3.43E-09	1.3
ENSG00000055955	**ITIH4**	1366.41	2.44	0.00	1.31E-09	4.6	1034.48	2.24	0.00	3.85E-08	2.5
ENSG00000173801	**JUP**	29.44	372.69	12.66	2.00E-04	5.9	27.78	351.69	12.66	6.40E-03	4.6
ENSG00000065427	**KARS**	17.20	169.37	9.85	5.20E-03	1.0	20.03	155.79	7.78	NS	0.9
ENSG00000115919	**KYNU**	3.61	0.21	0.06	6.13E-07	5.7	3.72	0.76	0.20	0.00E+00	23.9
ENSG00000213398	**LCAT**	110.40	30.81	0.28	1.88E-06	6.0	124.45	24.67	0.20	2.10E-06	5.5
ENSG00000164406	**LEAP2**	69.33	1.71	0.02	2.45E-07	1.0	59.29	1.64	0.03	3.33E-07	0.8
ENSG00000196365	**LONP1**	20.91	84.25	4.03	NS	0.9	21.76	99.42	4.57	NS	1.2
ENSG00000101577	**LPIN2**	28.14	28.91	1.03	1.00E-04	2.2	34.43	47.49	1.38	1.40E-03	3.4
ENSG00000111885	**MAN1A1**	52.89	85.74	1.62	3.30E-03	23.3	38.84	85.94	2.21	6.60E-03	25.1
ENSG00000189221	**MAOA**	48.58	2.71	0.06	5.37E-07	0.6	46.58	3.50	0.08	9.56E-07	0.7
ENSG00000145495	**MARC6**	13.92	58.16	4.18	NS	1.7	13.80	63.01	4.57	NS	1.7
ENSG00000165471	**MBL2**	38.25	0.28	0.01	7.57E-06	#DIV/0!	63.80	0.93	0.01	0.00E+00	50.1
ENSG00000131844	**MCCC2**	35.05	26.96	0.77	0.00E+00	0.4	32.98	33.43	1.01	1.00E-04	0.5
ENSG00000125148	**MT2A**	1369.60	379.63	0.28	2.00E-04	5.4	827.35	319.32	0.39	1.00E-04	2.6
ENSG00000100714	**MTHFD1**	53.10	37.93	0.71	1.00E-04	0.3	50.04	54.53	1.09	3.00E-04	0.4
ENSG00000165795	**NDRG2**	95.59	2.52	0.03	7.08E-08	0.4	113.32	6.23	0.06	3.92E-07	0.8
ENSG00000244005	**NFS1**	10.38	10.76	1.04	1.00E-04	1.1	11.37	12.97	1.14	3.00E-04	1.3
ENSG00000131669	**NINJ1**	21.81	11.80	0.54	2.39E-06	0.7	19.98	48.42	2.42	NS	2.5
ENSG00000136448	**NMT1**	19.97	78.89	3.95	NS	1.1	20.90	95.20	4.55	NS	1.2
ENSG00000141458	**NPC1**	1.47	52.94	35.91	7.00E-04	3.2	1.57	60.55	38.59	7.00E-04	3.4
ENSG00000181019	**NQO1**	0.38	303.69	809.49	1.00E-06	10.1	0.21	626.55	2962.00	1.18E-06	13.9

ENSG00000198682	**PAPSS2**	30.97	7.67	0.25	4.63E-06	0.3	31.27	22.68	0.73	2.00E-04	0.8
ENSG00000173599	**PC**	39.27	3.08	0.08	6.02E-07	0.4	36.26	9.69	0.27	8.61E-06	1.1
ENSG00000004799	**PDK4**	45.07	5.94	0.13	8.07E-06	73.5	99.37	4.03	0.04	0.00E+00	49.8
ENSG00000163110	**PDLIM5**	12.51	48.05	3.84	NS	2.2	12.67	34.45	2.72	NS	2.5
ENSG00000133027	**PEMT**	64.28	19.85	0.31	1.14E-06	0.6	76.71	19.92	0.26	1.94E-06	0.6
ENSG00000142657	**PGD**	26.29	196.36	7.47	6.70E-03	0.7	24.61	304.70	12.38	1.40E-03	0.9
ENSG00000101856	**PGRMC1**	398.83	228.67	0.57	2.73E-06	0.7	403.13	249.17	0.62	7.57E-06	0.7
ENSG00000158828	**PINK1**	40.96	44.73	1.09	0.00E+00	3.1	36.75	39.73	1.08	1.00E-04	3.3
ENSG00000122194	**PLG**	531.41	0.15	0.00	6.04E-09	7.3	580.42	0.29	0.00	2.06E-08	1.7
ENSG00000100417	**PMM1**	20.61	22.13	1.07	NS	6.2	15.91	28.61	1.80	NS	7.1
ENSG00000127948	**POR**	54.92	44.06	0.80	0.00E+00	1.2	60.24	41.83	0.69	0.00E+00	1.1
ENSG00000112033	**PPARD**	6.45	20.34	3.15	NS	1.3	4.20	23.44	5.58	NS	1.5
ENSG00000184500	**PROS1**	83.88	7.80	0.09	2.25E-06	6.1	74.24	49.81	0.67	1.00E-04	25.2
ENSG00000205220	**PSMB10**	18.54	18.29	0.99	2.00E-04	1.1	17.00	13.16	0.77	1.20E-03	0.8
ENSG00000107317	**PTGDS**	7.76	0.03	0.00	5.53E-06	0.2	3.10	1.43	0.46	1.30E-03	5.7
ENSG00000148344	**PTGES**	0.30	84.66	285.84	0.00E+00	20.0	0.10	63.02	636.52	2.00E-04	10.6
ENSG00000110958	**PTGES3**	45.43	252.13	5.55	NS	0.7	59.17	261.65	4.42	NS	0.6
ENSG00000106853	**PTGR1**	156.93	68.07	0.43	3.00E-04	4.2	191.26	404.26	2.11	NS	19.1
ENSG00000150787	**PTS**	13.44	46.23	3.44	NS	4.2	8.06	49.80	6.18	NS	4.8
ENSG00000183010	**PYCR1**	1.48	95.39	64.43	1.20E-03	0.8	3.44	108.82	31.63	1.10E-03	0.9
ENSG00000076864	**RAP1GAP**	4.96	1.55	0.31	0.00E+00	0.3	4.55	8.84	1.94	NS	1.4
ENSG00000138207	**RBP4**	7050.33	6.18	0.00	8.54E-08	8.9	7284.92	171.30	0.02	1.07E-06	121.3
ENSG00000042445	**RETSAT**	49.45	20.76	0.42	4.92E-06	1.8	55.79	39.89	0.71	1.00E-04	3.1
ENSG00000152700	**SAR1B**	17.66	12.34	0.70	6.20E-06	2.4	20.48	18.01	0.88	1.00E-04	3.4
ENSG00000106366	**SERPINE1**	6.63	623.82	94.12	2.00E-04	70.5	17.41	294.22	16.90	3.80E-03	30.8
ENSG00000197019	**SERTAD1**	5.51	31.68	5.75	NS	4.6	3.44	36.91	10.72	NS	3.9
ENSG00000182199	**SHMT2**	62.21	326.69	5.25	NS	2.4	71.70	290.96	4.06	NS	2.0
ENSG00000124067	**SLC12A4**	8.99	57.37	6.38	NS	4.8	7.19	69.15	9.61	NS	6.1
ENSG00000105281	**SLC1A5**	1.37	640.33	467.30	4.81E-06	3.2	1.02	449.17	440.21	0.00E+00	1.8
ENSG00000175003	**SLC22A1**	480.10	0.77	0.00	1.57E-09	3.0	501.68	0.90	0.00	2.10E-09	2.3
ENSG00000121073	**SLC35B1**	8.41	22.32	2.65	NS	1.2	11.58	23.93	2.07	NS	1.2
ENSG00000116704	**SLC35D1**	29.06	23.47	0.81	2.00E-04	6.3	28.84	22.87	0.79	2.00E-04	5.2
ENSG00000139508	**SLC46A3**	50.08	4.05	0.08	8.81E-08	10.4	46.22	3.28	0.07	9.46E-08	7.5

ENSG00000111181	**SLC6A12**	35.18	0.14	0.00	2.52E-09	0.6	31.24	0.41	0.01	9.35E-08	1.5
ENSG00000131389	**SLC6A6**	0.61	16.28	26.85	NS	0.2	0.20	30.98	157.19	9.10E-03	0.3
ENSG00000088826	**SMOX**	0.98	61.28	62.66	6.63E-06	7.0	0.98	74.16	75.90	0.00E+00	8.6
ENSG00000167642	**SPINT2**	1.84	220.06	119.54	1.36E-06	2.4	1.06	197.31	185.59	3.01E-06	2.0
ENSG00000114850	**SSR3**	17.58	218.13	12.41	9.90E-03	2.1	24.38	171.04	7.02	NS	1.5
ENSG00000198650	**TAT**	262.82	0.07	0.00	6.55E-08	1.1	369.98	0.28	0.00	1.28E-07	2.4
ENSG00000151790	**TDO2**	81.84	0.04	0.00	1.35E-06	3.4	56.20	1.23	0.02	5.33E-07	24.6
ENSG00000092969	**TGFB2**	0.20	46.28	227.08	2.22E-06	58.5	0.11	52.05	459.11	1.00E-04	126.6
ENSG00000139644	**TMBIM6**	349.10	644.92	1.85	7.00E-04	2.4	364.67	594.46	1.63	5.00E-04	2.1
ENSG00000106565	**TMEM176B**	729.37	4.81	0.01	8.81E-09	5.7	828.05	1.65	0.00	3.93E-07	1.8
ENSG00000109084	**TMEM97**	39.46	195.66	4.96	NS	1.5	44.19	148.19	3.35	NS	1.0
ENSG00000067182	**TNFRSF1A**	27.88	180.71	6.48	NS	6.3	20.46	154.69	7.56	NS	4.7
ENSG00000169902	**TPST1**	7.14	17.53	2.46	5.40E-03	1.4	6.00	27.33	4.55	NS	2.3
ENSG00000109814	**UGDH**	15.02	58.13	3.87	NS	1.0	19.49	53.10	2.72	NS	0.9
ENSG00000100024	**UPB1**	118.60	0.03	0.00	2.01E-07	0.4	148.34	1.37	0.01	0.00E+00	28.2
ENSG00000183696	**UPP1**	2.59	255.02	98.37	2.38E-06	14.1	2.26	124.64	55.25	1.10E-03	7.3
ENSG00000109072	**VTN**	3720.59	5.33	0.00	1.61E-08	23.5	3437.38	69.82	0.02	3.10E-08	91.4
ENSG00000158125	**XDH**	26.51	5.50	0.21	8.28E-06	5.5	33.67	0.86	0.03	0.00E+00	2.8

Hallmark Bile Acid Metabolism

ENSG00000165029	**ABCA1**	16.96	15.04	0.89	1.60E-03	1.5	10.22	88.06	8.62	NS	11.7
ENSG00000107331	**ABCA2**	10.88	1.14	0.10	3.72E-07	0.2	9.70	5.46	0.56	3.00E-04	0.9
ENSG00000167972	**ABCA3**	0.57	2.61	4.56	NS	1.5	0.35	8.58	24.33	NS	1.4
ENSG00000198691	**ABCA4**	0.24	2.46	10.06	NS	5.5	0.13	2.69	19.96	NS	23.0
ENSG00000154265	**ABCA5**	10.80	7.14	0.66	0.00E+00	7.3	13.18	5.60	0.42	1.00E-04	6.1
ENSG00000154262	**ABCA6**	14.15	0.08	0.01	5.68E-07	0.0	11.88	0.04	0.00	3.40E-07	3.0
ENSG00000141338	**ABCA8**	12.34	0.06	0.00	0.00E+00	0.0	13.25	0.19	0.01	8.73E-08	20.9
ENSG00000154258	**ABCA9**	11.45	0.22	0.02	1.07E-07	5.6	6.61	0.14	0.02	8.91E-07	1.7
ENSG00000101986	**ABCD1**	4.18	11.78	2.82	NS	1.9	3.65	9.76	2.68	NS	2.2
ENSG00000173208	**ABCD2**	0.06	0.00	0.00	4.00E-04	0.6	0.02	0.30	14.80	NS	11.2
ENSG00000117528	**ABCD3**	22.77	24.28	1.07	1.00E-04	1.6	32.27	47.05	1.46	2.10E-03	3.0
ENSG00000172350	**ABCG4**	0.04	0.06	1.40	NS	1.0	0.02	0.18	10.78	NS	0.2
ENSG00000143921	**ABCG8**	65.92	0.00	0.00	1.60E-09	0.0	52.70	0.02	0.00	1.81E-08	0.2
ENSG00000151726	**ACSL1**	256.41	56.13	0.22	0.00E+00	8.0	291.26	48.01	0.16	0.00E+00	4.7

ENSG00000197142	**ACSL5**	54.68	11.74	0.21	5.15E-07	#DIV/0!	65.06	4.67	0.07	4.00E-04	150.9
ENSG00000172482	**AGXT**	943.53	0.00	0.00	3.34E-10	0.0	952.18	0.36	0.00	2.82E-09	2.0
ENSG00000122787	**AKR1D1**	106.72	0.34	0.00	1.33E-06	2.4	150.69	6.09	0.04	3.04E-06	40.4
ENSG00000165092	**ALDH1A1**	346.80	1.14	0.00	5.24E-08	1.4	425.78	393.98	0.93	NS	290.7
ENSG00000118514	**ALDH8A1**	120.95	1.05	0.01	1.02E-08	5.7	117.98	0.88	0.01	2.59E-08	4.5
ENSG00000143149	**ALDH9A1**	80.16	75.13	0.94	0.00E+00	0.9	89.10	72.28	0.81	0.00E+00	0.8
ENSG00000242110	**AMACR**	44.64	10.09	0.23	1.61E-06	1.6	45.75	16.47	0.36	5.84E-06	2.3
ENSG00000118137	**APOA1**	11000.82	13.80	0.00	1.39E-07	21.7	8593.47	899.42	0.10	1.00E-04	429.3
ENSG00000103569	**AQP9**	294.35	0.00	0.00	3.18E-10	0.0	292.75	0.08	0.00	4.07E-09	1.3
ENSG00000169083	**AR**	15.33	0.43	0.03	4.30E-06	0.1	17.91	0.64	0.04	3.20E-06	0.1
ENSG00000124788	**ATXN1**	2.42	23.51	9.70	NS	8.1	2.56	11.88	4.65	NS	34.9
ENSG00000129151	**BBOX1**	37.01	0.08	0.00	1.69E-07	0.0	24.18	0.19	0.01	6.81E-07	4.6
ENSG00000137936	**BCAR3**	3.31	77.63	23.47	2.00E-04	11.6	3.16	34.39	10.87	NS	4.8
ENSG00000153162	**BMP6**	0.72	0.66	0.91	NS	0.9	0.20	103.67	531.46	NS	67.6
ENSG00000121691	**CAT**	341.00	10.86	0.03	1.54E-07	0.9	400.58	21.17	0.05	5.49E-07	1.5
ENSG00000138135	**CH25H**	0.19	0.00	0.00	4.00E-04	0.9	0.06	0.05	0.83	1.90E-03	0.1
ENSG00000005469	**CROT**	7.03	5.81	0.83	1.00E-04	1.8	9.36	15.57	1.66	5.00E-03	5.3
ENSG00000135929	**CYP27A1**	303.83	6.18	0.02	2.50E-08	1.0	374.56	19.40	0.05	8.89E-07	4.8
ENSG00000146233	**CYP39A1**	31.29	0.61	0.02	3.71E-07	0.4	24.01	0.92	0.04	4.43E-07	0.7
ENSG00000036530	**CYP46A1**	0.49	0.62	1.25	7.70E-03	0.9	0.59	0.72	1.23	NS	1.5
ENSG00000167910	**CYP7A1**	7.47	0.13	0.02	1.76E-06	0.0	6.67	0.01	0.00	5.91E-07	1.3
ENSG00000172817	**CYP7B1**	2.36	5.42	2.29	NS	2.1	2.76	6.99	2.53	NS	14.2
ENSG00000180432	**CYP8B1**	234.07	0.00	0.00	1.84E-10	0.0	194.32	0.11	0.00	4.09E-09	1.1
ENSG00000116133	**DHCR24**	379.13	61.59	0.16	2.34E-07	0.1	418.13	179.28	0.43	1.00E-04	0.3
ENSG00000211452	**DIO1**	116.20	0.17	0.00	5.87E-09	1.1	97.45	3.55	0.04	2.45E-06	12.9
ENSG00000211448	**DIO2**	0.00	0.28	85.76	1.20E-03	2.3	0.00	6.65	2388.71	9.00E-04	32.1
ENSG00000096093	**EFHC1**	2.31	12.02	5.20	NS	2.0	2.60	12.82	4.93	NS	1.4
ENSG00000120915	**EPHX2**	89.31	1.23	0.01	1.37E-07	0.3	100.87	4.30	0.04	8.65E-08	0.8
ENSG00000149485	**FADS1**	15.97	16.31	1.02	NS	1.0	12.07	46.72	3.87	NS	0.5
ENSG00000134824	**FADS2**	35.62	10.14	0.28	1.30E-03	0.1	29.15	45.89	1.57	NS	0.2
ENSG00000161513	**FDXR**	7.40	26.51	3.58	NS	2.1	4.26	39.61	9.30	NS	3.2
ENSG00000145321	**GC**	2359.12	0.00	0.00	8.36E-10	0.0	2207.74	0.55	0.00	1.10E-08	0.8
ENSG00000023909	**GCLM**	8.19	82.35	10.05	NS	5.4	7.58	97.64	12.89	NS	7.4

ENSG00000124713	**GNMT**	219.19	0.99	0.00	3.64E-08	0.4	218.39	0.98	0.00	8.88E-08	0.4
ENSG00000116906	**GNPAT**	15.97	61.23	3.83	NS	1.7	15.99	70.84	4.43	NS	1.2
ENSG00000197448	**GSTK1**	93.68	41.79	0.45	5.26E-06	3.8	90.10	49.25	0.55	0.00E+00	4.2
ENSG00000131373	**HACL1**	11.69	18.16	1.55	7.00E-04	0.5	18.49	26.67	1.44	2.90E-03	0.5
ENSG00000101323	**HAO1**	346.96	0.00	0.00	2.23E-10	0.0	337.73	0.03	0.00	5.12E-09	#DIV/0!
ENSG00000198189	**HSD17B11**	80.42	22.96	0.29	0.00E+00	2.1	62.49	44.90	0.72	1.00E-04	3.6
ENSG00000133835	**HSD17B4**	51.12	25.51	0.50	6.27E-06	0.3	63.61	45.17	0.71	0.00E+00	0.4
ENSG00000025423	**HSD17B6**	581.14	1.49	0.00	3.30E-09	0.3	725.95	1.78	0.00	5.87E-09	0.4
ENSG00000203857	**HSD3B1**	0.70	89.26	127.59	5.00E-04	80.0	0.09	151.81	1778.03	NS	11453.5
ENSG00000099377	**HSD3B7**	42.16	6.63	0.16	1.00E-06	1.3	42.13	6.76	0.16	1.49E-06	1.6
ENSG00000138413	**IDH1**	130.12	76.64	0.59	8.11E-06	0.3	125.10	176.61	1.41	1.20E-03	0.5
ENSG00000182054	**IDH2**	147.13	60.03	0.41	3.00E-04	1.0	62.77	63.12	1.01	3.00E-04	1.0
ENSG00000067064	**IDI1**	28.99	28.70	0.99	2.00E-04	0.3	58.11	47.73	0.82	2.20E-03	0.5
ENSG00000066583	**ISOC1**	43.83	61.62	1.41	1.00E-04	1.2	44.48	57.50	1.29	2.00E-04	1.2
ENSG00000105610	**KLF1**	0.14	0.10	0.72	NS	1.3	0.04	0.17	4.21	NS	0.3
ENSG00000182866	**LCK**	2.38	0.09	0.04	0.00E+00	0.9	0.66	0.05	0.08	0.00E+00	0.0
ENSG00000079435	**LIPE**	0.21	1.41	6.68	NS	1.6	0.09	1.44	16.32	NS	0.5
ENSG00000102910	**LONP2**	32.25	30.58	0.95	0.00E+00	3.0	41.68	34.48	0.83	1.00E-04	3.6
ENSG00000103150	**MLYCD**	4.14	2.53	0.61	5.56E-06	0.9	4.71	2.87	0.61	0.00E+00	1.1
ENSG00000069869	**NEDD4**	4.23	50.09	11.83	NS	8.8	5.03	46.84	9.31	NS	21.9
ENSG00000141458	**NPC1**	1.47	52.94	35.91	7.00E-04	4.2	1.57	60.55	38.59	7.00E-04	3.4
ENSG00000131910	**NR0B2**	109.38	0.00	0.00	8.13E-07	0.0	66.45	0.00	0.00	7.58E-07	#DIV/0!
ENSG00000012504	**NR1H4**	34.61	0.01	0.00	1.28E-08	0.0	33.22	0.01	0.00	1.91E-08	0.7
ENSG00000144852	**NR1I2**	57.81	0.01	0.00	1.51E-08	0.0	69.81	0.21	0.00	7.22E-07	0.3
ENSG00000151623	**NR3C2**	2.41	0.46	0.19	0.00E+00	0.6	2.45	1.90	0.77	1.50E-03	3.9
ENSG00000112874	**NUDT12**	7.49	17.43	2.33	2.50E-03	1.4	8.95	22.42	2.50	8.10E-03	1.5
ENSG00000123240	**OPTN**	26.13	125.47	4.80	NS	3.8	18.74	97.75	5.22	NS	10.0
ENSG00000148832	**PAOX**	10.27	6.69	0.65	1.00E-04	2.0	12.52	5.47	0.44	1.00E-04	1.5
ENSG00000115425	**PECR**	55.56	4.17	0.08	6.85E-08	0.5	56.85	6.68	0.12	8.24E-07	0.8
ENSG00000127980	**PEX1**	3.47	16.88	4.87	NS	1.6	3.30	17.53	5.32	NS	1.1
ENSG00000166821	**PEX11A**	12.81	6.17	0.48	3.03E-06	2.6	15.87	7.70	0.48	0.00E+00	2.8
ENSG00000104883	**PEX11G**	11.82	5.02	0.42	1.00E-04	2.4	12.99	3.40	0.26	1.00E-04	1.9
ENSG00000108733	**PEX12**	3.89	10.75	2.76	5.10E-03	1.9	3.94	11.92	3.02	NS	3.4

ENSG00000162928	**PEX13**	6.65	22.53	3.39	NS	2.0	6.59	23.05	3.50	NS	1.9
ENSG00000121680	**PEX16**	14.11	17.81	1.26	3.20E-03	1.1	15.83	16.57	1.05	2.20E-03	1.5
ENSG00000162735	**PEX19**	29.35	50.66	1.73	5.00E-04	1.2	32.92	56.32	1.71	9.00E-04	0.8
ENSG00000215193	**PEX26**	2.75	10.38	3.77	NS	1.6	2.44	11.65	4.78	NS	1.1
ENSG00000124587	**PEX6**	11.90	14.56	1.22	1.00E-04	2.6	12.10	22.53	1.86	7.00E-04	2.2
ENSG00000112357	**PEX7**	4.74	10.97	2.32	6.00E-03	1.4	4.72	11.50	2.44	9.50E-03	1.0
ENSG00000152556	**PFKM**	2.38	16.44	6.91	7.60E-03	1.2	1.58	27.10	17.18	1.10E-03	0.6
ENSG00000107537	**PHYH**	202.87	25.58	0.13	9.82E-07	2.0	216.53	28.19	0.13	2.26E-06	2.1
ENSG00000179761	**PIPOX**	180.84	0.36	0.00	5.71E-09	0.0	157.03	0.89	0.01	5.39E-08	0.0
ENSG00000135241	**PNPLA8**	4.72	38.15	8.09	NS	4.0	4.52	39.67	8.77	NS	4.7
ENSG00000126432	**PRDX5**	184.20	521.41	2.83	NS	1.5	220.45	590.59	2.68	NS	1.5
ENSG00000176894	**PXMP2**	132.71	20.71	0.16	7.44E-07	1.7	142.84	22.53	0.16	1.15E-06	2.1
ENSG00000114115	**RBP1**	13.72	75.28	5.49	NS	3.3	5.45	30.98	5.69	NS	2.6
ENSG00000042445	**RETSAT**	49.45	20.76	0.42	4.92E-06	1.8	55.79	39.89	0.71	1.00E-04	3.1
ENSG00000186350	**RXRA**	37.87	17.85	0.47	8.40E-06	1.7	47.92	24.39	0.51	0.00E+00	1.9
ENSG00000143171	**RXRG**	0.14	0.01	0.10	6.00E-04	0.1	0.07	0.15	2.08	NS	1.5
ENSG00000116171	**SCP2**	129.48	21.53	0.17	1.41E-06	1.7	122.10	26.00	0.21	2.51E-06	2.1
ENSG00000170099	**SERPINA6**	213.87	0.04	0.00	7.70E-10	0.0	167.78	0.14	0.00	7.24E-09	2.6
ENSG00000110628	**SLC22A18**	7.43	4.03	0.54	9.79E-06	10.8	7.55	7.81	1.04	2.00E-04	12.8
ENSG00000170482	**SLC23A1**	12.02	0.11	0.01	2.35E-07	0.0	15.55	0.39	0.02	2.71E-07	#DIV/0!
ENSG00000089057	**SLC23A2**	20.70	86.00	4.16	NS	2.5	33.84	66.50	1.96	NS	3.3
ENSG00000140284	**SLC27A2**	110.22	1.38	0.01	2.08E-08	0.1	111.65	11.67	0.10	1.85E-06	0.8
ENSG00000083807	**SLC27A5**	161.74	0.48	0.00	8.08E-09	0.2	186.96	0.99	0.01	1.96E-08	0.2
ENSG00000112759	**SLC29A1**	56.74	75.46	1.33	8.60E-03	1.1	52.36	115.34	2.20	NS	0.5
ENSG00000157593	**SLC35B2**	20.46	85.97	4.20	NS	2.0	17.70	98.59	5.57	NS	2.3
ENSG00000084453	**SLCO1A2**	0.18	0.76	4.26	NS	1.1	0.35	0.40	1.13	NS	0.3
ENSG00000167780	**SOAT2**	2.16	0.15	0.07	6.00E-04	1.6	4.98	4.94	0.99	NS	311.9
ENSG00000142168	**SOD1**	416.76	112.95	0.27	4.32E-06	0.8	569.21	148.84	0.26	9.53E-06	0.9
ENSG00000173597	**SULT1B1**	3.39	0.08	0.02	5.43E-06	0.0	3.60	0.93	0.26	2.00E-04	102.8
ENSG00000088002	**SULT2B1**	0.11	0.12	1.07	NS	1.0	0.02	0.09	4.28	NS	0.2
ENSG00000115112	**TFCP2L1**	0.16	10.55	67.49	2.30E-03	5.6	0.04	9.15	256.82	3.30E-03	4.9
ENSG00000118271	**TTR**	3202.86	5.69	0.00	2.59E-07	7.9	3471.91	404.78	0.12	0.00E+00	501.0

Hallmark Fatty Acid Metabolism

ENSG00000109576	**AADAT**	36.78	12.74	0.35	8.34E-07	1.1	34.48	24.07	0.70	9.60E-06	2.0
ENSG00000060971	**ACAA1**	102.67	22.99	0.22	2.50E-06	2.3	182.69	53.93	0.30	1.00E-04	5.6
ENSG00000167315	**ACAA2**	164.36	54.06	0.33	3.70E-06	0.8	209.38	71.71	0.34	9.71E-06	0.9
ENSG00000115361	**ACADL**	12.51	5.11	0.41	1.42E-06	27.9	11.90	12.63	1.06	9.00E-04	52.0
ENSG00000117054	**ACADM**	50.53	30.87	0.61	0.00E+00	0.8	51.62	43.00	0.83	1.00E-04	1.1
ENSG00000122971	**ACADS**	111.56	14.42	0.13	2.77E-07	2.7	119.35	11.09	0.09	7.99E-07	2.2
ENSG00000072778	**ACADVL**	276.00	245.76	0.89	3.20E-03	4.7	365.27	136.33	0.37	1.70E-03	2.5
ENSG00000120437	**ACAT2**	28.37	24.15	0.85	3.00E-04	0.2	50.99	45.02	0.88	5.40E-03	0.3
ENSG00000100412	**ACO2**	27.97	74.05	2.65	8.80E-03	1.2	22.70	108.94	4.80	NS	1.6
ENSG00000119673	**ACOT2**	46.24	7.14	0.15	0.00E+00	1.1	69.82	8.52	0.12	0.00E+00	1.0
ENSG00000101473	**ACOT8**	5.05	9.15	1.81	8.00E-04	0.8	5.71	10.61	1.86	2.40E-03	1.0
ENSG00000161533	**ACOX1**	55.87	17.86	0.32	7.51E-07	1.9	57.78	23.32	0.40	2.60E-06	2.8
ENSG00000151726	**ACSL1**	256.41	56.13	0.22	0.00E+00	8.0	291.26	48.01	0.16	0.00E+00	4.7
ENSG00000068366	**ACSL4**	2.51	57.27	22.80	NS	1.2	0.29	28.64	100.10	NS	0.7
ENSG00000197142	**ACSL5**	54.68	11.74	0.21	5.15E-07	#DIV/0!	65.06	4.67	0.07	4.00E-04	150.9
ENSG00000005187	**ACSM3**	10.16	6.51	0.64	3.00E-04	6.4	23.54	5.29	0.22	2.00E-04	3.8
ENSG00000154930	**ACSS1**	1.56	0.17	0.11	0.00E+00	0.1	0.60	1.24	2.07	1.00E-02	1.6
ENSG00000248144	**ADH1C**	944.48	0.00	0.00	1.11E-10	#DIV/0!	892.47	0.33	0.00	3.44E-09	2.3
ENSG00000196344	**ADH7**	0.21	0.00	0.00	1.54E-07	#DIV/0!	0.17	0.00	0.00	3.72E-07	0.0
ENSG00000006831	**ADIPOR2**	31.60	58.78	1.86	5.00E-04	1.7	39.29	57.87	1.47	5.00E-04	1.6
ENSG00000239900	**ADSL**	5.88	31.83	5.41	NS	0.4	5.90	37.72	6.40	NS	0.4
ENSG00000148218	**ALAD**	101.27	15.88	0.16	2.23E-07	1.7	114.46	24.47	0.21	7.73E-07	2.6
ENSG00000165092	**ALDH1A1**	346.80	1.14	0.00	5.24E-08	1.4	425.78	393.98	0.93	NS	290.7
ENSG00000108602	**ALDH3A1**	0.17	0.09	0.56	8.00E-03	0.5	0.35	0.10	0.29	4.50E-03	0.6
ENSG00000072210	**ALDH3A2**	60.13	12.01	0.20	1.56E-06	0.3	89.82	46.49	0.52	3.00E-04	1.4
ENSG00000143149	**ALDH9A1**	80.16	75.13	0.94	0.00E+00	0.9	89.10	72.28	0.81	0.00E+00	0.8
ENSG00000149925	**ALDOA**	15.46	301.59	19.51	0.00E+00	1.0	12.04	262.38	21.80	2.00E-04	0.9
ENSG00000131471	**AOC3**	5.36	2.31	0.43	1.00E-04	2.3	3.22	1.54	0.48	0.00E+00	1.1
ENSG00000100823	**APEX1**	60.52	220.08	3.64	NS	0.5	67.71	331.55	4.90	NS	0.5
ENSG00000165269	**AQP7**	14.29	0.05	0.00	3.42E-07	1.2	12.42	0.47	0.04	6.28E-06	15.0
ENSG00000148090	**AUH**	9.81	23.76	2.42	2.80E-03	1.3	9.00	18.76	2.09	3.30E-03	1.1
ENSG00000083123	**BCKDHB**	19.92	17.94	0.90	1.00E-04	1.0	20.99	19.84	0.95	2.00E-04	1.0
ENSG00000106605	**BLVRA**	2.48	21.85	8.81	NS	0.6	0.87	18.69	21.47	NS	0.6

ENSG00000138696	**BMPR1B**	0.21	1.85	8.71	5.60E-03	3.9	0.14	4.34	30.53	NS	4.3
ENSG00000137274	**BPHL**	43.37	12.37	0.29	0.00E+00	0.9	49.15	12.70	0.26	0.00E+00	0.9
ENSG00000104267	**CA2**	61.14	8.06	0.13	2.07E-06	0.7	69.17	28.19	0.41	1.00E-04	3.4
ENSG00000167434	**CA4**	0.03	0.80	26.43	7.00E-04	0.3	0.03	6.19	239.88	0.00E+00	3.4
ENSG00000159228	**CBR1**	106.87	28.48	0.27	4.13E-06	0.3	130.30	24.05	0.18	2.25E-06	0.3
ENSG00000159231	**CBR3**	0.31	2.62	8.35	NS	0.5	0.54	2.73	5.09	NS	0.8
ENSG00000160124	**CCDC58**	11.79	26.20	2.22	NS	0.6	9.87	33.14	3.36	NS	0.8
ENSG00000158473	**CD1D**	16.34	0.26	0.02	8.69E-07	0.1	14.16	0.72	0.05	4.82E-06	0.3
ENSG00000135218	**CD36**	3.83	0.01	0.00	1.93E-06	0.0	4.19	0.11	0.03	0.00E+00	0.3
ENSG00000170835	**CEL**	1.40	2.45	1.74	NS	1.3	1.10	12.62	11.50	NS	5.0
ENSG00000080819	**CPOX**	16.64	43.41	2.61	3.30E-03	1.6	22.58	40.24	1.78	3.40E-03	1.4
ENSG00000110090	**CPT1A**	13.01	33.13	2.55	NS	8.8	18.15	13.12	0.72	7.00E-04	3.7
ENSG00000157184	**CPT2**	39.48	19.74	0.50	6.90E-06	0.6	31.01	20.91	0.67	0.00E+00	0.7
ENSG00000095321	**CRAT**	66.79	17.71	0.27	1.34E-06	1.9	76.38	17.51	0.23	2.34E-06	1.8
ENSG00000116791	**CRYZ**	55.88	39.26	0.70	0.00E+00	1.3	52.36	25.01	0.48	1.00E-04	1.3
ENSG00000140465	**CYP1A1**	4.46	14.33	3.21	NS	25.6	21.25	34.58	1.63	NS	42.8
ENSG00000187048	**CYP4A11**	419.96	0.00	0.00	2.12E-10	0.0	555.30	0.11	0.00	2.03E-09	1.2
ENSG00000162365	**CYP4A22**	187.60	0.00	0.00	2.93E-10	0.0	188.06	0.07	0.00	1.30E-09	1.3
ENSG00000180902	**D2HGDH**	37.09	6.90	0.19	2.30E-03	1.5	47.17	9.84	0.21	3.90E-03	1.6
ENSG00000104325	**DECR1**	110.40	12.49	0.11	2.25E-07	0.6	82.53	14.53	0.18	6.47E-07	0.8
ENSG00000116133	**DHCR24**	379.13	61.59	0.16	2.34E-07	0.1	418.13	179.28	0.43	1.00E-04	0.3
ENSG00000091140	**DLD**	18.16	68.13	3.75	NS	1.3	18.15	69.14	3.81	NS	1.2
ENSG00000119689	**DLST**	22.97	105.59	4.60	NS	1.5	23.34	131.37	5.63	NS	1.3
ENSG00000104823	**ECH1**	172.35	52.06	0.30	4.18E-06	0.7	215.23	70.43	0.33	0.00E+00	0.8
ENSG00000127884	**ECHS1**	768.10	128.10	0.17	1.59E-06	0.8	763.36	172.03	0.23	5.05E-06	1.0
ENSG00000167969	**ECI1**	35.29	62.63	1.77	1.20E-03	2.3	37.84	65.78	1.74	2.10E-03	2.7
ENSG00000198721	**ECI2**	63.29	29.88	0.47	3.75E-06	0.9	64.61	31.74	0.49	7.99E-06	0.9
ENSG00000113790	**EHHADH**	139.27	7.80	0.06	2.61E-07	3.0	146.48	11.28	0.08	4.72E-07	5.1
ENSG00000012660	**ELOVL5**	24.80	140.67	5.67	NS	1.0	27.04	128.98	4.77	NS	0.9
ENSG00000111674	**ENO2**	1.65	5.52	3.34	NS	0.1	0.69	46.60	67.36	NS	0.8
ENSG00000108515	**ENO3**	109.23	2.28	0.02	2.70E-06	0.4	61.98	3.62	0.06	5.38E-06	0.5
ENSG00000143819	**EPHX1**	646.92	50.60	0.08	6.73E-08	0.9	700.07	111.99	0.16	4.84E-06	1.9
ENSG00000089248	**ERP29**	89.65	216.53	2.42	NS	1.7	93.53	280.32	3.00	NS	2.0

ENSG00000171503	**ETFDH**	31.63	7.04	0.22	0.00E+00	1.1	31.97	11.69	0.37	0.00E+00	1.6
ENSG00000163586	**FABP1**	833.54	0.87	0.00	3.11E-08	32.2	792.69	2.37	0.00	2.21E-06	16.0
ENSG00000169710	**FASN**	47.98	71.52	1.49	9.80E-03	0.2	132.73	113.10	0.85	NS	0.3
ENSG00000091483	**FH**	84.62	113.49	1.34	1.00E-04	1.1	89.92	106.04	1.18	1.00E-04	1.0
ENSG00000010932	**FMO1**	0.31	0.47	1.53	NS	25.9	0.12	3.63	31.37	4.10E-03	97.7
ENSG00000123689	**G0S2**	51.68	102.15	1.98	NS	191.4	63.54	27.10	0.43	2.00E-03	54.1
ENSG00000139112	**GABARAPL 1**	45.19	134.94	2.99	NS	2.4	39.32	151.39	3.85	NS	3.1
ENSG00000136750	**GAD2**	0.00	0.01	3.23	NS	0.0	0.00	0.46	134.20	NS	1.0
ENSG00000105607	**GCDH**	34.09	8.91	0.26	4.47E-06	0.3	28.04	11.27	0.40	8.92E-06	0.4
ENSG00000135821	**GLUL**	123.27	68.81	0.56	5.81E-06	0.7	164.94	90.20	0.55	0.00E+00	0.9
ENSG00000167588	**GPD1**	72.90	0.59	0.01	6.93E-09	1.3	82.65	0.92	0.01	1.65E-08	1.9
ENSG00000115159	**GPD2**	1.07	38.54	36.08	2.00E-04	1.2	0.70	46.91	66.77	2.00E-04	1.4
ENSG00000137106	**GRHPR**	123.57	26.69	0.22	2.11E-06	1.3	133.12	34.88	0.26	5.48E-06	1.4
ENSG00000100577	**GSTZ1**	24.55	5.45	0.22	3.19E-06	0.8	24.23	7.71	0.32	7.91E-06	0.7
ENSG00000164032	**H2AFZ**	15.79	316.89	20.07	0.00E+00	0.5	16.82	403.56	23.99	1.00E-04	0.6
ENSG00000138796	**HADH**	61.90	34.63	0.56	7.92E-06	0.7	74.19	28.42	0.38	0.00E+00	0.6
ENSG00000138029	**HADHB**	58.30	105.93	1.82	9.00E-04	1.9	63.89	67.51	1.06	2.00E-04	1.6
ENSG00000116882	**HAO2**	217.00	0.06	0.00	1.41E-09	0.5	229.60	1.41	0.01	6.79E-06	6.2
ENSG00000004961	**HCCS**	5.51	45.81	8.31	NS	1.2	4.65	51.00	10.98	NS	1.3
ENSG00000198130	**HIBCH**	20.91	5.32	0.25	1.99E-06	0.5	17.45	7.74	0.44	7.67E-06	0.7
ENSG00000117305	**HMGCL**	66.64	15.46	0.23	1.46E-06	4.0	62.44	21.53	0.34	4.92E-06	4.6
ENSG00000112972	**HMGCS1**	27.60	39.38	1.43	1.50E-03	0.2	95.73	68.95	0.72	7.00E-03	0.2
ENSG00000134240	**HMGCS2**	874.78	2.85	0.00	1.00E-06	73.8	1059.55	27.36	0.03	1.64E-07	133.9
ENSG00000164120	**HPGD**	51.60	14.04	0.27	0.00E+00	18.6	30.41	46.87	1.54	4.90E-03	68.2
ENSG00000072506	**HSD17B10**	139.49	95.30	0.68	0.00E+00	0.7	149.84	120.90	0.81	1.00E-04	0.8
ENSG00000198189	**HSD17B11**	80.42	22.96	0.29	0.00E+00	2.1	62.49	44.90	0.72	1.00E-04	3.6
ENSG00000133835	**HSD17B4**	51.12	25.51	0.50	6.27E-06	0.3	63.61	45.17	0.71	0.00E+00	0.4
ENSG00000132196	**HSD17B7**	5.40	5.18	0.96	2.00E-04	0.5	10.19	5.09	0.50	3.00E-04	0.8
ENSG00000119471	**HSDL2**	49.55	52.23	1.05	1.00E-04	2.9	60.67	82.52	1.36	4.00E-04	3.0
ENSG00000080824	**HSP90AA1**	44.35	711.45	16.04	NS	1.0	87.59	1001.62	11.43	NS	1.0
ENSG00000120694	**HSPH1**	4.01	91.95	22.93	NS	1.2	7.09	85.66	12.08	NS	1.0
ENSG00000138413	**IDH1**	130.12	76.64	0.59	8.11E-06	0.3	125.10	176.61	1.41	1.20E-03	0.5
ENSG00000101365	**IDH3B**	28.77	68.35	2.38	NS	0.7	32.29	79.82	2.47	NS	0.8

ENSG00000067829	**IDH3G**	14.49	21.50	1.48	7.60E-03	0.8	14.65	29.45	2.01	NS	1.0
ENSG00000067064	**IDI1**	28.99	28.70	0.99	2.00E-04	0.3	58.11	47.73	0.82	2.20E-03	0.5
ENSG00000104951	**IL4I1**	0.37	1.92	5.16	NS	1.0	0.07	2.75	37.59	NS	1.3
ENSG00000241644	**INMT**	9.42	0.35	0.04	6.12E-06	0.9	3.09	1.53	0.49	1.00E-04	3.6
ENSG00000134333	**LDHA**	76.76	111.88	1.46	2.00E-04	0.5	89.44	90.66	1.01	2.00E-04	0.4
ENSG00000100097	**LGALS1**	47.85	5.94	0.12	6.45E-06	0.2	86.85	4.22	0.05	4.19E-06	0.3
ENSG00000213316	**LTC4S**	1.52	0.98	0.64	2.10E-03	1.9	1.63	0.61	0.38	6.00E-04	1.4
ENSG00000189221	**MAOA**	48.58	2.71	0.06	5.37E-07	0.6	46.58	3.50	0.08	9.56E-07	0.7
ENSG00000124370	**MCEE**	14.81	5.91	0.40	3.37E-06	0.8	16.45	7.38	0.45	0.00E+00	1.0
ENSG00000014641	**MDH1**	29.38	72.08	2.45	2.00E-03	0.5	31.13	102.58	3.29	NS	0.7
ENSG00000146701	**MDH2**	50.71	106.63	2.10	7.00E-04	0.7	49.98	122.38	2.45	2.80E-03	0.8
ENSG00000065833	**ME1**	3.30	38.69	11.72	2.50E-03	5.0	2.47	78.78	31.91	3.00E-04	10.6
ENSG00000164024	**METAP1**	7.83	76.43	9.76	NS	0.9	9.80	74.59	7.61	NS	0.8
ENSG00000074416	**MGLL**	21.04	21.20	1.01	1.00E-04	130.3	15.27	9.76	0.64	5.00E-04	64.8
ENSG00000240972	**MIF**	50.35	111.56	2.22	5.30E-03	0.5	78.77	130.61	1.66	NS	0.6
ENSG00000103150	**MLYCD**	4.14	2.53	0.61	5.56E-06	0.9	4.71	2.87	0.61	0.00E+00	1.1
ENSG00000104320	**NBN**	5.20	21.89	4.21	NS	0.8	4.29	23.35	5.45	NS	0.8
ENSG00000025770	**NCAPH2**	10.02	59.88	5.97	NS	1.3	12.09	73.68	6.09	NS	1.4
ENSG00000147383	**NSDHL**	13.39	31.03	2.32	6.20E-03	0.6	24.15	43.09	1.78	NS	0.8
ENSG00000065057	**NTHL1**	18.01	6.76	0.38	3.00E-04	0.2	25.09	9.33	0.37	5.00E-04	0.2
ENSG00000115758	**ODC1**	8.14	320.45	39.35	0.00E+00	2.5	7.10	328.92	46.34	1.00E-04	3.0
ENSG00000198856	**OSTC**	57.44	268.86	4.68	NS	1.5	64.56	252.65	3.91	NS	1.2
ENSG00000166228	**PCBD1**	157.37	48.31	0.31	3.61E-06	2.3	205.04	85.13	0.42	0.00E+00	2.7
ENSG00000131828	**PDHA1**	27.39	55.63	2.03	1.20E-03	0.9	27.95	66.24	2.37	5.10E-03	1.0
ENSG00000168291	**PDHB**	17.51	39.14	2.24	2.30E-03	0.4	22.22	53.05	2.39	NS	0.5
ENSG00000186951	**PPARA**	19.13	8.93	0.47	0.00E+00	4.6	21.65	11.64	0.54	1.00E-04	6.5
ENSG00000117592	**PRDX6**	236.83	520.02	2.20	2.00E-03	0.9	272.43	667.41	2.45	NS	1.2
ENSG00000092010	**PSME1**	83.56	117.90	1.41	1.00E-04	1.6	87.23	81.68	0.94	5.00E-04	0.8
ENSG00000144724	**PTPRG**	1.22	15.32	12.52	NS	0.5	1.43	17.84	12.51	NS	0.5
ENSG00000150787	**PTS**	13.44	46.23	3.44	NS	4.2	8.06	49.80	6.18	NS	4.8
ENSG00000138698	**RAP1GDS1**	4.43	35.98	8.12	NS	2.2	6.12	37.39	6.11	NS	2.1
ENSG00000072042	**RDH11**	35.49	45.53	1.28	6.00E-04	0.6	31.24	73.63	2.36	NS	0.7
ENSG00000139547	**RDH16**	160.54	0.70	0.00	1.39E-08	1.8	195.98	0.98	0.00	3.23E-08	1.9

ENSG00000115255	**REEP6**	151.23	3.54	0.02	6.89E-07	0.3	179.89	23.17	0.13	0.00E+00	1.8
ENSG00000042445	**RETSAT**	49.45	20.76	0.42	4.92E-06	1.8	55.79	39.89	0.71	1.00E-04	3.1
ENSG00000197747	**S100A10**	50.26	596.97	11.88	4.00E-03	5.0	23.66	651.20	27.53	4.30E-03	11.2
ENSG00000073578	**SDHA**	33.50	26.22	0.78	0.00E+00	0.9	35.16	30.57	0.87	0.00E+00	1.3
ENSG00000143252	**SDHC**	11.70	20.81	1.78	7.00E-04	1.1	11.65	22.88	1.96	1.30E-03	1.3
ENSG00000204370	**SDHD**	60.21	56.04	0.93	1.00E-04	0.8	61.49	62.95	1.02	1.00E-04	0.9
ENSG00000111897	**SERINC1**	78.83	297.69	3.78	NS	4.9	65.47	391.24	5.98	NS	5.8
ENSG00000197375	**SLC22A5**	2.41	17.69	7.35	NS	11.5	2.58	10.94	4.24	NS	7.4
ENSG00000102172	**SMS**	13.98	483.48	34.58	4.41E-06	1.4	13.01	238.15	18.31	1.90E-03	1.0
ENSG00000136143	**SUCLA2**	6.39	51.27	8.02	NS	1.0	7.36	75.72	10.28	NS	1.1
ENSG00000163541	**SUCLG1**	30.55	27.81	0.91	0.00E+00	1.4	25.33	39.15	1.55	3.00E-04	1.9
ENSG00000172340	**SUCLG2**	76.41	32.09	0.42	0.00E+00	0.7	74.04	49.31	0.67	1.00E-04	0.8
ENSG00000151790	**TDO2**	81.84	0.04	0.00	1.35E-06	3.4	56.20	1.23	0.02	5.33E-07	24.6
ENSG00000078804	**TP53INP2**	17.34	37.49	2.16	9.60E-03	6.7	15.35	63.01	4.10	NS	11.8
ENSG00000156587	**UBE2L6**	33.52	50.17	1.50	1.10E-03	0.8	25.91	32.27	1.25	1.20E-03	0.5
ENSG00000109814	**UGDH**	15.02	58.13	3.87	NS	1.0	19.49	53.10	2.72	NS	0.9
ENSG00000126088	**UROD**	43.10	51.55	1.20	1.00E-03	0.9	50.86	66.83	1.31	3.30E-03	1.0
ENSG00000188690	**UROS**	6.50	11.67	1.79	1.60E-03	0.6	6.34	14.02	2.21	4.80E-03	0.8
ENSG00000112299	**VNN1**	77.81	0.04	0.00	2.59E-08	#DIV/0!	63.72	0.06	0.00	7.48E-08	2.7
ENSG00000229807	**XIST**	38.62	1.73	0.04	NS	0.3	0.00	0.02	23.48	NS	0.6
ENSG00000128245	**YWHAH**	12.22	226.42	18.52	1.00E-04	0.9	10.02	211.14	21.08	4.00E-04	0.8

Hallmark Adipogenesis

ENSG00000165029	**ABCA1**	16.96	15.04	0.89	1.60E-03	1.5	10.22	88.06	8.62	NS	11.7
ENSG00000197150	**ABCB8**	4.71	13.12	2.78	NS	2.2	4.41	13.72	3.11	NS	2.2
ENSG00000167315	**ACAA2**	164.36	54.06	0.33	3.70E-06	0.8	209.38	71.71	0.34	9.71E-06	0.9
ENSG00000115361	**ACADL**	12.51	5.11	0.41	1.42E-06	27.9	11.90	12.63	1.06	9.00E-04	52.0
ENSG00000117054	**ACADM**	50.53	30.87	0.61	0.00E+00	0.8	51.62	43.00	0.83	1.00E-04	1.1
ENSG00000122971	**ACADS**	111.56	14.42	0.13	2.77E-07	2.7	119.35	11.09	0.09	7.99E-07	2.2
ENSG00000131473	**ACLY**	5.26	141.93	27.00	2.00E-04	0.4	6.08	197.91	32.57	1.00E-04	0.5
ENSG00000100412	**ACO2**	27.97	74.05	2.65	8.80E-03	1.2	22.70	108.94	4.80	NS	1.6
ENSG00000161533	**ACOX1**	55.87	17.86	0.32	7.51E-07	1.9	57.78	23.32	0.40	2.60E-06	2.8
ENSG00000174233	**ADCY6**	8.74	35.74	4.09	NS	3.3	11.83	37.41	3.16	NS	3.0
ENSG00000006831	**ADIPOR2**	31.60	58.78	1.86	5.00E-04	1.7	39.29	57.87	1.47	5.00E-04	1.6

ENSG00000160216	**AGPAT3**	17.83	20.85	1.17	0.00E+00	4.7	18.84	31.16	1.65	9.00E-04	15.7
ENSG00000156709	**AIFM1**	40.28	36.11	0.90	0.00E+00	0.6	39.59	41.89	1.06	0.00E+00	0.7
ENSG00000004455	**AK2**	30.52	44.94	1.47	1.00E-04	1.0	35.58	48.69	1.37	5.00E-04	1.0
ENSG00000111275	**ALDH2**	1213.55	120.45	0.10	1.65E-07	2.0	1511.06	187.48	0.12	7.77E-07	3.2
ENSG00000149925	**ALDOA**	15.46	301.59	19.51	0.00E+00	1.0	12.04	262.38	21.80	2.00E-04	0.9
ENSG00000154188	**ANGPT1**	0.25	0.10	0.39	5.30E-03	0.1	0.06	1.52	23.56	NS	2.0
ENSG00000167772	**ANGPTL4**	9.42	83.96	8.91	NS	72.2	18.56	31.54	1.70	NS	21.5
ENSG00000084234	**APLP2**	83.20	359.75	4.32	NS	1.8	78.12	914.32	11.70	NS	4.1
ENSG00000130203	**APOE**	2589.24	449.37	0.17	0.00E+00	1.3	2690.60	1522.84	0.57	6.00E-04	4.1
ENSG00000078061	**ARAF**	34.82	44.94	1.29	5.00E-04	1.6	37.63	64.34	1.71	4.30E-03	2.4
ENSG00000122644	**ARL4A**	1.60	6.51	4.08	NS	0.4	1.00	25.00	24.96	8.90E-03	1.4
ENSG00000119787	**ATL2**	13.37	41.50	3.10	7.90E-03	0.6	16.21	40.69	2.51	9.90E-03	0.8
ENSG00000069849	**ATP1B3**	2.05	122.61	59.80	1.00E-04	0.7	1.34	148.16	110.57	1.00E-04	0.8
ENSG00000241837	**ATP5O**	33.14	81.18	2.45	8.60E-03	0.7	39.23	121.68	3.10	NS	0.9
ENSG00000076108	**BAZ2A**	8.35	64.58	7.73	NS	1.3	11.50	72.89	6.34	NS	1.3
ENSG00000248098	**BCKDHA**	28.86	24.95	0.86	0.00E+00	0.8	28.99	27.68	0.95	0.00E+00	0.9
ENSG00000099968	**BCL2L13**	6.64	27.93	4.20	NS	1.4	7.42	30.38	4.10	NS	1.5
ENSG00000113916	**BCL6**	10.74	25.69	2.39	NS	10.4	10.04	27.15	2.71	NS	15.1
ENSG00000125730	**C3**	1773.54	14.49	0.01	1.54E-07	5.7	1456.62	7.61	0.01	0.00E+00	3.3
ENSG00000121691	**CAT**	341.00	10.86	0.03	1.54E-07	0.9	400.58	21.17	0.05	5.49E-07	1.5
ENSG00000138764	**CCNG2**	1.92	53.86	28.11	0.00E+00	12.0	1.29	26.95	20.92	NS	4.6
ENSG00000177697	**CD151**	44.89	104.06	2.32	NS	2.1	25.07	110.90	4.42	NS	2.3
ENSG00000241399	**CD302**	73.86	1.52	0.02	2.46E-07	1.3	62.93	4.20	0.07	6.66E-07	3.9
ENSG00000135218	**CD36**	3.83	0.01	0.00	1.93E-06	0.0	4.19	0.11	0.03	0.00E+00	0.3
ENSG00000123080	**CDKN2C**	0.62	0.44	0.72	1.00E-04	8.7	1.14	0.68	0.60	2.00E-04	11.4
ENSG00000250479	**CHCHD10**	42.30	3.53	0.08	1.47E-06	0.2	73.23	21.18	0.29	3.00E-04	1.0
ENSG00000213341	**CHUK**	2.97	18.85	6.35	NS	1.3	2.90	23.11	7.96	NS	1.6
ENSG00000164237	**CMBL**	113.11	17.00	0.15	2.82E-06	0.9	157.50	54.45	0.35	2.00E-04	2.8
ENSG00000162368	**CMPK1**	60.26	97.13	1.61	2.00E-04	2.7	63.67	108.52	1.70	5.00E-04	2.8
ENSG00000204291	**COL15A1**	0.25	1.91	7.64	NS	9.9	0.08	2.45	28.94	NS	8.9
ENSG00000187498	**COL4A1**	2.57	84.32	32.81	NS	1.6	1.24	601.28	483.68	1.20E-03	10.7
ENSG00000132423	**COQ3**	8.91	5.89	0.66	0.00E+00	0.2	8.83	10.75	1.22	5.00E-04	0.4
ENSG00000110871	**COQ5**	19.73	47.88	2.43	3.20E-03	1.7	21.09	53.97	2.56	9.10E-03	1.6

ENSG00000088682	**COQ9**	17.34	14.19	0.82	0.00E+00	0.9	17.82	18.98	1.07	1.00E-04	1.3
ENSG00000111775	**COX6A1**	133.21	163.36	1.23	8.00E-04	0.9	128.27	245.19	1.91	8.50E-03	1.4
ENSG00000131174	**COX7B**	34.01	31.18	0.92	1.00E-04	0.5	38.36	39.93	1.04	4.00E-04	0.7
ENSG00000176340	**COX8A**	233.95	388.62	1.66	5.80E-03	0.7	243.47	523.06	2.15	NS	0.9
ENSG00000157184	**CPT2**	39.48	19.74	0.50	6.90E-06	0.6	31.01	20.91	0.67	0.00E+00	0.7
ENSG00000095321	**CRAT**	66.79	17.71	0.27	1.34E-06	1.9	76.38	17.51	0.23	2.34E-06	1.8
ENSG00000062485	**CS**	19.58	97.81	5.00	NS	1.0	15.24	98.93	6.49	NS	1.0
ENSG00000179091	**CYC1**	45.67	81.05	1.77	3.00E-04	0.6	56.58	115.77	2.05	3.40E-03	0.8
ENSG00000137992	**DBT**	5.94	10.10	1.70	4.20E-03	1.1	6.06	11.55	1.91	5.70E-03	1.1
ENSG00000099977	**DDT**	98.30	17.01	0.17	4.12E-06	0.7	135.98	28.72	0.21	0.00E+00	1.2
ENSG00000104325	**DECR1**	110.40	12.49	0.11	2.25E-07	0.6	82.53	14.53	0.18	6.47E-07	0.8
ENSG00000185000	**DGAT1**	27.63	11.02	0.40	0.00E+00	1.0	30.40	21.06	0.69	4.00E-04	1.9
ENSG00000172893	**DHCR7**	54.07	9.52	0.18	0.00E+00	0.1	57.04	35.68	0.63	1.10E-03	0.4
ENSG00000100612	**DHRS7**	21.15	39.71	1.88	1.00E-03	2.9	20.77	57.34	2.76	8.50E-03	2.7
ENSG00000109016	**DHRS7B**	3.35	9.60	2.86	NS	2.1	3.11	12.61	4.06	NS	2.5
ENSG00000150768	**DLAT**	5.35	38.66	7.22	NS	0.6	3.44	58.86	17.13	NS	0.9
ENSG00000091140	**DLD**	18.16	68.13	3.75	NS	1.3	18.15	69.14	3.81	NS	1.2
ENSG00000128590	**DNAJB9**	18.34	71.72	3.91	NS	4.9	32.82	62.78	1.91	NS	3.6
ENSG00000120675	**DNAJC15**	6.62	25.50	3.85	NS	3.0	7.13	50.48	7.08	NS	5.6
ENSG00000156171	**DRAM2**	10.45	68.64	6.57	NS	3.3	13.05	72.15	5.53	NS	3.3
ENSG00000104823	**ECH1**	172.35	52.06	0.30	4.18E-06	0.7	215.23	70.43	0.33	0.00E+00	0.8
ENSG00000127884	**ECHS1**	768.10	128.10	0.17	1.59E-06	0.8	763.36	172.03	0.23	5.05E-06	1.0
ENSG00000115459	**ELMOD3**	5.46	8.02	1.47	8.10E-03	2.9	5.51	9.40	1.71	NS	3.5
ENSG00000170522	**ELOVL6**	12.45	29.92	2.40	6.70E-03	0.5	19.83	23.14	1.17	4.70E-03	0.3
ENSG00000136960	**ENPP2**	6.48	1.43	0.22	NS	1.3	0.61	16.02	26.11	NS	6.3
ENSG00000120915	**EPHX2**	89.31	1.23	0.01	1.37E-07	0.3	100.87	4.30	0.04	8.65E-08	0.8
ENSG00000173153	**ESRRA**	25.00	29.32	1.17	2.00E-04	1.3	26.17	29.48	1.13	3.00E-04	1.2
ENSG00000139641	**ESYT1**	22.20	132.87	5.98	NS	1.5	19.76	126.47	6.40	NS	1.5
ENSG00000105379	**ETFB**	59.67	6.80	0.11	5.10E-06	0.9	67.99	17.95	0.26	0.00E+00	1.7
ENSG00000170323	**FABP4**	1.29	0.00	0.00	1.00E-04	0.0	0.24	0.00	0.00	1.00E-04	#DIV/0!
ENSG00000103876	**FAH**	41.82	12.60	0.30	1.83E-06	2.3	53.48	9.19	0.17	0.00E+00	1.5
ENSG00000174804	**FZD4**	13.82	5.78	0.42	0.00E+00	0.7	12.39	51.91	4.19	NS	7.9
ENSG00000138757	**G3BP2**	9.25	96.73	10.45	NS	0.5	8.58	122.57	14.29	NS	0.7

ENSG00000116717	**GADD45A**	42.67	188.41	4.42	NS	23.6	45.42	189.69	4.18	NS	21.9
ENSG00000114480	**GBE1**	21.86	30.94	1.42	8.00E-04	4.4	17.66	36.42	2.06	1.50E-03	4.3
ENSG00000165678	**GHITM**	115.60	380.54	3.29	NS	2.3	112.97	429.12	3.80	NS	2.7
ENSG00000119927	**GPAM**	54.08	13.38	0.25	0.00E+00	0.9	68.46	13.78	0.20	0.00E+00	1.0
ENSG00000115159	**GPD2**	1.07	38.54	36.08	2.00E-04	1.2	0.70	46.91	66.77	2.00E-04	1.4
ENSG00000171723	**GPHN**	6.45	7.76	1.20	2.00E-04	0.9	9.49	11.09	1.17	6.00E-04	0.9
ENSG00000211445	**GPX3**	108.25	4.71	0.04	1.80E-07	0.2	70.65	67.16	0.95	2.00E-03	1.7
ENSG00000167468	**GPX4**	197.85	209.81	1.06	1.60E-03	1.2	286.71	358.68	1.25	NS	1.9
ENSG00000109519	**GRPEL1**	12.54	30.35	2.42	1.90E-03	1.2	15.06	23.99	1.59	6.00E-04	0.9
ENSG00000138796	**HADH**	61.90	34.63	0.56	7.92E-06	0.7	74.19	28.42	0.38	0.00E+00	0.6
ENSG00000198130	**HIBCH**	20.91	5.32	0.25	1.99E-06	0.5	17.45	7.74	0.44	7.67E-06	0.7
ENSG00000152137	**HSPB8**	0.48	184.76	386.48	1.00E-04	7.7	0.25	248.70	1002.98	1.00E-04	16.2
ENSG00000138413	**IDH1**	130.12	76.64	0.59	8.11E-06	0.3	125.10	176.61	1.41	1.20E-03	0.5
ENSG00000166411	**IDH3A**	3.98	15.02	3.77	NS	1.1	4.31	15.70	3.64	NS	1.3
ENSG00000067829	**IDH3G**	14.49	21.50	1.48	7.60E-03	0.8	14.65	29.45	2.01	NS	1.0
ENSG00000027697	**IFNGR1**	22.46	116.91	5.21	NS	3.6	15.81	156.62	9.91	NS	4.1
ENSG00000132305	**IMMT**	18.44	101.58	5.51	NS	1.1	17.81	110.07	6.18	NS	1.0
ENSG00000135424	**ITGA7**	1.95	2.66	1.36	2.90E-03	0.1	1.51	9.25	6.14	NS	0.3
ENSG00000123243	**ITIH5**	0.44	0.80	1.81	NS	0.5	0.16	3.68	22.82	NS	2.4
ENSG00000205726	**ITSN1**	3.35	15.73	4.69	NS	0.8	3.24	19.10	5.89	NS	1.0
ENSG00000171135	**JAGN1**	30.35	52.00	1.71	3.00E-03	1.2	30.99	54.69	1.76	4.00E-03	1.3
ENSG00000112769	**LAMA4**	1.17	0.04	0.03	6.70E-06	0.1	0.49	0.41	0.84	6.00E-04	0.8
ENSG00000113594	**LIFR**	7.24	32.21	4.45	NS	4.1	3.72	124.40	33.42	NS	14.6
ENSG00000079435	**LIPE**	0.21	1.41	6.68	NS	0.4	0.09	1.44	16.32	NS	0.5
ENSG00000111684	**LPCAT3**	23.02	34.58	1.50	2.00E-04	0.9	23.42	41.17	1.76	7.00E-04	1.0
ENSG00000175445	**LPL**	0.08	44.52	532.69	7.00E-04	6.4	0.00	24.00	5041.67	3.30E-03	2.3
ENSG00000213316	**LTC4S**	1.52	0.98	0.64	2.10E-03	1.9	1.63	0.61	0.38	6.00E-04	1.4
ENSG00000011566	**MAP4K3**	4.43	68.01	15.36	5.40E-03	1.3	3.96	42.82	10.80	NS	0.9
ENSG00000078070	**MCCC1**	21.70	15.40	0.71	0.00E+00	0.8	17.80	20.12	1.13	1.00E-04	1.2
ENSG00000146701	**MDH2**	50.71	106.63	2.10	7.00E-04	0.7	49.98	122.38	2.45	2.80E-03	0.8
ENSG00000065833	**ME1**	3.30	38.69	11.72	2.50E-03	5.0	2.47	78.78	31.91	3.00E-04	10.6
ENSG00000074416	**MGLL**	21.04	21.20	1.01	1.00E-04	130.3	15.27	9.76	0.64	5.00E-04	64.8
ENSG00000143198	**MGST3**	7.32	29.56	4.04	NS	2.8	9.02	25.79	2.86	NS	1.9

ENSG00000137547	**MRPL15**	28.47	166.99	5.86	NS	1.1	28.23	146.95	5.21	NS	0.9
ENSG00000109919	**MTCH2**	73.38	92.51	1.26	1.00E-04	1.0	81.91	120.31	1.47	5.00E-04	1.4
ENSG00000065534	**MYLK**	6.76	39.77	5.88	NS	9.7	5.71	11.75	2.06	NS	2.4
ENSG00000128609	**NDUFA5**	8.10	14.84	1.83	1.00E-03	0.8	7.30	16.99	2.33	3.00E-03	0.9
ENSG00000004779	**NDUFAB1**	42.03	72.13	1.72	2.80E-03	0.6	55.73	92.43	1.66	NS	0.9
ENSG00000099795	**NDUFB7**	171.09	200.80	1.17	3.20E-03	1.0	226.45	235.57	1.04	5.50E-03	1.2
ENSG00000213619	**NDUFS3**	21.86	41.48	1.90	9.40E-03	0.8	27.19	48.09	1.77	NS	0.9
ENSG00000197885	**NKIRAS1**	3.77	7.68	2.04	6.00E-04	1.9	4.90	9.82	2.00	3.50E-03	2.0
ENSG00000136448	**NMT1**	19.97	78.89	3.95	NS	1.1	20.90	95.20	4.55	NS	1.2
ENSG00000229314	**ORM1**	9562.54	0.18	0.00	6.16E-10	0.5	5834.23	2.41	0.00	1.25E-08	1.4
ENSG00000150593	**PDCD4**	15.03	54.95	3.65	NS	4.7	12.85	43.46	3.38	NS	4.0
ENSG00000133027	**PEMT**	64.28	19.85	0.31	1.14E-06	0.6	76.71	19.92	0.26	1.94E-06	0.6
ENSG00000142655	**PEX14**	13.22	27.16	2.05	NS	1.6	14.49	34.96	2.41	NS	2.1
ENSG00000170525	**PFKFB3**	11.53	49.90	4.33	NS	2.4	1.62	49.73	30.60	NS	2.3
ENSG00000141959	**PFKL**	21.56	68.92	3.20	NS	2.3	22.41	62.65	2.80	NS	1.9
ENSG00000079739	**PGM1**	90.55	47.35	0.52	6.54E-06	0.9	104.33	59.08	0.57	0.00E+00	1.7
ENSG00000019144	**PHLDB1**	0.78	8.65	11.12	1.80E-03	1.1	0.50	10.83	21.47	2.30E-03	1.0
ENSG00000107537	**PHYH**	202.87	25.58	0.13	9.82E-07	2.0	216.53	28.19	0.13	2.26E-06	2.1
ENSG00000198355	**PIM3**	17.46	51.38	2.94	NS	1.9	20.67	49.04	2.37	NS	3.1
ENSG00000147872	**PLIN2**	59.84	213.27	3.56	NS	12.0	50.42	139.53	2.77	NS	6.5
ENSG00000127948	**POR**	54.92	44.06	0.80	0.00E+00	1.2	60.24	41.83	0.69	0.00E+00	1.1
ENSG00000132170	**PPARG**	2.43	15.37	6.34	NS	117.0	2.97	10.43	3.51	NS	107.0
ENSG00000138032	**PPM1B**	8.70	38.39	4.41	NS	0.4	7.09	35.00	4.94	NS	0.5
ENSG00000158615	**PPP1R15B**	9.63	109.04	11.32	NS	2.5	11.14	96.27	8.64	NS	2.2
ENSG00000162976	**PQLC3**	7.77	27.14	3.49	NS	1.7	4.49	18.11	4.03	NS	1.4
ENSG00000165672	**PRDX3**	94.31	133.25	1.41	4.00E-04	0.7	99.03	194.23	1.96	2.20E-03	0.9
ENSG00000138073	**PREB**	18.31	68.69	3.75	NS	1.1	29.00	48.99	1.69	NS	1.1
ENSG00000132300	**PTCD3**	8.35	23.10	2.76	3.30E-03	0.5	9.63	30.69	3.19	NS	0.7
ENSG00000050628	**PTGER3**	0.04	0.12	3.27	NS	0.3	0.02	0.30	15.63	NS	1.1
ENSG00000177469	**PTRF**	9.59	229.79	23.97	5.00E-04	5.0	4.03	94.28	23.38	NS	2.7
ENSG00000151552	**QDPR**	69.50	34.80	0.50	6.55E-06	2.1	70.61	39.33	0.56	0.00E+00	2.0
ENSG00000109113	**RAB34**	1.67	92.76	55.40	9.00E-06	0.7	0.88	84.34	96.03	0.00E+00	0.8
ENSG00000129625	**REEP5**	41.53	112.57	2.71	4.40E-03	2.3	39.75	89.98	2.26	1.40E-03	1.9

ENSG00000115255	**REEP6**	151.23	3.54	0.02	6.89E-07	0.3	179.89	23.17	0.13	0.00E+00	1.8
ENSG00000042445	**RETSAT**	49.45	20.76	0.42	4.92E-06	1.8	55.79	39.89	0.71	1.00E-04	3.1
ENSG00000101782	**RIOK3**	19.84	111.08	5.60	NS	8.0	18.32	94.95	5.18	NS	6.7
ENSG00000123091	**RNF11**	18.93	84.41	4.46	NS	1.7	18.99	128.74	6.78	NS	2.2
ENSG00000124782	**RREB1**	5.42	17.97	3.32	NS	3.2	5.57	16.69	3.00	NS	2.4
ENSG00000133318	**RTN3**	19.01	84.75	4.46	NS	0.7	18.37	114.46	6.23	NS	1.0
ENSG00000100347	**SAMM50**	6.40	24.56	3.84	NS	0.8	7.21	29.57	4.10	NS	0.9
ENSG00000073060	**SCARB1**	50.16	1.62	0.03	4.87E-08	0.1	53.88	23.34	0.43	7.00E-04	1.1
ENSG00000116171	**SCP2**	129.48	21.53	0.17	1.41E-06	1.7	122.10	26.00	0.21	2.51E-06	2.1
ENSG00000117118	**SDHB**	65.44	42.91	0.66	5.48E-06	0.8	69.59	60.35	0.87	1.00E-04	1.1
ENSG00000143252	**SDHC**	11.70	20.81	1.78	7.00E-04	1.1	11.65	22.88	1.96	1.30E-03	1.3
ENSG00000168497	**SDPR**	5.23	0.15	0.03	1.00E-04	3.6	1.80	0.91	0.51	1.20E-03	21.9
ENSG00000173638	**SLC19A1**	2.55	3.43	1.34	4.30E-03	0.3	3.79	5.01	1.32	NS	0.5
ENSG00000105281	**SLC1A5**	1.37	640.33	467.30	4.81E-06	3.2	1.02	449.17	440.21	0.00E+00	1.8
ENSG00000100075	**SLC25A1**	76.35	69.39	0.91	1.00E-04	0.6	108.64	145.24	1.34	3.70E-03	1.1
ENSG00000183048	**SLC25A10**	61.47	7.87	0.13	3.27E-06	0.3	81.60	14.07	0.17	0.00E+00	0.6
ENSG00000130304	**SLC27A1**	3.63	6.05	1.66	NS	2.5	2.86	3.44	1.20	3.60E-03	1.1
ENSG00000138074	**SLC5A6**	9.08	200.55	22.09	3.00E-04	1.7	13.61	93.77	6.89	NS	1.1
ENSG00000173267	**SNCG**	1.25	2.94	2.35	NS	0.5	0.79	2.56	3.26	NS	0.3
ENSG00000142168	**SOD1**	416.76	112.95	0.27	4.32E-06	0.8	569.21	148.84	0.26	9.53E-06	0.9
ENSG00000095637	**SORBS1**	9.77	0.62	0.06	8.91E-08	0.0	10.03	4.84	0.48	2.00E-04	0.3
ENSG00000152583	**SPARCL1**	6.31	0.35	0.06	0.00E+00	0.2	2.74	3.22	1.18	6.30E-03	2.1
ENSG00000137767	**SQRDL**	20.72	7.73	0.37	3.70E-06	31.0	20.78	5.78	0.28	1.00E-04	39.0
ENSG00000123096	**SSPN**	0.36	2.62	7.31	NS	3.9	0.16	1.08	6.64	NS	1.7
ENSG00000126561	**STAT5A**	3.52	6.41	1.82	6.00E-04	1.3	2.87	3.64	1.27	1.00E-03	1.0
ENSG00000148175	**STOM**	132.96	119.84	0.90	4.00E-04	3.0	77.20	136.79	1.77	6.00E-04	4.6
ENSG00000163541	**SUCLG1**	30.55	27.81	0.91	0.00E+00	1.4	25.33	39.15	1.55	3.00E-04	1.9
ENSG00000196502	**SULT1A1**	31.27	1.17	0.04	3.53E-07	1.2	27.11	2.78	0.10	4.83E-06	1.5
ENSG00000177156	**TALDO1**	39.47	145.33	3.68	NS	0.8	42.14	178.58	4.24	NS	1.1
ENSG00000136560	**TANK**	7.04	21.26	3.02	NS	2.9	6.52	20.58	3.15	NS	2.6
ENSG00000163931	**TKT**	7.33	221.55	30.22	8.42E-06	0.9	8.19	206.24	25.17	1.00E-04	0.7
ENSG00000141232	**TOB1**	39.19	45.55	1.16	1.00E-04	2.2	45.59	52.14	1.14	1.00E-04	2.3
ENSG00000128311	**TST**	207.39	23.39	0.11	7.67E-07	1.1	255.45	25.04	0.10	1.64E-06	1.2

ENSG00000150991	**UBC**	226.49	1280.90	5.66	NS	2.4	251.97	1330.75	5.28	NS	2.4
ENSG00000135018	**UBQLN1**	11.52	87.70	7.61	NS	1.2	13.05	90.82	6.96	NS	1.2
ENSG00000130717	**UCK1**	16.36	39.05	2.39	2.00E-03	1.0	19.24	45.88	2.39	5.40E-03	1.2
ENSG00000175567	**UCP2**	4.74	4.24	0.90	NS	0.2	1.28	17.62	13.74	NS	0.4
ENSG00000184076	**UQCR10**	73.22	109.81	1.50	7.00E-04	0.8	84.69	171.41	2.02	8.80E-03	1.1
ENSG00000127540	**UQCR11**	52.54	48.40	0.92	7.00E-04	0.7	60.85	75.45	1.24	4.90E-03	1.1
ENSG00000010256	**UQCRC1**	73.65	157.49	2.14	2.50E-03	1.0	85.49	138.73	1.62	1.50E-03	0.9
ENSG00000164405	**UQCRQ**	150.51	46.07	0.31	1.00E-04	0.6	149.63	75.66	0.51	3.00E-04	1.0
ENSG00000173511	**VEGFB**	15.79	57.19	3.62	NS	2.0	15.36	78.50	5.11	NS	3.1
ENSG00000170027	**YWHAG**	19.25	269.63	14.01	5.60E-03	0.8	22.10	290.78	13.16	8.10E-03	0.9

Hallmark Cholesterol Homeostasis

ENSG00000107331	**ABCA2**	10.88	1.14	0.10	3.72E-07	0.2	9.70	5.46	0.56	3.00E-04	0.9
ENSG00000120437	**ACAT2**	28.37	24.15	0.85	3.00E-04	0.2	50.99	45.02	0.88	5.40E-03	0.3
ENSG00000131069	**ACSS2**	43.22	18.11	0.42	1.00E-04	1.3	61.85	18.34	0.30	1.00E-04	0.9
ENSG00000184009	**ACTG1**	218.07	4348.52	19.94	1.00E-04	2.0	212.39	3291.27	15.50	9.00E-04	1.4
ENSG00000198099	**ADH4**	1838.30	0.06	0.00	7.80E-08	0.5	2589.75	0.45	0.00	2.47E-09	1.0
ENSG00000170017	**ALCAM**	14.36	16.39	1.14	1.00E-04	0.5	12.93	67.72	5.24	NS	2.9
ENSG00000109107	**ALDOC**	47.39	3.17	0.07	8.69E-06	0.1	33.94	5.58	0.16	0.00E+00	0.1
ENSG00000163297	**ANTXR2**	4.91	35.30	7.19	NS	11.0	4.41	14.97	3.39	NS	6.1
ENSG00000104537	**ANXA13**	9.08	0.00	0.00	2.00E-04	#DIV/0!	3.50	0.01	0.00	3.00E-04	#DIV/0!
ENSG00000164111	**ANXA5**	25.16	769.00	30.57	1.00E-04	3.2	13.22	614.27	46.46	3.00E-04	2.9
ENSG00000162772	**ATF3**	0.90	145.28	161.34	4.28E-06	14.4	1.06	146.83	138.73	6.21E-06	16.5
ENSG00000169136	**ATF5**	462.12	54.49	0.12	3.47E-06	3.5	435.87	78.94	0.18	8.16E-06	3.2
ENSG00000204842	**ATXN2**	3.50	17.56	5.02	NS	2.1	3.52	18.28	5.19	NS	2.1
ENSG00000166148	**AVPR1A**	3.64	0.14	0.04	0.00E+00	0.7	2.23	4.89	2.19	NS	22.5
ENSG00000160200	**CBS**	36.94	46.42	1.26	6.00E-04	0.4	79.97	72.93	0.91	3.10E-03	0.7
ENSG00000010278	**CD9**	6.62	228.24	34.50	1.00E-04	2.3	5.29	97.34	18.39	NS	0.9
ENSG00000110721	**CHKA**	27.97	72.99	2.61	NS	1.4	27.72	74.21	2.68	NS	1.3
ENSG00000120885	**CLU**	859.16	23.62	0.03	1.77E-08	0.2	749.50	52.36	0.07	2.38E-07	0.7
ENSG00000137449	**CPEB2**	5.43	16.06	2.96	NS	17.1	5.93	16.46	2.77	NS	23.8
ENSG00000168036	**CTNNB1**	32.31	339.00	10.49	NS	2.1	28.64	314.71	10.99	NS	1.4
ENSG00000161921	**CXCL16**	12.05	11.82	0.98	6.90E-03	2.8	5.33	10.45	1.96	4.30E-03	7.6
ENSG00000001630	**CYP51A1**	56.30	42.22	0.75	6.00E-04	0.5	76.79	59.42	0.77	2.00E-03	0.6

ENSG00000172893	**DHCR7**	54.07	9.52	0.18	0.00E+00	0.1	57.04	35.68	0.63	1.10E-03	0.4
ENSG00000147155	**EBP**	147.24	27.70	0.19	1.00E-04	0.4	162.11	43.59	0.27	3.00E-04	0.6
ENSG00000104823	**ECH1**	172.35	52.06	0.30	4.18E-06	0.7	215.23	70.43	0.33	0.00E+00	0.8
ENSG00000116285	**ERRFI1**	219.19	654.42	2.99	8.00E-03	9.5	299.96	673.86	2.25	8.70E-03	9.1
ENSG00000105755	**ETHE1**	22.33	43.94	1.97	7.60E-03	2.6	18.11	41.26	2.28	5.10E-03	2.7
ENSG00000164687	**FABP5**	0.25	13.37	54.14	4.00E-04	0.1	0.32	29.89	93.00	1.00E-04	0.3
ENSG00000134824	**FADS2**	35.62	10.14	0.28	1.30E-03	0.1	29.15	45.89	1.57	NS	0.2
ENSG00000135842	**FAM129A**	0.58	66.41	114.44	6.00E-04	2.9	0.17	40.68	243.72	1.60E-03	2.0
ENSG00000169710	**FASN**	47.98	71.52	1.49	9.80E-03	0.2	132.73	113.10	0.85	NS	0.3
ENSG00000116663	**FBXO6**	8.16	18.18	2.23	4.70E-03	32.5	6.93	23.87	3.44	NS	22.2
ENSG00000079459	**FDFT1**	13.70	93.15	6.80	NS	0.5	28.64	83.14	2.90	NS	0.4
ENSG00000160752	**FDPS**	123.69	50.11	0.41	3.10E-03	0.3	109.04	71.74	0.66	7.20E-03	0.4
ENSG00000178445	**GLDC**	49.51	28.24	0.57	0.00E+00	0.3	36.34	69.71	1.92	2.10E-03	0.8
ENSG00000127955	**GNAI1**	6.59	17.41	2.64	4.80E-03	1.5	8.17	20.29	2.49	NS	1.6
ENSG00000164294	**GPX8**	0.44	90.42	204.64	0.00E+00	2.6	0.23	92.80	400.20	0.00E+00	2.1
ENSG00000213366	**GSTM2**	33.51	0.99	0.03	2.70E-03	0.4	5.89	2.63	0.45	9.40E-03	1.0
ENSG00000169919	**GUSB**	48.77	41.66	0.85	0.00E+00	1.6	39.45	44.71	1.13	1.00E-04	1.6
ENSG00000113161	**HMGCR**	6.57	47.49	7.23	NS	0.3	11.27	63.72	5.66	NS	0.4
ENSG00000112972	**HMGCS1**	27.60	39.38	1.43	1.50E-03	0.2	95.73	68.95	0.72	7.00E-03	0.2
ENSG00000132196	**HSD17B7**	5.40	5.18	0.96	2.00E-04	0.5	10.19	5.09	0.50	3.00E-04	0.8
ENSG00000067064	**IDI1**	28.99	28.70	0.99	2.00E-04	0.3	58.11	47.73	0.82	2.20E-03	0.5
ENSG00000101384	**JAG1**	1.63	22.79	14.02	NS	1.8	0.98	33.54	34.14	5.10E-03	2.8
ENSG00000130164	**LDLR**	2.44	76.32	31.33	NS	1.8	5.97	30.68	5.14	NS	0.7
ENSG00000131981	**LGALS3**	4.18	85.85	20.56	1.40E-03	19.9	2.04	97.56	47.71	5.90E-03	17.7
ENSG00000100600	**LGMN**	22.16	64.88	2.93	NS	1.7	19.00	74.32	3.91	NS	1.3
ENSG00000175445	**LPL**	0.08	44.52	532.69	7.00E-04	6.4	0.00	24.00	5041.67	3.30E-03	2.3
ENSG00000160285	**LSS**	46.38	23.43	0.51	1.00E-03	0.3	53.37	42.15	0.79	6.00E-03	0.5
ENSG00000167508	**MVD**	5.20	11.33	2.18	NS	0.4	17.98	23.85	1.33	NS	0.5
ENSG00000110921	**MVK**	8.59	6.45	0.75	3.00E-04	1.1	15.07	6.85	0.45	5.00E-04	1.0
ENSG00000165030	**NFIL3**	12.40	104.97	8.46	NS	6.7	7.14	84.18	11.79	NS	5.0
ENSG00000147383	**NSDHL**	13.39	31.03	2.32	6.20E-03	0.6	24.15	43.09	1.78	NS	0.8
ENSG00000185813	**PCYT2**	35.69	15.16	0.42	1.00E-04	1.1	43.05	43.36	1.01	4.80E-03	3.1
ENSG00000067992	**PDK3**	1.05	5.91	5.62	NS	0.4	0.68	7.67	11.27	NS	0.5

ENSG00000011422	**PLAUR**	0.31	45.46	148.18	1.00E-04	6.4	0.08	10.44	123.99	NS	1.0
ENSG00000188313	**PLSCR1**	3.74	67.71	18.10	NS	3.9	3.26	50.38	15.44	NS	2.7
ENSG00000163344	**PMVK**	45.55	28.92	0.63	0.00E+00	0.7	51.46	44.98	0.87	2.00E-04	1.3
ENSG00000146278	**PNRC1**	28.12	33.02	1.17	2.00E-04	2.2	19.53	29.68	1.52	4.00E-04	2.3
ENSG00000132170	**PPARG**	2.43	15.37	6.34	NS	117.0	2.97	10.43	3.51	NS	107.0
ENSG00000163191	**S100A11**	24.52	3158.97	128.81	9.71E-06	12.0	12.42	1898.00	152.78	1.00E-04	7.0
ENSG00000099194	**SCD**	586.39	21.85	0.04	0.00E+00	0.0	361.18	168.63	0.47	1.00E-03	0.2
ENSG00000012171	**SEMA3B**	2.02	1.28	0.63	NS	0.7	1.78	7.15	4.02	NS	4.2
ENSG00000104549	**SQLE**	2.06	35.04	16.97	NS	0.3	10.33	42.15	4.08	NS	0.3
ENSG00000198911	**SREBF2**	15.99	64.67	4.04	NS	0.6	27.88	117.29	4.21	NS	1.1
ENSG00000164211	**STARD4**	5.14	21.75	4.23	NS	0.5	9.08	32.58	3.59	NS	0.7
ENSG00000162236	**STX5**	15.87	86.90	5.48	NS	3.8	21.64	80.21	3.71	NS	3.0
ENSG00000149809	**TM7SF2**	41.70	6.32	0.15	0.00E+00	0.4	54.64	16.98	0.31	1.00E-04	0.6
ENSG00000109084	**TMEM97**	39.46	195.66	4.96	NS	1.5	44.19	148.19	3.35	NS	1.0
ENSG00000006327	**TNFRSF12A**	1.48	284.84	192.49	2.44E-06	4.7	0.95	228.89	241.42	4.59E-06	4.2
ENSG00000164938	**TP53INP1**	42.38	65.36	1.54	6.20E-03	6.2	31.31	117.26	3.75	NS	12.7
ENSG00000101255	**TRIB3**	8.67	118.04	13.62	6.00E-04	12.4	7.53	98.69	13.10	4.60E-03	6.2

Hallmark Glycolysis

ENSG00000115657	**ABCB6**	9.48	17.33	1.83	1.10E-03	1.0	0.68	7.67	11.27	NS	0.5
ENSG00000170425	**ADORA2B**	0.03	9.37	326.20	5.00E-04	2.9	1.46	48.13	33.00	4.30E-03	3.5
ENSG00000162688	**AGL**	14.73	15.42	1.05	1.30E-03	0.9	13.53	41.65	3.08	NS	3.2
ENSG00000188157	**AGRN**	8.71	73.23	8.41	NS	1.6	0.30	6.19	20.29	3.80E-03	0.5
ENSG00000147853	**AK3**	61.90	70.65	1.14	1.00E-04	2.1	9.07	10.03	1.11	6.00E-04	6.7
ENSG00000162433	**AK4**	32.04	4.41	0.14	2.17E-07	0.1	9.26	40.09	4.33	NS	2.0
ENSG00000117448	**AKR1A1**	122.26	63.01	0.52	1.00E-04	0.5	89.10	72.28	0.81	2.00E-04	0.8
ENSG00000164904	**ALDH7A1**	43.55	24.47	0.56	7.35E-06	0.4	6.54	3.58	0.55	0.00E+00	4.5
ENSG00000143149	**ALDH9A1**	80.16	75.13	0.94	0.00E+00	0.9	12.72	14.37	1.13	0.00E+00	2.7
ENSG00000149925	**ALDOA**	15.46	301.59	19.51	0.00E+00	1.0	0.65	17.41	26.90	2.00E-04	3.5
ENSG00000136872	**ALDOB**	4282.19	0.16	0.00	1.72E-09	#DIV/0!	3707.62	0.99	0.00	1.42E-09	1.5
ENSG00000033011	**ALG1**	3.19	11.20	3.51	NS	0.6	23.83	87.79	3.68	NS	0.6
ENSG00000214274	**ANG**	548.51	2.55	0.00	7.74E-09	0.9	710.63	7.80	0.01	6.41E-08	3.0
ENSG00000167772	**ANGPTL4**	9.42	83.96	8.91	NS	72.2	11.65	22.88	1.96	NS	1.3
ENSG00000163516	**ANKZF1**	13.26	33.88	2.56	NS	1.6	4.32	6.29	1.46	NS	0.9

ENSG00000128989	**ARPP19**	8.43	148.54	17.62	1.50E-03	1.2	2.47	78.78	31.91	1.90E-03	10.6
ENSG00000117407	**ARTN**	0.05	0.66	14.26	NS	0.1	0.09	0.92	10.71	NS	0.2
ENSG00000087586	**AURKA**	0.61	26.49	43.51	1.00E-04	0.4	28.23	514.46	18.23	3.00E-04	19.2
ENSG00000176022	**B3GALT6**	5.12	21.19	4.14	NS	1.6	72.17	419.93	5.82	NS	85.5
ENSG00000109956	**B3GAT1**	1.21	0.40	0.33	0.00E+00	0.0	124.93	408.41	3.27	NS	0.7
ENSG00000149541	**B3GAT3**	12.63	32.36	2.56	NS	1.8	3.70	16.94	4.58	NS	0.8
ENSG00000179913	**B3GNT3**	0.52	3.61	6.95	NS	1.6	1.45	11.87	8.17	NS	0.5
ENSG00000086062	**B4GALT1**	25.34	149.78	5.91	NS	8.5	7.81	49.04	6.28	NS	3.0
ENSG00000117411	**B4GALT2**	11.42	44.93	3.94	NS	1.1	17.15	25.00	1.46	NS	1.2
ENSG00000121578	**B4GALT4**	3.43	41.85	12.20	3.00E-04	1.8	0.39	4.42	11.23	1.00E-03	0.4
ENSG00000027847	**B4GALT7**	4.29	12.41	2.89	NS	1.6	17.54	24.04	1.37	NS	1.5
ENSG00000100290	**BIK**	0.33	4.93	14.80	NS	1.7	0.02	6.58	300.85	NS	2.6
ENSG00000162813	**BPNT1**	8.38	17.84	2.13	1.30E-03	1.3	29.96	47.67	1.59	NS	3.8
ENSG00000196557	**CACNA1H**	18.99	1.00	0.05	1.78E-06	0.1	36.88	3.85	0.10	0.00E+00	0.2
ENSG00000149260	**CAPN5**	22.69	10.48	0.46	3.40E-06	0.6	28.65	15.06	0.53	0.00E+00	0.8
ENSG00000138794	**CASP6**	9.96	35.10	3.52	NS	1.5	5.18	26.34	5.09	NS	1.7
ENSG00000026508	**CD44**	1.45	63.99	44.23	7.80E-03	11.3	1.29	25.34	19.68	NS	14.8
ENSG00000170312	**CDK1**	0.31	52.56	169.24	2.00E-04	0.7	0.07	56.15	755.85	2.00E-04	0.7
ENSG00000115163	**CENPA**	0.11	16.29	147.01	0.00E+00	0.7	0.03	16.79	531.95	1.00E-04	0.9
ENSG00000123989	**CHPF**	22.19	89.89	4.05	NS	1.6	6.66	20.21	3.03	NS	1.6
ENSG00000033100	**CHPF2**	7.48	34.87	4.66	NS	1.7	4.77	35.72	7.49	NS	1.5
ENSG00000175264	**CHST1**	0.52	5.47	10.53	2.60E-03	3.8	17.08	42.83	2.51	NS	20.0
ENSG00000136213	**CHST12**	2.44	9.09	3.72	NS	1.0	1.43	14.17	9.89	NS	0.6
ENSG00000175040	**CHST2**	0.42	2.47	5.95	NS	0.2	7.77	175.45	22.59	NS	1.4
ENSG00000140835	**CHST4**	1.08	0.02	0.01	7.28E-07	0.0	0.75	0.03	0.05	5.67E-06	0.0
ENSG00000183196	**CHST6**	0.00	0.35	120.79	0.00E+00	0.0	0.00	0.49	200.44	0.00E+00	0.0
ENSG00000164442	**CITED2**	23.57	191.12	8.11	NS	3.0	0.15	1.08	7.46	NS	0.4
ENSG00000165215	**CLDN3**	16.86	3.50	0.21	0.00E+00	0.2	569.21	148.84	0.26	1.00E-04	0.9
ENSG00000213937	**CLDN9**	6.28	5.00	0.80	NS	1.6	115.24	81.65	0.71	NS	0.7
ENSG00000128973	**CLN6**	5.64	44.32	7.86	NS	0.9	8.19	37.82	4.62	NS	1.3
ENSG00000135775	**COG2**	1.70	9.24	5.44	NS	0.8	129.51	491.81	3.80	NS	1.6
ENSG00000130635	**COL5A1**	1.95	505.09	258.91	1.07E-06	52.7	0.17	72.40	430.69	2.20E-03	4.1
ENSG00000184432	**COPB2**	10.89	79.60	7.31	NS	1.4	1.79	10.81	6.05	NS	0.6

ENSG00000116761	**CTH**	23.60	60.01	2.54	7.00E-03	2.2	6.00	27.33	4.55	NS	2.3
ENSG00000121966	**CXCR4**	1.51	1.22	0.80	6.00E-04	0.5	3.14	45.06	14.34	NS	2.8
ENSG00000166347	**CYB5A**	99.96	6.56	0.07	1.59E-07	0.4	117.69	16.00	0.14	3.65E-06	0.9
ENSG00000011465	**DCN**	16.72	2.79	0.17	2.00E-04	9.4	0.69	0.25	0.36	1.00E-04	0.4
ENSG00000168209	**DDIT4**	28.76	911.04	31.68	1.00E-04	47.5	4.85	43.65	9.00	1.90E-03	5.5
ENSG00000024526	**DEPDC1**	0.06	12.15	195.82	0.00E+00	0.5	0.04	9.03	232.48	2.00E-04	0.3
ENSG00000091140	**DLD**	18.16	68.13	3.75	NS	1.3	3.99	16.31	4.09	NS	1.1
ENSG00000151640	**DPYSL4**	0.19	4.98	25.90	1.70E-03	0.2	0.05	28.55	620.96	4.00E-04	1.3
ENSG00000134755	**DSC2**	1.10	58.68	53.19	2.00E-04	3.9	1.43	103.91	72.46	3.00E-04	2.3
ENSG00000122882	**ECD**	5.01	34.41	6.87	NS	0.7	2.09	23.84	11.41	NS	2.8
ENSG00000143590	**EFNA3**	0.27	1.73	6.43	NS	0.2	23.01	227.23	9.88	NS	3.5
ENSG00000146648	**EGFR**	13.47	60.53	4.49	NS	24.7	2.53	6.51	2.57	NS	1.5
ENSG00000129521	**EGLN3**	0.16	0.30	1.90	NS	0.0	0.09	11.12	130.76	NS	0.9
ENSG00000163435	**ELF3**	3.81	6.76	1.77	NS	1.0	13.16	57.68	4.38	NS	2.0
ENSG00000074800	**ENO1**	94.71	811.54	8.57	NS	0.6	13.24	121.50	9.18	6.40E-03	2.4
ENSG00000111674	**ENO2**	1.65	5.52	3.34	NS	0.1	0.64	64.90	101.05	NS	1.1
ENSG00000182197	**EXT1**	7.16	59.75	8.34	NS	4.8	9.22	115.50	12.53	NS	2.4
ENSG00000151348	**EXT2**	7.28	75.44	10.36	NS	2.1	7.30	42.39	5.81	NS	0.9
ENSG00000114023	**FAM162A**	25.72	8.74	0.34	0.00E+00	0.4	28.90	15.34	0.53	1.00E-04	0.6
ENSG00000004478	**FKBP4**	13.76	110.85	8.06	NS	0.8	0.46	1.17	2.53	NS	1.8
ENSG00000033170	**FUT8**	0.37	105.12	282.81	1.00E-04	11.3	0.32	470.42	1491.26	3.00E-04	4.7
ENSG00000160211	**G6PD**	1.69	84.74	50.09	6.05E-06	2.0	0.15	3.70	25.14	7.81E-06	1.1
ENSG00000128242	**GAL3ST1**	1.55	0.31	0.20	1.20E-03	0.1	125.10	176.61	1.41	NS	0.5
ENSG00000117308	**GALE**	19.78	62.24	3.15	NS	5.3	0.81	2.82	3.49	NS	0.2
ENSG00000108479	**GALK1**	59.61	9.81	0.16	1.91E-06	0.8	36.26	9.69	0.27	3.24E-06	1.1
ENSG00000156958	**GALK2**	2.45	7.85	3.20	NS	1.8	32.26	47.59	1.48	8.90E-03	2.0
ENSG00000001084	**GCLC**	22.08	18.19	0.82	0.00E+00	0.8	11.50	28.10	2.44	1.40E-03	5.4
ENSG00000198380	**GFPT1**	3.95	136.85	34.65	1.60E-03	2.7	1.00	31.13	31.14	1.60E-03	3.5
ENSG00000138604	**GLCE**	4.52	7.19	1.59	5.00E-03	0.8	43.91	101.34	2.31	2.90E-03	1.9
ENSG00000173221	**GLRX**	13.79	4.79	0.35	3.92E-06	1.0	26.65	42.87	1.61	3.10E-03	1.8
ENSG00000144591	**GMPPA**	9.72	21.56	2.22	NS	1.5	55.54	98.87	1.78	NS	3.6
ENSG00000173540	**GMPPB**	3.50	17.12	4.89	NS	1.3	31.13	102.58	3.29	NS	0.7
ENSG00000159921	**GNE**	45.35	10.86	0.24	5.03E-06	1.0	8.53	4.29	0.50	8.74E-06	104.9

ENSG00000113552	**GNPDA1**	5.31	127.08	23.92	0.00E+00	1.6	3.90	174.07	44.67	0.00E+00	2.2
ENSG00000120053	**GOT1**	100.17	156.18	1.56	3.00E-04	2.6	18.86	16.79	0.89	2.00E-04	1.1
ENSG00000125166	**GOT2**	108.15	88.16	0.82	0.00E+00	0.5	162.47	108.38	0.67	1.00E-04	0.5
ENSG00000063660	**GPC1**	7.18	39.18	5.46	NS	2.5	8.48	46.42	5.47	NS	1.1
ENSG00000147257	**GPC3**	1.68	36.42	21.62	4.20E-03	0.3	0.34	142.72	424.23	2.00E-04	1.9
ENSG00000076716	**GPC4**	0.56	45.10	80.37	0.00E+00	0.2	0.30	49.05	163.89	2.00E-04	2.9
ENSG00000138271	**GPR87**	0.08	97.26	1281.04	2.97E-06	187.5	0.01	87.77	6766.40	4.16E-06	430.0
ENSG00000169919	**GUSB**	48.77	41.66	0.85	0.00E+00	1.6	12.87	38.45	2.99	1.00E-04	2.2
ENSG00000104812	**GYS1**	8.59	30.05	3.50	NS	1.0	21.32	81.13	3.81	NS	1.5
ENSG00000111713	**GYS2**	99.32	0.10	0.00	8.55E-09	0.6	68.29	0.29	0.00	7.81E-08	2.1
ENSG00000143575	**HAX1**	29.75	128.37	4.31	NS	2.6	13.06	50.71	3.88	NS	2.6
ENSG00000115677	**HDLBP**	43.63	174.45	4.00	NS	1.5	26.53	172.82	6.51	NS	0.8
ENSG00000159399	**HK2**	0.12	64.11	513.67	4.00E-04	1.1	0.09	70.20	755.89	4.00E-04	1.2
ENSG00000072571	**HMMR**	0.35	17.59	50.63	1.00E-04	0.3	0.87	61.92	71.32	1.00E-03	3.3
ENSG00000152413	**HOMER1**	0.18	12.31	67.43	7.00E-04	0.4	0.05	14.72	289.58	6.00E-04	0.4
ENSG00000153936	**HS2ST1**	5.17	21.83	4.23	NS	0.9	0.88	16.83	19.09	NS	5.3
ENSG00000171004	**HS6ST2**	0.00	3.08	623.31	5.63E-06	0.4	0.02	12.67	751.50	2.56E-06	2.0
ENSG00000044574	**HSPA5**	62.71	682.50	10.88	NS	1.4	2.78	34.02	12.24	NS	2.4
ENSG00000138413	**IDH1**	130.12	76.64	0.59	8.11E-06	0.3	8.75	18.05	2.06	1.20E-03	2.0
ENSG00000127415	**IDUA**	6.63	2.73	0.41	NS	3.1	96.66	97.54	1.01	NS	0.4
ENSG00000137331	**IER3**	1.60	33.86	21.13	4.00E-04	4.1	20.30	160.26	7.89	2.10E-03	25.7
ENSG00000146674	**IGFBP3**	105.99	669.73	6.32	NS	110.2	2.10	11.68	5.56	NS	1.0
ENSG00000131724	**IL13RA1**	55.02	64.87	1.18	1.00E-04	6.8	73.71	101.25	1.37	2.00E-04	1.1
ENSG00000185950	**IRS2**	2.87	67.71	23.56	1.00E-03	6.9	1.43	14.54	10.16	NS	1.3
ENSG00000172183	**ISG20**	0.48	2.20	4.54	NS	3.1	0.58	2.64	4.55	NS	0.8
ENSG00000100196	**KDELR3**	8.06	12.74	1.58	NS	1.1	118.56	1254.64	10.58	NS	0.9
ENSG00000112984	**KIF20A**	0.13	18.64	143.87	8.00E-04	0.3	0.05	13.87	292.36	2.30E-03	0.2
ENSG00000068796	**KIF2A**	0.96	58.43	61.10	5.00E-04	1.1	1.29	72.75	56.25	5.00E-04	6.9
ENSG00000115850	**LCT**	0.00	0.23	60.90	2.55E-06	7.4	0.00	0.11	35.71	4.00E-04	7.6
ENSG00000134333	**LDHA**	76.76	111.88	1.46	2.00E-04	0.5	46.97	61.84	1.32	2.00E-04	5.6
ENSG00000166796	**LDHC**	1.61	0.41	0.25	NS	0.4	1.29	0.22	0.17	NS	0.2
ENSG00000107902	**LHPP**	17.24	3.73	0.22	2.19E-06	0.4	18.64	6.35	0.34	0.00E+00	0.5
ENSG00000143355	**LHX9**	0.11	0.00	0.00	0.00E+00	0.0	0.04	0.67	18.56	NS	16.1

ENSG00000014641	**MDH1**	29.38	72.08	2.45	2.00E-03	0.5	5.99	18.92	3.16	NS	0.8
ENSG00000146701	**MDH2**	50.71	106.63	2.10	7.00E-04	0.7	49.98	122.38	2.45	2.80E-03	0.8
ENSG00000065833	**ME1**	3.30	38.69	11.72	2.50E-03	5.0	4.34	186.60	43.04	3.00E-04	11.2
ENSG00000082212	**ME2**	1.83	14.31	7.83	NS	0.5	1.93	65.80	34.14	NS	2.2
ENSG00000008838	**MED24**	8.36	38.11	4.56	NS	0.9	125.79	616.12	4.90	NS	1.1
ENSG00000153208	**MERTK**	3.93	40.75	10.38	NS	6.6	23.45	170.46	7.27	NS	9.8
ENSG00000105976	**MET**	14.53	52.96	3.64	NS	5.4	78.77	130.61	1.66	NS	0.6
ENSG00000240972	**MIF**	50.35	111.56	2.22	5.30E-03	0.5	16.55	10.38	0.63	NS	5.5
ENSG00000100253	**MIOX**	0.29	3.40	11.91	NS	12.1	27.64	135.98	4.92	NS	1.0
ENSG00000178802	**MPI**	3.62	4.83	1.34	1.00E-04	0.7	42.62	46.01	1.08	3.00E-04	6.2
ENSG00000119950	**MXI1**	10.51	15.03	1.43	4.00E-04	2.7	24.15	43.09	1.78	NS	0.8
ENSG00000170191	**NANP**	1.58	12.18	7.69	NS	1.2	3.77	55.35	14.68	NS	4.4
ENSG00000132780	**NASP**	12.80	70.33	5.49	NS	0.2	1.74	13.59	7.81	NS	1.8
ENSG00000164100	**NDST3**	0.31	2.96	9.44	NS	3.9	0.04	2.81	67.49	NS	4.5
ENSG00000160194	**NDUFV3**	9.08	15.37	1.69	2.20E-03	0.9	8.47	18.30	2.16	6.90E-03	1.0
ENSG00000140939	**NOL3**	11.74	12.02	1.02	NS	11.2	39.45	44.71	1.13	NS	1.6
ENSG00000147383	**NSDHL**	13.39	31.03	2.32	6.20E-03	0.6	17.54	35.76	2.04	NS	4.9
ENSG00000135318	**NT5E**	19.79	78.19	3.95	NS	38.2	48.96	37.69	0.77	3.30E-03	0.5
ENSG00000122884	**P4HA1**	3.62	40.60	11.23	NS	1.6	12.04	262.38	21.80	NS	0.9
ENSG00000072682	**P4HA2**	1.41	20.48	14.55	1.00E-04	9.1	2.85	77.75	27.29	3.00E-04	3.0
ENSG00000145730	**PAM**	1.80	54.54	30.36	3.90E-03	3.2	0.20	12.08	59.97	4.40E-03	0.2
ENSG00000157212	**PAXIP1**	1.54	10.27	6.65	NS	0.5	68.19	326.46	4.79	NS	0.7
ENSG00000173599	**PC**	39.27	3.08	0.08	6.02E-07	0.4	62.21	10.73	0.17	8.61E-06	0.7
ENSG00000067992	**PDK3**	1.05	5.91	5.62	NS	0.4	0.41	1.17	2.82	NS	0.8
ENSG00000158571	**PFKFB1**	45.24	0.57	0.01	9.62E-08	1.6	47.83	0.53	0.01	1.09E-07	1.1
ENSG00000067057	**PFKP**	0.49	203.10	410.48	2.88E-07	3.3	1.17	253.44	216.83	1.08E-06	27.6
ENSG00000171314	**PGAM1**	23.53	129.13	5.49	NS	0.4	20.68	181.29	8.77	NS	0.5
ENSG00000164708	**PGAM2**	2.25	0.04	0.02	5.00E-04	0.0	1.63	0.21	0.13	2.70E-03	0.1
ENSG00000102144	**PGK1**	27.69	164.38	5.94	NS	0.8	2.98	35.91	12.03	NS	0.9
ENSG00000130313	**PGLS**	12.97	69.90	5.39	NS	2.9	2.03	13.59	6.70	NS	2.9
ENSG00000169299	**PGM2**	5.80	102.10	17.60	6.40E-03	4.4	0.13	0.81	6.33	NS	4.8
ENSG00000044446	**PHKA2**	16.85	22.66	1.34	1.00E-04	1.4	2.37	1.93	0.81	4.00E-04	0.3
ENSG00000057294	**PKP2**	5.62	190.88	33.95	1.00E-03	7.5	0.69	46.60	67.36	1.90E-03	0.8

ENSG00000083444	**PLOD1**	32.39	49.67	1.53	3.00E-04	1.5	34.20	62.46	1.83	1.50E-03	1.7
ENSG00000152952	**PLOD2**	6.92	22.57	3.26	NS	1.6	8.54	24.24	2.84	NS	1.9
ENSG00000140650	**PMM2**	4.32	16.67	3.86	NS	0.7	4.77	20.51	4.30	NS	0.9
ENSG00000161980	**POLR3K**	2.96	11.68	3.95	NS	0.3	13.40	92.87	6.93	NS	1.6
ENSG00000143847	**PPFIA4**	0.11	0.05	0.47	7.20E-03	0.0	0.03	0.47	18.20	NS	0.2
ENSG00000196262	**PPIA**	72.46	326.11	4.50	NS	0.7	18.56	31.54	1.70	NS	21.5
ENSG00000104695	**PPP2CB**	15.06	127.15	8.44	NS	2.6	13.20	85.80	6.50	NS	0.3
ENSG00000147224	**PRPS1**	18.35	46.99	2.56	2.20E-03	0.2	11.13	28.08	2.52	2.10E-03	1.9
ENSG00000013275	**PSMC4**	20.25	117.83	5.82	NS	0.9	6.77	42.03	6.21	NS	9.0
ENSG00000100994	**PYGB**	6.35	109.30	17.21	1.00E-04	2.0	0.46	24.63	54.03	1.60E-03	0.3
ENSG00000100504	**PYGL**	51.08	54.29	1.06	1.00E-04	1.6	3.06	6.11	2.00	4.90E-03	0.5
ENSG00000116260	**QSOX1**	6.56	30.55	4.66	NS	1.3	3.78	104.44	27.61	NS	4.0
ENSG00000113643	**RARS**	14.07	92.27	6.56	NS	0.9	3.14	17.71	5.65	NS	0.4
ENSG00000125826	**RBCK1**	30.87	67.97	2.20	NS	2.9	93.50	121.49	1.30	NS	1.9
ENSG00000197713	**RPE**	5.53	48.72	8.82	NS	1.0	5.80	58.70	10.11	NS	1.0
ENSG00000025039	**RRAGD**	2.27	17.86	7.86	NS	1.4	7.45	79.14	10.62	NS	2.3
ENSG00000164105	**SAP30**	1.48	9.71	6.57	NS	0.4	17.92	106.53	5.95	NS	0.9
ENSG00000115884	**SDC1**	253.36	47.37	0.19	2.28E-06	0.7	108.14	34.97	0.32	0.00E+00	0.5
ENSG00000169439	**SDC2**	87.32	12.06	0.14	3.22E-07	0.2	254.85	72.05	0.28	0.00E+00	1.4
ENSG00000162512	**SDC3**	6.00	97.68	16.28	NS	4.7	6.24	219.26	35.13	NS	4.6
ENSG00000143252	**SDHC**	11.70	20.81	1.78	7.00E-04	1.1	21.70	45.68	2.10	1.30E-03	0.3
ENSG00000141526	**SLC16A3**	0.49	45.34	92.28	0.00E+00	67.5	0.27	42.51	155.51	7.50E-03	0.2
ENSG00000183048	**SLC25A10**	61.47	7.87	0.13	3.27E-06	0.3	49.35	12.15	0.25	0.00E+00	1.1
ENSG00000004864	**SLC25A13**	59.11	73.06	1.24	1.00E-04	0.8	4.69	3.20	0.68	5.00E-04	1.2
ENSG00000117620	**SLC35A3**	7.69	14.56	1.89	2.40E-03	1.6	25.44	86.86	3.41	5.20E-03	3.4
ENSG00000137700	**SLC37A4**	55.53	16.26	0.29	1.17E-06	0.7	51.61	17.23	0.33	3.32E-06	0.7
ENSG00000142168	**SOD1**	416.76	112.95	0.27	4.32E-06	0.8	81.60	14.07	0.17	9.53E-06	0.6
ENSG00000125398	**SOX9**	1.89	145.55	77.17	3.76E-06	23.8	4.45	264.45	59.46	0.00E+00	5.2
ENSG00000061656	**SPAG4**	0.89	0.37	0.41	1.00E-04	0.6	21.81	7.26	0.33	1.00E-04	0.7
ENSG00000128039	**SRD5A3**	3.17	7.44	2.35	5.50E-03	0.7	28.50	134.36	4.71	8.10E-03	1.2
ENSG00000159167	**STC1**	0.10	2.02	20.16	8.50E-03	0.0	0.05	1.85	40.88	NS	0.1
ENSG00000113739	**STC2**	0.07	217.72	3214.39	4.68E-09	9.5	0.05	198.62	3647.04	1.16E-08	7.4
ENSG00000117632	**STMN1**	2.46	181.58	73.68	1.32E-06	1.1	2.53	202.66	80.04	2.33E-06	1.0

ENSG00000177156	**TALDO1**	39.47	145.33	3.68	NS	0.8	9.91	47.46	4.79	NS	1.6
ENSG00000160180	**TFF3**	2.38	0.04	0.02	3.00E-04	0.1	0.39	0.04	0.09	2.00E-04	0.1
ENSG00000163235	**TGFA**	1.24	4.09	3.29	NS	1.5	42.14	178.58	4.24	NS	1.1
ENSG00000120708	**TGFBI**	18.48	611.96	33.11	0.00E+00	59.1	4.76	42.46	8.91	NS	0.8
ENSG00000007350	**TKTL1**	0.03	0.00	0.00	NS	0.0	0.01	0.11	19.14	NS	0.6
ENSG00000146242	**TPBG**	0.34	54.12	159.40	2.84E-07	2.6	0.19	22.98	119.42	0.00E+00	74.3
ENSG00000111669	**TPI1**	102.36	308.82	3.02	NS	0.6	18.15	69.14	3.81	NS	1.2
ENSG00000169902	**TPST1**	7.14	17.53	2.46	5.40E-03	1.4	39.31	154.63	3.93	NS	2.6
ENSG00000104522	**TSTA3**	9.76	21.64	2.22	NS	0.9	5.92	13.03	2.20	NS	1.8
ENSG00000136810	**TXN**	129.33	561.16	4.34	NS	1.7	45.51	216.30	4.75	NS	1.7
ENSG00000169764	**UGP2**	114.56	74.60	0.65	0.00E+00	0.3	3.55	6.32	1.78	1.00E-04	0.6
ENSG00000038427	**VCAN**	0.29	9.71	33.72	2.30E-03	0.1	0.46	179.38	388.85	0.00E+00	1.5
ENSG00000112715	**VEGFA**	40.78	45.00	1.10	9.00E-04	6.2	89.44	90.66	1.01	1.20E-03	0.4
ENSG00000147852	**VLDLR**	0.34	46.55	136.97	9.36E-07	2.1	0.25	25.42	102.62	2.89E-06	1.2
ENSG00000015532	**XYLT2**	4.44	16.07	3.62	NS	1.1	14.16	43.33	3.06	NS	1.1
ENSG00000188994	**ZNF292**	1.42	39.45	27.87	2.90E-03	2.5	0.82	57.70	70.52	2.40E-03	3.0

Hallmark Heme Metabolism

ENSG00000115657	**ABCB6**	9.48	17.33	1.83	1.10E-03	1.0	13.53	41.65	3.08	NS	3.2
ENSG00000118777	**ABCG2**	12.05	53.14	4.41	NS	14.1	15.74	85.56	5.44	NS	21.5
ENSG00000102575	**ACP5**	9.39	1.38	0.15	0.00E+00	0.3	4.95	0.59	0.12	7.92E-06	0.1
ENSG00000164398	**ACSL6**	0.93	0.03	0.03	1.96E-06	0.1	0.53	0.39	0.74	2.30E-03	0.6
ENSG00000087274	**ADD1**	11.27	61.89	5.49	NS	2.0	9.50	67.87	7.14	NS	2.1
ENSG00000075340	**ADD2**	0.04	0.66	18.08	3.00E-04	0.0	0.02	4.90	212.45	1.70E-03	0.1
ENSG00000159346	**ADIPOR1**	30.42	136.13	4.48	NS	2.0	27.86	139.74	5.02	NS	2.1
ENSG00000026652	**AGPAT4**	0.23	7.93	33.90	3.00E-04	0.5	0.13	11.27	86.17	1.00E-04	0.7
ENSG00000169877	**AHSP**	0.22	0.00	0.00	4.70E-03	0.0	0.11	0.00	0.00	5.10E-03	0.0
ENSG00000148218	**ALAD**	101.27	15.88	0.16	2.23E-07	1.7	114.46	24.47	0.21	7.73E-07	2.6
ENSG00000144908	**ALDH1L1**	93.84	0.09	0.00	6.07E-08	0.3	92.35	2.02	0.02	1.97E-06	4.7
ENSG00000119711	**ALDH6A1**	70.02	11.16	0.16	0.00E+00	0.8	84.75	30.54	0.36	1.00E-04	1.6
ENSG00000029534	**ANK1**	0.03	0.51	17.27	1.70E-03	0.1	0.02	4.25	275.27	1.90E-03	1.2
ENSG00000165272	**AQP3**	32.40	2.20	0.07	1.82E-07	0.9	33.32	32.82	0.98	2.20E-03	9.3
ENSG00000196914	**ARHGEF12**	8.48	28.81	3.40	NS	2.9	7.81	26.56	3.40	NS	2.5
ENSG00000102931	**ARL2BP**	14.38	115.08	8.00	NS	3.8	13.77	122.44	8.89	NS	4.6

ENSG00000070669	**ASNS**	1.84	315.62	171.91	8.05E-06	4.6	1.98	371.26	187.46	8.70E-06	4.5
ENSG00000101844	**ATG4A**	7.19	13.90	1.93	8.00E-04	3.1	6.51	16.75	2.57	4.20E-03	4.5
ENSG00000033627	**ATP6V0A1**	7.17	25.23	3.52	NS	1.9	6.71	34.21	5.10	NS	2.4
ENSG00000156273	**BACH1**	2.68	39.98	14.90	NS	2.5	3.03	43.91	14.48	NS	2.3
ENSG00000187244	**BCAM**	9.49	33.39	3.52	NS	0.7	6.71	70.46	10.49	NS	1.8
ENSG00000106605	**BLVRA**	2.48	21.85	8.81	NS	0.6	0.87	18.69	21.47	NS	0.6
ENSG00000090013	**BLVRB**	66.94	74.93	1.12	4.00E-04	2.9	106.09	87.90	0.83	7.00E-04	3.0
ENSG00000138756	**BMP2K**	3.31	19.97	6.03	NS	2.1	2.82	24.84	8.82	NS	2.5
ENSG00000104765	**BNIP3L**	8.24	70.31	8.54	NS	1.9	7.63	70.98	9.30	NS	1.9
ENSG00000172331	**BPGM**	3.07	14.99	4.89	NS	0.8	3.39	21.69	6.39	NS	1.0
ENSG00000172270	**BSG**	31.25	217.92	6.97	NS	0.6	26.52	257.95	9.73	9.90E-03	0.7
ENSG00000159388	**BTG2**	3.89	100.21	25.78	3.00E-03	22.6	1.07	214.14	199.33	1.00E-03	37.7
ENSG00000166167	**BTRC**	4.20	22.71	5.41	NS	1.3	4.06	25.29	6.24	NS	1.3
ENSG00000125730	**C3**	1773.54	14.49	0.01	1.54E-07	5.7	1456.62	7.61	0.01	0.00E+00	3.3
ENSG00000104267	**CA2**	61.14	8.06	0.13	2.07E-06	0.7	69.17	28.19	0.41	1.00E-04	3.4
ENSG00000153113	**CAST**	10.61	74.21	7.00	NS	6.8	9.32	60.19	6.46	NS	5.1
ENSG00000121691	**CAT**	341.00	10.86	0.03	1.54E-07	0.9	400.58	21.17	0.05	5.49E-07	1.5
ENSG00000024862	**CCDC28A**	35.34	34.49	0.98	1.00E-03	2.2	23.52	26.61	1.13	1.00E-03	1.7
ENSG00000112576	**CCND3**	2.45	33.83	13.81	6.00E-04	3.3	1.59	32.98	20.76	4.40E-03	3.6
ENSG00000004897	**CDC27**	4.70	34.16	7.26	NS	1.1	5.05	53.99	10.69	NS	1.6
ENSG00000140743	**CDR2**	3.94	18.72	4.75	NS	0.6	4.47	21.49	4.80	NS	0.7
ENSG00000138433	**CIR1**	8.27	35.89	4.34	NS	2.2	8.53	31.08	3.64	NS	1.8
ENSG00000109572	**CLCN3**	5.22	50.33	9.64	NS	2.8	4.81	66.82	13.90	NS	3.2
ENSG00000155962	**CLIC2**	2.34	0.55	0.23	1.00E-03	1.2	0.87	0.47	0.53	7.00E-04	0.9
ENSG00000080819	**CPOX**	16.64	43.41	2.61	3.30E-03	1.6	22.58	40.24	1.78	3.40E-03	1.4
ENSG00000215908	**CROCCP2**	37.43	20.23	0.54	3.70E-03	1.6	44.32	18.51	0.42	2.60E-03	1.1
ENSG00000040531	**CTNS**	7.18	34.65	4.83	NS	2.1	7.08	42.14	5.95	NS	2.6
ENSG00000164733	**CTSB**	153.65	419.75	2.73	NS	30.7	100.71	281.13	2.79	6.70E-03	23.8
ENSG00000196188	**CTSE**	0.01	0.41	44.50	NS	31.3	0.07	13.75	195.65	1.00E-04	#DIV/0!
ENSG00000100592	**DAAM1**	2.85	10.15	3.56	NS	0.9	2.92	22.09	7.56	NS	1.7
ENSG00000122741	**DCAF10**	4.16	30.18	7.25	NS	4.3	4.47	26.96	6.03	NS	3.8
ENSG00000100897	**DCAF11**	66.78	32.32	0.48	4.67E-06	1.0	64.18	37.69	0.59	0.00E+00	0.9
ENSG00000043093	**DCUN1D1**	6.50	19.81	3.05	NS	1.1	6.16	19.39	3.15	NS	1.0

ENSG00000007968	**E2F2**	0.09	3.61	38.47	2.50E-03	0.8	0.02	4.24	221.94	2.00E-03	0.8
ENSG00000086232	**EIF2AK1**	25.81	62.97	2.44	2.00E-03	1.0	25.12	83.79	3.34	NS	1.1
ENSG00000118985	**ELL2**	23.01	54.46	2.37	1.40E-03	5.0	27.17	53.12	1.96	1.60E-03	4.5
ENSG00000149218	**ENDOD1**	2.03	53.61	26.46	4.00E-04	16.4	1.44	32.94	22.84	NS	11.0
ENSG00000159023	**EPB41**	10.94	25.67	2.35	1.60E-03	0.4	11.02	33.57	3.05	NS	0.6
ENSG00000187266	**EPOR**	11.16	9.49	0.85	NS	1.0	9.23	9.02	0.98	NS	0.9
ENSG00000164010	**ERMAP**	12.59	10.46	0.83	3.00E-04	3.7	8.55	8.95	1.05	4.00E-04	3.2
ENSG00000108799	**EZH1**	7.14	21.25	2.98	NS	1.9	7.70	20.23	2.63	NS	1.7
ENSG00000183508	**FAM46C**	2.51	1.61	0.64	1.00E-03	2.3	1.20	2.71	2.26	4.10E-03	3.4
ENSG00000178974	**FBXO34**	2.70	22.66	8.39	NS	1.7	2.50	32.08	12.81	NS	2.1
ENSG00000100225	**FBXO7**	23.38	63.72	2.73	NS	2.7	20.11	56.25	2.80	NS	2.3
ENSG00000112146	**FBXO9**	11.73	14.16	1.21	1.00E-04	1.1	10.53	14.90	1.41	3.00E-04	1.2
ENSG00000066926	**FECH**	8.78	44.39	5.05	NS	2.7	9.23	75.57	8.19	NS	3.0
ENSG00000167363	**FN3K**	27.31	4.88	0.18	0.00E+00	1.2	21.41	10.93	0.51	4.00E-04	2.7
ENSG00000065970	**FOXJ2**	3.56	30.50	8.58	6.70E-03	3.8	3.58	35.63	9.94	8.90E-03	4.1
ENSG00000118689	**FOXO3**	4.80	56.46	11.77	NS	4.3	4.22	39.09	9.27	NS	3.0
ENSG00000160282	**FTCD**	418.94	0.39	0.00	1.74E-07	0.6	677.61	1.75	0.00	5.37E-07	2.0
ENSG00000165219	**GAPVD1**	2.39	19.66	8.23	NS	1.5	2.57	24.86	9.68	NS	2.1
ENSG00000001084	**GCLC**	22.08	18.19	0.82	0.00E+00	0.8	26.65	42.87	1.61	1.40E-03	1.8
ENSG00000023909	**GCLM**	8.19	82.35	10.05	NS	7.4	7.58	97.64	12.89	NS	7.4
ENSG00000006007	**GDE1**	10.34	56.48	5.46	NS	1.5	10.43	70.02	6.71	NS	1.8
ENSG00000182512	**GLRX5**	22.89	55.83	2.44	2.40E-03	1.0	30.04	95.45	3.18	NS	1.4
ENSG00000163655	**GMPS**	2.71	36.63	13.53	1.90E-03	0.5	2.84	31.47	11.09	NS	0.5
ENSG00000136732	**GYPC**	2.84	34.52	12.16	3.40E-03	2.5	1.70	29.93	17.56	NS	1.9
ENSG00000197465	**GYPE**	0.18	0.00	0.00	7.60E-03	0.0	0.02	0.10	5.03	NS	6.5
ENSG00000189060	**H1F0**	101.20	377.14	3.73	NS	3.4	66.28	356.51	5.38	NS	3.7
ENSG00000063854	**HAGH**	54.31	10.06	0.19	4.63E-06	2.0	61.62	15.53	0.25	0.00E+00	3.2
ENSG00000244734	**HBB**	173.68	0.00	0.00	2.39E-06	#DIV/0!	32.39	0.08	0.00	7.11E-06	#DIV/0!
ENSG00000223609	**HBD**	0.41	0.00	0.00	2.00E-04	#DIV/0!	0.14	0.00	0.00	3.00E-04	#DIV/0!
ENSG00000086506	**HBQ1**	0.05	0.07	1.49	NS	0.0	0.04	0.48	12.43	NS	0.4
ENSG00000143321	**HDGF**	82.77	229.33	2.77	4.90E-03	0.8	105.13	224.21	2.13	6.30E-03	0.7
ENSG00000013583	**HEBP1**	24.35	45.07	1.85	3.00E-04	2.2	29.55	44.80	1.52	6.00E-04	2.0
ENSG00000256269	**HMBS**	3.93	10.58	2.69	NS	0.3	6.41	14.94	2.33	NS	0.4

ENSG00000109854	**HTATIP2**	15.44	43.78	2.83	5.40E-03	2.2	15.33	61.13	3.99	NS	3.0
ENSG00000115317	**HTRA2**	9.14	28.75	3.15	NS	2.2	8.94	28.86	3.23	NS	2.0
ENSG00000105371	**ICAM4**	0.15	0.10	0.66	NS	0.2	0.04	0.34	7.76	NS	0.4
ENSG00000143061	**IGSF3**	0.21	132.88	639.27	2.44E-07	4.5	0.23	90.81	402.73	2.42E-06	2.4
ENSG00000135070	**ISCA1**	8.55	43.91	5.14	NS	1.3	9.63	53.26	5.53	NS	1.1
ENSG00000114166	**KAT2B**	11.20	4.25	0.38	0.00E+00	5.3	10.20	8.51	0.83	2.00E-04	10.0
ENSG00000197993	**KEL**	0.03	0.25	7.95	NS	0.4	0.05	4.70	98.29	1.00E-04	3.4
ENSG00000100441	**KHNYN**	12.06	95.74	7.94	NS	5.9	11.91	90.00	7.56	NS	4.0
ENSG00000105610	**KLF1**	0.14	0.10	0.72	NS	0.1	0.04	0.17	4.21	NS	0.3
ENSG00000109787	**KLF3**	12.92	93.17	7.21	NS	8.5	12.29	63.21	5.14	NS	4.6
ENSG00000005893	**LAMP2**	43.74	113.26	2.59	NS	4.8	36.59	137.60	3.76	NS	6.1
ENSG00000135363	**LMO2**	4.53	0.20	0.04	0.00E+00	0.1	1.87	3.90	2.09	6.10E-03	3.3
ENSG00000101577	**LPIN2**	28.14	28.91	1.03	1.00E-04	2.2	34.43	47.49	1.38	1.40E-03	3.4
ENSG00000197324	**LRP10**	8.95	86.30	9.64	8.40E-03	8.1	6.15	82.51	13.42	NS	6.9
ENSG00000034152	**MAP2K3**	21.65	19.91	0.92	1.00E-04	0.8	20.87	19.97	0.96	1.00E-04	0.8
ENSG00000117791	**MARC2**	121.94	11.47	0.09	3.37E-07	1.5	135.21	8.72	0.06	2.48E-06	1.0
ENSG00000165406	**MARC8**	4.35	8.58	1.97	2.00E-03	1.4	3.80	9.40	2.48	3.40E-03	1.6
ENSG00000075413	**MARK3**	6.23	36.28	5.82	NS	1.4	6.01	39.12	6.51	NS	1.2
ENSG00000143797	**MBOAT2**	0.28	71.87	253.89	3.02E-06	8.4	0.32	32.31	100.20	3.00E-04	3.3
ENSG00000147324	**MFHAS1**	2.62	62.22	23.77	2.60E-03	1.4	1.88	48.76	25.87	9.80E-03	1.2
ENSG00000143198	**MGST3**	7.32	29.56	4.04	NS	2.8	9.02	25.79	2.86	NS	1.9
ENSG00000107789	**MINPP1**	8.35	25.92	3.11	7.60E-03	0.8	12.42	32.67	2.63	NS	1.0
ENSG00000133606	**MKRN1**	11.88	60.77	5.12	NS	1.1	11.77	58.75	4.99	NS	1.2
ENSG00000075643	**MOCOS**	9.26	5.91	0.64	0.00E+00	0.6	10.25	4.23	0.41	0.00E+00	0.5
ENSG00000101928	**MOSPD1**	1.74	75.14	43.10	1.00E-04	4.3	1.79	82.86	46.20	1.00E-04	5.3
ENSG00000130830	**MPP1**	8.90	5.02	0.56	0.00E+00	0.6	6.80	11.69	1.72	5.00E-04	1.3
ENSG00000119950	**MXI1**	10.51	15.03	1.43	4.00E-04	2.7	11.50	28.10	2.44	NS	5.4
ENSG00000198336	**MYL4**	0.19	0.48	2.47	NS	1.1	0.14	196.95	1404.31	1.60E-03	302.0
ENSG00000141562	**NARF**	4.90	9.77	1.99	NS	0.6	4.78	12.67	2.65	NS	0.8
ENSG00000266412	**NCOA4**	83.67	186.59	2.23	NS	2.4	73.05	180.91	2.48	7.90E-03	2.1
ENSG00000151414	**NEK7**	6.81	42.91	6.30	NS	4.9	6.34	37.57	5.92	NS	4.7
ENSG00000123405	**NFE2**	0.21	0.42	2.02	NS	0.2	0.09	0.58	6.28	NS	0.2
ENSG00000082641	**NFE2L1**	61.28	217.11	3.54	NS	1.5	46.76	231.17	4.94	NS	1.6

ENSG00000112992	**NNT**	34.74	41.59	1.20	1.60E-03	3.2	22.20	36.50	1.64	1.20E-03	2.7
ENSG00000113580	**NR3C1**	9.04	24.56	2.72	NS	6.7	9.21	32.14	3.49	NS	5.7
ENSG00000173598	**NUDT4**	3.60	12.81	3.56	NS	1.1	4.93	31.59	6.41	NS	2.6
ENSG00000123240	**OPTN**	26.13	125.47	4.80	NS	13.5	18.74	97.75	5.22	NS	10.0
ENSG00000184792	**OSBP2**	0.18	0.73	3.99	NS	0.2	0.18	1.56	8.49	NS	0.5
ENSG00000072682	**P4HA2**	1.41	20.48	14.55	1.00E-04	9.1	1.29	25.34	19.68	3.00E-04	14.8
ENSG00000173599	**PC**	39.27	3.08	0.08	6.02E-07	0.4	36.26	9.69	0.27	8.61E-06	1.1
ENSG00000162366	**PDZK1IP1**	0.29	0.00	0.00	2.76E-06	0.0	0.52	0.06	0.12	0.00E+00	0.7
ENSG00000130313	**PGLS**	12.97	69.90	5.39	NS	2.9	13.16	57.68	4.38	NS	2.0
ENSG00000073921	**PICALM**	23.21	196.98	8.49	NS	4.4	21.89	144.06	6.58	NS	3.5
ENSG00000007541	**PIGQ**	7.93	18.21	2.30	NS	1.5	8.85	16.82	1.90	NS	1.4
ENSG00000143224	**PPOX**	5.46	7.85	1.44	NS	0.8	5.06	7.56	1.49	NS	0.8
ENSG00000068971	**PPP2R5B**	4.14	11.64	2.81	NS	2.7	3.75	13.94	3.72	NS	3.5
ENSG00000122490	**PQLC1**	47.76	17.94	0.38	1.00E-04	3.2	53.76	16.16	0.30	1.00E-04	3.6
ENSG00000167815	**PRDX2**	74.84	118.20	1.58	3.00E-04	0.4	83.89	175.46	2.09	5.80E-03	0.7
ENSG00000110801	**PSMD9**	3.87	16.60	4.29	NS	2.7	4.16	18.61	4.47	NS	2.9
ENSG00000179262	**RAD23A**	37.61	127.74	3.40	NS	2.4	50.40	154.52	3.07	NS	3.4
ENSG00000141084	**RANBP10**	23.99	22.49	0.94	6.00E-04	1.7	21.82	23.36	1.07	8.00E-04	1.7
ENSG00000076864	**RAP1GAP**	4.96	1.55	0.31	0.00E+00	0.3	4.55	8.84	1.94	NS	1.4
ENSG00000132819	**RBM38**	3.75	54.28	14.46	2.00E-04	0.9	2.74	42.67	15.56	1.70E-03	0.8
ENSG00000003756	**RBM5**	18.02	74.22	4.12	NS	3.5	20.01	75.54	3.77	NS	2.7
ENSG00000120158	**RCL1**	16.40	12.87	0.78	0.00E+00	0.7	27.04	13.70	0.51	1.00E-04	0.7
ENSG00000188672	**RHCE**	0.90	0.43	0.48	2.50E-03	2.4	0.40	0.19	0.48	3.00E-04	2.4
ENSG00000101782	**RIOK3**	19.84	111.08	5.60	NS	8.0	18.32	94.95	5.18	NS	6.7
ENSG00000164068	**RNF123**	19.80	14.72	0.74	1.00E-04	1.1	21.12	15.44	0.73	1.00E-04	1.0
ENSG00000034677	**RNF19A**	8.73	47.38	5.43	NS	6.1	6.88	43.45	6.32	NS	7.4
ENSG00000137575	**SDCBP**	16.67	340.17	20.40	1.90E-03	5.2	14.39	251.11	17.45	NS	3.7
ENSG00000129657	**SEC14L1**	3.52	55.22	15.70	NS	2.7	1.81	48.28	26.75	NS	2.4
ENSG00000143416	**SELENBP1**	221.61	0.98	0.00	5.69E-07	0.1	271.49	4.58	0.02	1.87E-06	0.3
ENSG00000149577	**SIDT2**	8.01	9.08	1.13	0.00E+00	1.5	7.88	11.44	1.45	2.00E-04	1.9
ENSG00000126903	**SLC10A3**	3.98	73.93	18.59	1.00E-04	6.7	2.71	50.83	18.75	3.70E-03	5.2
ENSG00000110911	**SLC11A2**	6.15	17.89	2.91	6.50E-03	0.9	8.21	28.90	3.52	NS	1.2
ENSG00000197208	**SLC22A4**	0.21	11.06	52.93	4.06E-06	32.8	0.23	1.91	8.19	NS	4.7

ENSG00000147454	**SLC25A37**	15.25	43.10	2.83	NS	2.6	20.64	27.11	1.31	NS	1.5
ENSG00000144659	**SLC25A38**	33.50	52.33	1.56	7.00E-04	1.0	36.56	85.22	2.33	NS	1.6
ENSG00000117394	**SLC2A1**	0.55	199.17	365.17	0.00E+00	1.1	0.19	192.83	997.52	1.00E-04	0.9
ENSG00000170385	**SLC30A1**	24.53	68.89	2.81	NS	5.2	18.27	86.06	4.71	NS	6.7
ENSG00000196660	**SLC30A10**	8.51	0.12	0.01	5.01E-06	0.5	10.73	0.92	0.09	0.00E+00	7.6
ENSG00000130821	**SLC6A8**	0.20	9.27	45.40	1.00E-04	0.2	0.12	21.51	182.91	0.00E+00	0.4
ENSG00000196517	**SLC6A9**	0.10	60.74	611.17	0.00E+00	5.3	0.19	38.33	201.73	1.00E-04	3.5
ENSG00000151012	**SLC7A11**	0.02	74.85	4224.00	3.87E-08	3.3	0.03	164.71	5440.14	1.81E-07	6.8
ENSG00000088826	**SMOX**	0.98	61.28	62.66	6.63E-06	7.0	0.98	74.16	75.90	0.00E+00	8.6
ENSG00000145335	**SNCA**	0.08	1.03	13.08	NS	0.1	0.02	5.44	349.01	NS	0.7
ENSG00000070182	**SPTB**	0.08	0.11	1.42	NS	0.1	0.04	1.36	36.95	NS	1.8
ENSG00000159082	**SYNJ1**	2.36	9.17	3.89	NS	6.6	2.13	11.13	5.22	NS	5.8
ENSG00000162367	**TAL1**	0.86	0.01	0.01	1.09E-06	0.2	0.48	0.03	0.06	2.64E-06	0.6
ENSG00000187735	**TCEA1**	12.82	155.94	12.17	NS	1.4	13.69	174.03	12.71	NS	1.7
ENSG00000114126	**TFDP2**	2.15	10.04	4.66	NS	0.3	2.28	13.27	5.82	NS	0.3
ENSG00000072274	**TFRC**	3.89	109.65	28.20	2.10E-03	1.4	7.52	144.40	19.21	1.50E-03	1.9
ENSG00000133069	**TMCC2**	0.18	3.92	21.34	5.60E-03	0.9	0.05	5.03	107.82	5.60E-03	1.2
ENSG00000175348	**TMEM9B**	8.03	38.83	4.83	NS	3.7	5.80	49.55	8.54	NS	4.7
ENSG00000100354	**TNRC6B**	1.84	9.27	5.05	NS	0.8	2.38	9.91	4.17	NS	0.9
ENSG00000079308	**TNS1**	20.02	7.71	0.39	5.08E-06	4.2	13.31	4.52	0.34	1.00E-04	1.8
ENSG00000198900	**TOP1**	12.87	91.97	7.14	NS	0.7	12.87	103.94	8.08	NS	0.7
ENSG00000115993	**TRAK2**	3.62	22.87	6.31	NS	1.9	3.08	32.31	10.48	NS	2.4
ENSG00000204613	**TRIM10**	1.40	0.00	0.00	2.75E-08	0.0	1.02	0.00	0.00	4.97E-08	0.0
ENSG00000162722	**TRIM58**	0.02	8.06	355.02	1.00E-03	5.2	0.01	5.74	888.02	3.30E-03	2.8
ENSG00000168785	**TSPAN5**	0.22	11.71	53.98	7.00E-04	1.2	0.37	15.73	42.14	5.00E-04	1.4
ENSG00000130560	**UBAC1**	9.39	34.61	3.69	NS	0.7	10.36	41.68	4.02	NS	0.9
ENSG00000175567	**UCP2**	4.74	4.24	0.90	NS	0.2	1.28	17.62	13.74	NS	0.4
ENSG00000126088	**UROD**	43.10	51.55	1.20	1.00E-03	0.9	50.86	66.83	1.31	3.30E-03	1.0
ENSG00000188690	**UROS**	6.50	11.67	1.79	1.60E-03	0.6	6.34	14.02	2.21	4.80E-03	0.8
ENSG00000135655	**USP15**	4.69	9.79	2.09	7.00E-04	1.6	5.24	11.03	2.10	1.90E-03	1.6
ENSG00000136451	**VEZF1**	6.06	73.19	12.08	1.20E-03	1.9	5.15	65.46	12.70	6.50E-03	1.5
ENSG00000047597	**XK**	0.44	0.38	0.86	6.00E-04	0.3	0.32	0.51	1.58	1.10E-03	0.4
ENSG00000130227	**XPO7**	5.72	50.47	8.83	NS	0.7	6.11	65.79	10.76	NS	0.9

ENSG00000119801	**YPEL5**	21.88	179.98	8.23	4.70E-03	8.0	22.39	91.27	4.08	NS	4.9

Hallmark Coagulation

ENSG00000175899	**A2M**	280.44	3.74	0.01	2.18E-07	5.7	275.03	15.46	0.06	1.26E-06	34.8
ENSG00000168306	**ACOX2**	116.27	0.29	0.00	1.50E-08	0.1	166.28	4.61	0.03	3.85E-06	1.6
ENSG00000168615	**ADAM9**	1.33	165.50	124.29	3.30E-03	14.0	1.26	208.89	165.63	2.80E-03	15.0
ENSG00000214274	**ANG**	548.51	2.55	0.00	7.74E-09	0.9	710.63	7.80	0.01	6.41E-08	3.0
ENSG00000135046	**ANXA1**	2.77	737.14	265.84	0.00E+00	82.9	1.23	412.95	335.69	3.00E-04	133.1
ENSG00000118137	**APOA1**	11000.82	13.80	0.00	1.39E-07	21.7	8593.47	899.42	0.10	1.00E-04	429.3
ENSG00000130208	**APOC1**	5444.72	12.09	0.00	5.89E-07	0.3	5644.64	204.01	0.04	0.00E+00	3.4
ENSG00000234906	**APOC2**	3348.84	0.52	0.00	6.04E-09	18.9	2704.47	2.86	0.00	5.31E-08	6.4
ENSG00000110245	**APOC3**	10956.82	0.79	0.00	1.36E-08	10.2	12127.76	9.24	0.00	3.69E-08	4.8
ENSG00000168374	**ARF4**	39.23	322.32	8.22	NS	1.5	47.98	338.02	7.05	NS	1.7
ENSG00000168487	**BMP1**	14.68	74.84	5.10	NS	29.2	19.65	41.01	2.09	NS	13.2
ENSG00000173372	**C1QA**	67.20	0.00	0.00	4.08E-06	#DIV/0!	22.25	0.03	0.00	5.95E-06	1.2
ENSG00000159403	**C1R**	793.04	0.48	0.00	6.51E-10	0.1	660.37	5.11	0.01	2.11E-06	1.0
ENSG00000182326	**C1S**	722.07	1.59	0.00	3.18E-09	0.5	647.15	13.04	0.02	6.05E-07	2.4
ENSG00000166278	**C2**	81.25	0.20	0.00	9.58E-09	2.1	68.43	0.26	0.00	1.25E-08	2.0
ENSG00000125730	**C3**	1773.54	14.49	0.01	1.54E-07	5.7	1456.62	7.61	0.01	0.00E+00	3.3
ENSG00000157131	**C8A**	278.31	0.11	0.00	1.08E-07	#DIV/0!	266.03	0.27	0.00	6.62E-08	3.8
ENSG00000021852	**C8B**	228.79	0.12	0.00	3.52E-07	9.6	227.71	0.57	0.00	4.82E-07	15.5
ENSG00000176919	**C8G**	304.86	0.26	0.00	3.51E-07	1.3	190.17	0.42	0.00	3.47E-08	3.2
ENSG00000113600	**C9**	432.26	0.04	0.00	8.24E-08	1.2	183.18	0.12	0.00	1.34E-07	0.6
ENSG00000162909	**CAPN2**	8.40	383.97	45.71	0.00E+00	25.4	3.61	265.81	73.71	1.00E-04	19.4
ENSG00000149260	**CAPN5**	22.69	10.48	0.46	3.40E-06	0.6	28.65	15.06	0.53	0.00E+00	0.8
ENSG00000132906	**CASP9**	4.95	7.96	1.61	NS	0.9	2.95	9.86	3.34	NS	0.9
ENSG00000010278	**CD9**	6.62	228.24	34.50	1.00E-04	2.3	5.29	97.34	18.39	NS	0.9
ENSG00000243649	**CFB**	463.52	0.32	0.00	7.08E-08	9.3	359.88	1.70	0.00	3.80E-07	11.7
ENSG00000197766	**CFD**	1.14	0.12	0.11	1.00E-03	0.5	0.32	0.73	2.24	NS	1.9
ENSG00000000971	**CFH**	354.48	0.00	0.00	1.96E-09	0.0	287.45	0.37	0.00	1.19E-07	6.5
ENSG00000205403	**CFI**	300.31	0.22	0.00	1.93E-08	#DIV/0!	224.35	6.86	0.03	1.95E-06	265.5
ENSG00000120885	**CLU**	859.16	23.62	0.03	1.77E-08	0.2	749.50	52.36	0.07	2.38E-07	0.7
ENSG00000105664	**COMP**	0.09	0.01	0.14	NS	0.2	0.01	0.10	14.40	NS	0.6
ENSG00000080618	**CPB2**	715.57	0.00	0.00	7.47E-10	#DIV/0!	545.00	0.20	0.00	2.77E-09	1.5

ENSG00000120054	**CPN1**	25.92	0.33	0.01	2.75E-08	2.1	23.18	10.47	0.45	3.00E-04	13.6
ENSG00000182809	**CRIP2**	5.60	0.26	0.05	9.86E-07	0.2	4.47	3.83	0.86	8.40E-03	2.9
ENSG00000159176	**CSRP1**	17.24	50.82	2.95	NS	7.1	15.99	35.90	2.25	NS	5.2
ENSG00000164733	**CTSB**	153.65	419.75	2.73	NS	30.7	100.71	281.13	2.79	6.70E-03	23.8
ENSG00000196188	**CTSE**	0.01	0.41	44.50	NS	31.3	0.07	13.75	195.65	1.00E-04	#DIV/0!
ENSG00000103811	**CTSH**	15.38	1.44	0.09	1.67E-06	0.2	32.69	4.18	0.13	0.00E+00	0.7
ENSG00000143387	**CTSK**	6.94	6.64	0.96	7.90E-03	1.0	4.79	6.78	1.42	7.40E-03	1.0
ENSG00000256043	**CTSO**	18.67	8.72	0.47	0.00E+00	9.7	15.74	6.99	0.44	2.00E-04	8.0
ENSG00000080166	**DCT**	0.30	0.09	0.31	5.00E-04	3.5	0.14	2.97	21.91	NS	67.0
ENSG00000197635	**DPP4**	14.18	9.55	0.67	1.00E-04	2.5	24.22	137.71	5.69	NS	42.3
ENSG00000276023	**DUSP14**	2.89	36.78	12.72	1.30E-03	3.7	3.54	44.36	12.53	1.40E-03	4.8
ENSG00000139318	**DUSP6**	17.32	48.77	2.82	NS	0.7	11.26	36.12	3.21	NS	0.3
ENSG00000126218	**F10**	77.41	0.29	0.00	3.90E-08	0.4	70.46	11.16	0.16	0.00E+00	4.3
ENSG00000088926	**F11**	92.80	0.02	0.00	7.33E-10	0.0	120.20	0.04	0.00	3.75E-09	0.2
ENSG00000131187	**F12**	350.76	0.55	0.00	1.11E-07	0.4	557.22	1.40	0.00	5.76E-07	1.4
ENSG00000143278	**F13B**	74.90	0.00	0.00	4.18E-08	#DIV/0!	82.96	0.09	0.00	3.10E-07	6.2
ENSG00000180210	**F2**	987.30	1.17	0.00	7.84E-08	11.0	834.58	20.52	0.02	1.21E-07	98.8
ENSG00000164220	**F2RL2**	0.66	0.27	0.40	NS	0.2	0.09	10.38	121.91	NS	38.2
ENSG00000117525	**F3**	1.20	24.73	20.65	4.00E-04	2.7	0.43	66.00	154.20	4.00E-04	13.9
ENSG00000185010	**F8**	2.69	1.47	0.55	3.30E-03	1.4	1.32	0.89	0.68	1.30E-03	0.6
ENSG00000101981	**F9**	279.73	0.00	0.00	6.28E-09	#DIV/0!	195.21	0.08	0.00	1.85E-08	1.0
ENSG00000166147	**FBN1**	0.75	10.97	14.57	NS	2.0	0.38	27.22	71.28	NS	5.4
ENSG00000171560	**FGA**	3563.87	33.57	0.01	7.81E-07	48.3	2533.82	502.27	0.20	5.32E-06	633.0
ENSG00000171557	**FGG**	2253.42	21.40	0.01	1.67E-06	55.8	1507.47	173.50	0.12	1.00E-04	410.9
ENSG00000115414	**FN1**	272.71	1865.14	6.84	NS	31.9	256.27	2388.22	9.32	NS	62.2
ENSG00000140564	**FURIN**	102.48	173.72	1.70	1.20E-03	5.9	91.83	125.72	1.37	7.00E-04	5.0
ENSG00000010810	**FYN**	5.78	48.64	8.41	4.70E-03	2.2	4.22	48.76	11.55	NS	2.0
ENSG00000119125	**GDA**	16.42	0.43	0.03	2.33E-08	0.9	14.14	0.52	0.04	5.00E-07	1.9
ENSG00000172354	**GNB2**	37.17	190.71	5.13	NS	2.2	39.85	187.88	4.71	NS	2.2
ENSG00000172380	**GNG12**	9.69	90.15	9.31	NS	2.6	8.06	77.13	9.57	NS	2.0
ENSG00000148180	**GSN**	6.57	44.00	6.69	NS	4.3	2.43	59.43	24.42	NS	5.9
ENSG00000134240	**HMGCS2**	874.78	2.85	0.00	1.00E-06	73.8	1059.55	27.36	0.03	1.64E-07	133.9
ENSG00000101076	**HNF4A**	100.42	0.57	0.01	3.22E-08	92.0	93.59	32.79	0.35	4.00E-04	553.1

ENSG00000105707	**HPN**	374.09	15.06	0.04	1.72E-06	1.4	396.67	30.54	0.08	5.11E-06	3.0
ENSG00000113905	**HRG**	1116.51	0.06	0.00	3.54E-09	0.7	965.07	0.82	0.00	1.09E-07	2.9
ENSG00000166033	**HTRA1**	45.34	1.15	0.03	7.40E-08	0.0	31.27	12.11	0.39	0.00E+00	0.4
ENSG00000136003	**ISCU**	18.78	72.31	3.85	NS	2.1	19.06	86.56	4.54	NS	2.5
ENSG00000164171	**ITGA2**	0.08	256.71	3326.75	1.00E-07	41.4	0.06	63.24	999.25	0.00E+00	15.9
ENSG00000259207	**ITGB3**	0.95	3.41	3.59	NS	25.0	0.63	5.51	8.72	NS	45.4
ENSG00000055957	**ITIH1**	727.41	0.01	0.00	1.29E-09	#DIV/0!	764.58	0.27	0.00	3.43E-09	1.3
ENSG00000118263	**KLF7**	2.31	22.59	9.79	NS	2.1	2.11	13.40	6.34	NS	1.1
ENSG00000129455	**KLK8**	0.01	1.76	134.77	7.71E-06	1.3	0.01	1.44	128.58	NS	1.3
ENSG00000164344	**KLKB1**	164.07	0.32	0.00	1.33E-09	0.0	170.45	0.27	0.00	5.59E-09	0.0
ENSG00000005893	**LAMP2**	43.74	113.26	2.59	NS	4.8	36.59	137.60	3.76	NS	6.1
ENSG00000143768	**LEFTY2**	0.07	0.18	2.56	NS	0.0	0.06	2.87	46.97	NS	0.2
ENSG00000100600	**LGMN**	22.16	64.88	2.93	NS	1.7	19.00	74.32	3.91	NS	1.3
ENSG00000123384	**LRP1**	44.14	76.99	1.74	9.00E-04	1.7	39.32	111.59	2.84	9.60E-03	2.5
ENSG00000111144	**LTA4H**	7.78	35.05	4.51	NS	0.5	6.12	35.92	5.86	NS	0.5
ENSG00000185022	**MAFF**	0.32	27.25	84.69	1.00E-04	8.6	0.14	16.31	115.54	3.00E-04	6.8
ENSG00000009724	**MASP2**	61.33	0.02	0.00	4.24E-10	0.1	66.83	0.13	0.00	6.53E-09	0.2
ENSG00000165471	**MBL2**	38.25	0.28	0.01	7.57E-06	#DIV/0!	63.80	0.93	0.01	0.00E+00	50.1
ENSG00000112818	**MEP1A**	0.01	0.75	91.25	NS	32.1	0.02	2.89	137.86	NS	#DIV/0!
ENSG00000196611	**MMP1**	0.01	0.19	15.66	NS	0.6	0.01	11.82	1116.54	7.00E-04	719.2
ENSG00000166670	**MMP10**	0.01	0.20	14.66	1.00E-03	0.3	0.01	0.74	62.73	5.00E-04	3.3
ENSG00000099953	**MMP11**	0.19	0.56	2.90	NS	0.3	0.08	2.77	32.77	1.50E-03	1.3
ENSG00000157227	**MMP14**	5.72	525.09	91.87	3.78E-06	9.9	3.10	498.85	161.03	7.19E-06	6.3
ENSG00000102996	**MMP15**	19.50	56.58	2.90	NS	1.6	20.52	55.21	2.69	NS	1.6
ENSG00000087245	**MMP2**	2.86	269.63	94.41	0.00E+00	10.6	1.11	127.41	114.57	NS	5.5
ENSG00000149968	**MMP3**	0.01	0.08	6.71	NS	#DIV/0!	0.01	0.04	3.94	NS	2.5
ENSG00000137673	**MMP7**	0.47	0.02	0.04	1.00E-04	#DIV/0!	0.94	0.05	0.06	2.00E-04	#DIV/0!
ENSG00000100985	**MMP9**	0.52	41.98	80.33	1.00E-04	13.8	0.10	5.16	52.57	NS	1.3
ENSG00000148450	**MSRB2**	23.18	19.67	0.85	1.00E-04	2.3	26.87	23.78	0.89	1.00E-04	2.2
ENSG00000173531	**MST1**	233.30	5.38	0.02	8.84E-06	3.2	223.95	7.11	0.03	0.00E+00	4.3
ENSG00000173391	**OLR1**	0.14	0.24	1.77	NS	#DIV/0!	0.03	5.54	162.38	NS	523.0
ENSG00000169860	**P2RY1**	0.08	1.49	18.58	NS	0.1	0.04	1.45	33.43	NS	0.1
ENSG00000100311	**PDGFB**	1.88	59.13	31.41	2.00E-04	10.2	2.61	30.43	11.65	NS	9.1

ENSG00000261371	**PECAM1**	3.94	0.08	0.02	0.00E+00	0.2	1.58	0.18	0.12	2.00E-04	0.4
ENSG00000162517	**PEF1**	21.61	115.51	5.35	NS	2.4	28.10	134.10	4.77	NS	2.6
ENSG00000163737	**PF4**	0.69	0.00	0.00	2.80E-03	0.0	0.14	0.00	0.00	2.90E-03	0.0
ENSG00000104368	**PLAT**	1.41	7.52	5.33	NS	2.8	0.31	5.01	16.05	NS	0.8
ENSG00000122861	**PLAU**	0.93	136.95	147.74	9.51E-06	4.1	0.37	28.48	76.00	2.80E-03	0.8
ENSG00000115956	**PLEK**	3.12	0.06	0.02	1.00E-04	6.0	1.06	0.08	0.07	1.00E-04	1.9
ENSG00000122194	**PLG**	531.41	0.15	0.00	6.04E-09	7.3	580.42	0.29	0.00	2.06E-08	1.7
ENSG00000085377	**PREP**	6.19	38.87	6.28	NS	0.7	5.49	37.05	6.75	NS	0.6
ENSG00000115718	**PROC**	291.96	0.08	0.00	1.81E-08	0.1	321.57	1.16	0.00	2.27E-07	1.9
ENSG00000184500	**PROS1**	83.88	7.80	0.09	2.25E-06	6.1	74.24	49.81	0.67	1.00E-04	25.2
ENSG00000126231	**PROZ**	184.70	0.14	0.00	6.02E-07	7.3	106.03	0.84	0.01	2.74E-06	14.0
ENSG00000150687	**PRSS23**	1.73	44.92	25.99	5.00E-04	15.0	1.04	56.86	54.92	5.00E-04	26.5
ENSG00000183155	**RABIF**	3.09	23.29	7.53	NS	2.0	3.68	28.95	7.86	NS	2.9
ENSG00000136238	**RAC1**	46.35	308.54	6.66	NS	1.4	41.01	287.37	7.01	NS	1.3
ENSG00000079337	**RAPGEF3**	0.85	1.40	1.64	3.30E-03	1.1	0.62	1.03	1.66	7.00E-04	0.5
ENSG00000130988	**RGN**	162.41	0.37	0.00	4.15E-07	0.2	171.48	3.79	0.02	6.98E-07	3.2
ENSG00000160678	**S100A1**	0.89	0.13	0.14	8.69E-06	1.0	0.68	0.41	0.60	1.00E-04	0.9
ENSG00000189171	**S100A13**	13.43	128.35	9.56	2.60E-03	5.3	6.82	107.30	15.72	NS	3.9
ENSG00000197249	**SERPINA1**	3218.49	2.16	0.00	2.41E-08	28.9	2099.11	11.57	0.01	6.39E-07	23.5
ENSG00000197632	**SERPINB2**	0.01	0.98	110.63	2.78E-06	2.1	0.01	1.56	205.10	NS	#DIV/0!
ENSG00000117601	**SERPINC1**	2139.15	0.79	0.00	1.82E-09	#DIV/0!	2028.29	11.99	0.01	5.63E-07	20.7
ENSG00000106366	**SERPINE1**	6.63	623.82	94.12	2.00E-04	70.5	17.41	294.22	16.90	3.80E-03	30.8
ENSG00000149131	**SERPING1**	1068.32	133.31	0.12	1.16E-06	4.3	759.02	93.00	0.12	0.00E+00	2.4
ENSG00000160999	**SH2B2**	0.72	5.99	8.36	6.10E-03	0.8	0.45	8.58	19.22	2.50E-03	1.1
ENSG00000068903	**SIRT2**	11.16	20.47	1.83	9.00E-04	6.6	10.74	24.90	2.32	4.30E-03	8.5
ENSG00000113140	**SPARC**	29.27	577.14	19.71	NS	2.7	13.68	828.99	60.61	6.90E-03	3.9
ENSG00000091513	**TF**	2547.50	0.99	0.00	1.17E-08	13.3	2049.92	12.10	0.01	1.52E-07	23.6
ENSG00000105825	**TFPI2**	0.44	84.87	192.84	NS	9.0	0.35	20.34	57.72	NS	2.2
ENSG00000178726	**THBD**	1.69	50.17	29.62	4.00E-04	110.8	0.51	33.32	65.64	1.30E-03	101.2
ENSG00000137801	**THBS1**	2.79	458.39	164.53	2.00E-04	14.8	0.89	139.88	157.07	2.90E-03	5.1
ENSG00000102265	**TIMP1**	50.91	148.58	2.92	NS	2.8	17.47	146.55	8.39	NS	2.6
ENSG00000100234	**TIMP3**	18.35	281.81	15.36	3.60E-03	40.0	13.89	215.61	15.52	NS	23.2
ENSG00000187045	**TMPRSS6**	130.48	0.11	0.00	1.77E-07	3.4	135.29	0.84	0.01	1.80E-07	10.4

ENSG00000102226	**USP11**	4.23	54.76	12.96	2.00E-04	0.9	4.42	64.25	14.53	4.00E-04	1.2
ENSG00000110799	**VWF**	2.20	1.73	0.79	4.30E-03	3.4	0.76	0.77	1.02	2.50E-03	0.5
ENSG00000071127	**WDR1**	11.41	340.01	29.81	0.00E+00	2.4	10.60	325.80	30.73	1.00E-04	2.3

Hallmark Complement

ENSG00000077522	**ACTN2**	0.02	2.49	112.92	1.00E-04	1.6	0.02	1.79	95.10	NS	1.1
ENSG00000168615	**ADAM9**	1.33	165.50	124.29	3.30E-03	14.0	1.26	208.89	165.63	2.80E-03	15.0
ENSG00000274286	**ADRA2B**	4.02	0.72	0.18	6.35E-06	0.1	3.06	1.06	0.35	2.00E-04	0.2
ENSG00000108599	**AKAP10**	3.88	30.25	7.80	NS	2.6	3.67	35.10	9.57	NS	2.9
ENSG00000214274	**ANG**	548.51	2.55	0.00	7.74E-09	0.9	710.63	7.80	0.01	6.41E-08	3.0
ENSG00000164111	**ANXA5**	25.16	769.00	30.57	1.00E-04	3.2	13.22	614.27	46.46	3.00E-04	2.9
ENSG00000110244	**APOA4**	28.41	4.83	0.17	5.00E-04	71.6	10.25	61.28	5.98	NS	2667.0
ENSG00000128394	**APOBEC3F**	1.44	3.92	2.72	NS	1.3	0.76	2.77	3.65	NS	1.0
ENSG00000239713	**APOBEC3G**	1.47	2.07	1.41	NS	3.2	0.47	0.75	1.61	5.10E-03	1.1
ENSG00000130208	**APOC1**	5444.72	12.09	0.00	5.89E-07	0.3	5644.64	204.01	0.04	0.00E+00	3.4
ENSG00000177556	**ATOX1**	13.69	11.87	0.87	6.00E-04	1.3	18.87	15.30	0.81	1.40E-03	1.7
ENSG00000096070	**BRPF3**	4.86	65.16	13.42	NS	2.3	6.40	67.14	10.49	NS	2.3
ENSG00000173372	**C1QA**	67.20	0.00	0.00	4.08E-06	#DIV/0!	22.25	0.03	0.00	5.95E-06	1.2
ENSG00000159189	**C1QC**	32.85	0.00	0.00	1.00E-04	#DIV/0!	7.72	0.00	0.00	1.00E-04	#DIV/0!
ENSG00000159403	**C1R**	793.04	0.48	0.00	6.51E-10	0.1	660.37	5.11	0.01	2.11E-06	1.0
ENSG00000182326	**C1S**	722.07	1.59	0.00	3.18E-09	0.5	647.15	13.04	0.02	6.05E-07	2.4
ENSG00000166278	**C2**	81.25	0.20	0.00	9.58E-09	2.1	68.43	0.26	0.00	1.25E-08	2.0
ENSG00000125730	**C3**	1773.54	14.49	0.01	1.54E-07	5.7	1456.62	7.61	0.01	0.00E+00	3.3
ENSG00000123843	**C4BPB**	144.00	0.48	0.00	1.48E-08	#DIV/0!	144.81	2.67	0.02	2.56E-07	#DIV/0!
ENSG00000113600	**C9**	432.26	0.04	0.00	8.24E-08	1.2	183.18	0.12	0.00	1.34E-07	0.6
ENSG00000104267	**CA2**	61.14	8.06	0.13	2.07E-06	0.7	69.17	28.19	0.41	1.00E-04	3.4
ENSG00000198668	**CALM1**	31.51	143.60	4.56	NS	1.3	32.73	188.25	5.75	NS	1.1
ENSG00000160014	**CALM3**	45.77	201.24	4.40	NS	0.9	47.59	273.20	5.74	NS	1.1
ENSG00000137752	**CASP1**	3.78	0.24	0.06	0.00E+00	#DIV/0!	1.68	0.23	0.14	0.00E+00	13.0
ENSG00000003400	**CASP10**	3.12	0.36	0.12	2.88E-07	0.1	2.30	2.17	0.94	1.10E-03	1.5
ENSG00000164305	**CASP3**	12.54	110.74	8.83	2.30E-03	0.9	9.73	82.36	8.47	NS	0.7
ENSG00000196954	**CASP4**	5.72	7.03	1.23	1.20E-03	51.0	3.33	6.20	1.86	NS	134.2
ENSG00000137757	**CASP5**	0.05	0.07	1.57	NS	#DIV/0!	0.03	0.13	5.03	NS	#DIV/0!
ENSG00000165806	**CASP7**	6.25	24.41	3.91	NS	6.5	8.02	15.74	1.96	NS	3.6

ENSG00000132906	**CASP9**	4.95	7.96	1.61	NS	0.9	2.95	9.86	3.34	NS	0.9
ENSG00000114423	**CBLB**	1.99	17.64	8.85	7.40E-03	2.0	2.37	16.30	6.87	NS	1.9
ENSG00000271503	**CCL5**	6.67	0.05	0.01	0.00E+00	0.4	1.45	0.03	0.02	0.00E+00	0.6
ENSG00000135218	**CD36**	3.83	0.01	0.00	1.93E-06	0.0	4.19	0.11	0.03	0.00E+00	0.3
ENSG00000102245	**CD40LG**	0.42	0.00	0.00	5.26E-06	#DIV/0!	0.32	0.00	0.00	6.31E-06	#DIV/0!
ENSG00000117335	**CD46**	22.36	97.91	4.38	NS	1.5	26.52	99.17	3.74	NS	1.4
ENSG00000196352	**CD55**	2.09	32.23	15.43	NS	2.8	1.47	87.61	59.57	8.10E-03	8.2
ENSG00000085063	**CD59**	39.62	186.94	4.72	NS	5.1	34.45	141.54	4.11	NS	4.0
ENSG00000158825	**CDA**	34.51	0.00	0.00	1.04E-08	0.0	36.58	0.32	0.01	1.99E-06	0.0
ENSG00000140945	**CDH13**	0.16	1.75	10.88	NS	1.1	0.03	2.80	107.60	NS	3.5
ENSG00000176749	**CDK5R1**	0.81	6.15	7.57	NS	0.7	0.76	16.15	21.32	NS	1.6
ENSG00000172216	**CEBPB**	46.16	85.80	1.86	1.50E-03	10.2	58.76	78.50	1.34	1.20E-03	9.1
ENSG00000243649	**CFB**	463.52	0.32	0.00	7.08E-08	9.3	359.88	1.70	0.00	3.80E-07	11.7
ENSG00000000971	**CFH**	354.48	0.00	0.00	1.96E-09	0.0	287.45	0.37	0.00	1.19E-07	6.5
ENSG00000120885	**CLU**	859.16	23.62	0.03	1.77E-08	0.2	749.50	52.36	0.07	2.38E-07	0.7
ENSG00000134871	**COL4A2**	3.34	81.45	24.36	7.50E-03	1.6	1.65	341.85	207.09	7.00E-04	7.2
ENSG00000047457	**CP**	352.74	0.03	0.00	7.60E-08	0.0	196.89	3.79	0.02	2.00E-04	4.5
ENSG00000135678	**CPM**	6.01	2.18	0.36	1.00E-04	4.5	4.96	2.02	0.41	3.00E-04	6.2
ENSG00000203710	**CR1**	0.21	0.00	0.00	3.80E-03	0.0	0.02	0.02	0.71	NS	0.1
ENSG00000117322	**CR2**	0.02	0.06	2.24	NS	0.0	0.01	0.18	14.21	NS	0.0
ENSG00000159176	**CSRP1**	17.24	50.82	2.95	NS	7.1	15.99	35.90	2.25	NS	5.2
ENSG00000164733	**CTSB**	153.65	419.75	2.73	NS	30.7	100.71	281.13	2.79	6.70E-03	23.8
ENSG00000109861	**CTSC**	4.07	13.15	3.23	NS	0.2	0.77	11.32	14.65	NS	0.1
ENSG00000117984	**CTSD**	361.54	209.38	0.58	0.00E+00	1.3	254.41	468.06	1.84	2.40E-03	3.0
ENSG00000103811	**CTSH**	15.38	1.44	0.09	1.67E-06	0.2	32.69	4.18	0.13	0.00E+00	0.7
ENSG00000256043	**CTSO**	18.67	8.72	0.47	0.00E+00	9.7	15.74	6.99	0.44	2.00E-04	8.0
ENSG00000163131	**CTSS**	12.91	0.13	0.01	0.00E+00	8.6	5.49	0.08	0.01	0.00E+00	1.5
ENSG00000163739	**CXCL1**	1.00	3.41	3.41	NS	2.5	0.60	2.14	3.59	NS	1.0
ENSG00000058866	**DGKG**	0.14	0.13	0.90	1.30E-03	0.6	0.10	0.34	3.35	NS	1.2
ENSG00000102780	**DGKH**	0.44	8.08	18.52	1.60E-03	2.9	0.35	4.43	12.57	NS	1.6
ENSG00000135905	**DOCK10**	0.89	0.03	0.03	1.00E-04	0.1	0.52	0.48	0.93	3.60E-03	1.2
ENSG00000128512	**DOCK4**	2.70	14.85	5.49	NS	1.3	2.65	14.46	5.45	NS	1.1
ENSG00000088387	**DOCK9**	2.34	60.04	25.65	9.00E-04	4.8	1.98	75.87	38.40	6.10E-03	9.7

ENSG00000197635	**DPP4**	14.18	9.55	0.67	1.00E-04	2.5	24.22	137.71	5.69	NS	42.3
ENSG00000138166	**DUSP5**	3.86	264.36	68.56	0.00E+00	7.5	2.19	139.50	63.83	4.00E-04	4.0
ENSG00000139318	**DUSP6**	17.32	48.77	2.82	NS	0.7	11.26	36.12	3.21	NS	0.3
ENSG00000127334	**DYRK2**	0.94	20.07	21.38	NS	1.2	0.89	13.78	15.45	NS	0.7
ENSG00000110047	**EHD1**	5.86	60.32	10.29	1.00E-03	3.8	5.90	64.74	10.97	3.20E-03	4.3
ENSG00000164308	**ERAP2**	5.50	10.93	1.99	NS	84.1	0.79	0.30	0.38	1.30E-03	6.2
ENSG00000126218	**F10**	77.41	0.29	0.00	3.90E-08	0.4	70.46	11.16	0.16	0.00E+00	4.3
ENSG00000180210	**F2**	987.30	1.17	0.00	7.84E-08	11.0	834.58	20.52	0.02	1.21E-07	98.8
ENSG00000117525	**F3**	1.20	24.73	20.65	4.00E-04	2.7	0.43	66.00	154.20	4.00E-04	13.9
ENSG00000198734	**F5**	49.86	0.06	0.00	1.25E-06	1.0	51.44	1.43	0.03	0.00E+00	11.4
ENSG00000057593	**F7**	89.93	0.10	0.00	6.11E-07	0.3	116.37	2.56	0.02	5.20E-06	6.4
ENSG00000185010	**F8**	2.69	1.47	0.55	3.30E-03	1.4	1.32	0.89	0.68	1.30E-03	0.6
ENSG00000158869	**FCER1G**	14.87	0.99	0.07	1.00E-04	1.7	6.19	1.84	0.30	4.00E-04	2.3
ENSG00000085265	**FCN1**	0.92	0.00	0.00	6.54E-07	#DIV/0!	0.51	0.01	0.02	2.65E-06	#DIV/0!
ENSG00000137714	**FDX1**	22.41	25.03	1.12	0.00E+00	1.8	22.47	34.04	1.51	2.00E-04	2.3
ENSG00000115414	**FN1**	272.71	1865.14	6.84	NS	31.9	256.27	2388.22	9.32	NS	62.2
ENSG00000010810	**FYN**	5.78	48.64	8.41	4.70E-03	2.2	4.22	48.76	11.55	NS	2.0
ENSG00000107485	**GATA3**	0.50	208.29	420.47	7.72E-07	747.8	0.29	184.56	639.65	2.45E-06	607.1
ENSG00000115271	**GCA**	2.59	28.06	10.83	2.50E-03	1.2	2.09	25.07	12.00	NS	0.9
ENSG00000197045	**GMFB**	3.98	61.61	15.50	9.50E-03	1.1	3.19	97.26	30.47	3.80E-03	1.2
ENSG00000114353	**GNAI2**	25.45	41.03	1.61	2.00E-04	0.6	21.28	61.83	2.91	4.40E-03	0.7
ENSG00000065135	**GNAI3**	2.19	23.73	10.85	1.40E-03	1.7	2.09	21.20	10.14	NS	1.4
ENSG00000172354	**GNB2**	37.17	190.71	5.13	NS	2.2	39.85	187.88	4.71	NS	2.2
ENSG00000114450	**GNB4**	0.31	35.25	113.24	NS	1.6	0.15	43.40	291.43	NS	1.8
ENSG00000186469	**GNG2**	1.04	1.16	1.11	9.70E-03	0.1	0.58	17.81	30.60	NS	1.0
ENSG00000167083	**GNGT2**	0.51	0.00	0.00	0.00E+00	0.0	0.20	0.05	0.26	1.00E-04	0.1
ENSG00000115159	**GPD2**	1.07	38.54	36.08	2.00E-04	1.2	0.70	46.91	66.77	2.00E-04	1.4
ENSG00000177885	**GRB2**	15.97	80.56	5.05	NS	1.2	16.18	90.04	5.57	NS	1.3
ENSG00000145649	**GZMA**	3.44	0.00	0.00	3.23E-06	#DIV/0!	1.68	0.00	0.00	3.33E-06	#DIV/0!
ENSG00000100453	**GZMB**	1.19	0.00	0.00	0.00E+00	#DIV/0!	0.35	0.03	0.08	0.00E+00	#DIV/0!
ENSG00000113088	**GZMK**	1.87	0.00	0.00	1.57E-06	#DIV/0!	0.84	0.03	0.03	6.28E-06	1.2
ENSG00000101076	**HNF4A**	100.42	0.57	0.01	3.22E-08	92.0	93.59	32.79	0.35	4.00E-04	553.1
ENSG00000116983	**HPCAL4**	0.01	0.14	9.19	5.40E-03	0.1	0.00	1.14	270.77	NS	1.4

ENSG00000204389	**HSPA1A**	16.31	72.22	4.43	NS	2.2	39.60	71.08	1.79	NS	2.2
ENSG00000044574	**HSPA5**	62.71	682.50	10.88	NS	1.4	125.79	616.12	4.90	NS	1.1
ENSG00000136244	**IL6**	0.04	0.32	8.95	NS	0.5	0.01	0.74	73.22	NS	2.0
ENSG00000125347	**IRF1**	6.24	27.62	4.42	NS	14.9	4.19	12.01	2.87	NS	5.6
ENSG00000168310	**IRF2**	12.32	12.93	1.05	1.00E-04	1.4	10.69	9.15	0.86	0.00E+00	1.1
ENSG00000185507	**IRF7**	6.51	9.20	1.41	NS	4.3	9.80	12.07	1.23	NS	3.7
ENSG00000169896	**ITGAM**	0.89	0.01	0.01	6.04E-06	0.0	0.47	0.05	0.10	0.00E+00	0.1
ENSG00000055957	**ITIH1**	727.41	0.01	0.00	1.29E-09	#DIV/0!	764.58	0.27	0.00	3.43E-09	1.3
ENSG00000096968	**JAK2**	1.49	14.39	9.67	NS	5.5	1.02	13.49	13.20	NS	5.9
ENSG00000120049	**KCNIP2**	0.96	1.27	1.31	3.70E-03	0.5	0.79	1.63	2.05	NS	0.6
ENSG00000115041	**KCNIP3**	0.40	0.11	0.27	0.00E+00	0.2	0.25	0.55	2.18	NS	0.9
ENSG00000068796	**KIF2A**	0.96	58.43	61.10	5.00E-04	1.1	0.64	64.90	101.05	5.00E-04	1.1
ENSG00000167748	**KLK1**	0.03	0.08	2.57	NS	0.0	0.01	0.05	5.92	NS	0.0
ENSG00000164344	**KLKB1**	164.07	0.32	0.00	1.33E-09	0.0	170.45	0.27	0.00	5.59E-09	0.0
ENSG00000115919	**KYNU**	3.61	0.21	0.06	6.13E-07	5.7	3.72	0.76	0.20	0.00E+00	23.9
ENSG00000154655	**L3MBTL4**	2.97	2.49	0.84	0.00E+00	2.1	2.79	1.36	0.49	0.00E+00	1.6
ENSG00000005893	**LAMP2**	43.74	113.26	2.59	NS	4.8	36.59	137.60	3.76	NS	6.1
ENSG00000002549	**LAP3**	40.50	56.58	1.40	3.00E-04	1.4	38.80	50.01	1.29	3.00E-04	1.1
ENSG00000182866	**LCK**	2.38	0.09	0.04	0.00E+00	0.0	0.66	0.05	0.08	0.00E+00	0.0
ENSG00000043462	**LCP2**	2.25	0.03	0.01	0.00E+00	0.1	0.80	0.06	0.07	0.00E+00	0.3
ENSG00000131981	**LGALS3**	4.18	85.85	20.56	1.40E-03	19.9	2.04	97.56	47.71	5.90E-03	17.7
ENSG00000100600	**LGMN**	22.16	64.88	2.93	NS	1.7	19.00	74.32	3.91	NS	1.3
ENSG00000107798	**LIPA**	23.02	20.57	0.89	3.00E-03	0.6	15.39	33.20	2.16	8.80E-03	0.6
ENSG00000123384	**LRP1**	44.14	76.99	1.74	9.00E-04	1.7	39.32	111.59	2.84	9.60E-03	2.5
ENSG00000111144	**LTA4H**	7.78	35.05	4.51	NS	0.5	6.12	35.92	5.86	NS	0.5
ENSG00000012223	**LTF**	0.34	0.04	0.13	6.00E-04	0.1	0.26	0.10	0.38	2.60E-03	0.2
ENSG00000254087	**LYN**	4.58	56.45	12.33	NS	0.9	3.02	34.54	11.44	NS	0.6
ENSG00000185022	**MAFF**	0.32	27.25	84.69	1.00E-04	8.6	0.14	16.31	115.54	3.00E-04	6.8
ENSG00000065833	**ME1**	3.30	38.69	11.72	2.50E-03	5.0	2.47	78.78	31.91	3.00E-04	10.6
ENSG00000157227	**MMP14**	5.72	525.09	91.87	3.78E-06	9.9	3.10	498.85	161.03	7.19E-06	6.3
ENSG00000102996	**MMP15**	19.50	56.58	2.90	NS	1.6	20.52	55.21	2.69	NS	1.6
ENSG00000204301	**NOTCH4**	2.88	0.15	0.05	1.05E-06	0.5	1.58	0.22	0.14	2.19E-06	0.6
ENSG00000173391	**OLR1**	0.14	0.24	1.77	NS	#DIV/0!	0.03	5.54	162.38	NS	523.0

ENSG00000186472	**PCLO**	0.04	7.58	202.80	4.00E-04	3.6	0.00	5.46	1196.81	8.00E-04	2.6
ENSG00000169174	**PCSK9**	14.62	0.02	0.00	5.67E-08	0.0	9.48	0.23	0.02	4.88E-07	0.0
ENSG00000100311	**PDGFB**	1.88	59.13	31.41	2.00E-04	10.2	2.61	30.43	11.65	NS	9.1
ENSG00000164951	**PDP1**	0.77	37.92	49.44	1.00E-04	1.4	0.76	29.44	38.57	2.00E-04	1.2
ENSG00000108518	**PFN1**	193.15	1633.35	8.46	5.40E-03	1.4	197.08	1234.39	6.26	NS	1.0
ENSG00000102174	**PHEX**	0.00	0.88	230.66	3.40E-03	3.3	0.00	0.41	124.86	NS	2.4
ENSG00000121879	**PIK3CA**	2.21	20.34	9.20	NS	3.3	2.22	17.71	7.99	NS	2.8
ENSG00000105851	**PIK3CG**	0.23	0.00	0.00	2.00E-04	0.0	0.09	0.00	0.00	1.00E-04	#DIV/0!
ENSG00000141506	**PIK3R5**	1.07	0.04	0.04	5.95E-06	0.0	0.48	0.13	0.28	0.00E+00	0.0
ENSG00000137193	**PIM1**	5.04	82.13	16.31	4.00E-04	1.3	6.22	120.98	19.44	3.00E-04	2.2
ENSG00000116711	**PLA2G4A**	0.40	2.55	6.39	NS	1.1	0.33	12.11	37.11	NS	5.6
ENSG00000146070	**PLA2G7**	0.87	5.06	5.84	NS	1.3	0.57	1.83	3.22	NS	0.3
ENSG00000104368	**PLAT**	1.41	7.52	5.33	NS	2.8	0.31	5.01	16.05	NS	0.8
ENSG00000011422	**PLAUR**	0.31	45.46	148.18	1.00E-04	6.4	0.08	10.44	123.99	NS	1.0
ENSG00000115956	**PLEK**	3.12	0.06	0.02	1.00E-04	6.0	1.06	0.08	0.07	1.00E-04	1.9
ENSG00000122194	**PLG**	531.41	0.15	0.00	6.04E-09	7.3	580.42	0.29	0.00	2.06E-08	1.7
ENSG00000188313	**PLSCR1**	3.74	67.71	18.10	NS	3.9	3.26	50.38	15.44	NS	2.7
ENSG00000104695	**PPP2CB**	15.06	127.15	8.44	NS	2.6	13.24	121.50	9.18	NS	2.4
ENSG00000149923	**PPP4C**	27.38	120.32	4.39	NS	1.4	25.84	112.76	4.36	NS	1.3
ENSG00000137509	**PRCP**	7.50	13.95	1.86	8.70E-03	0.7	5.50	16.69	3.04	NS	0.8
ENSG00000110851	**PRDM4**	3.05	54.33	17.83	3.00E-04	3.1	2.61	51.57	19.78	9.00E-04	3.2
ENSG00000085377	**PREP**	6.19	38.87	6.28	NS	0.7	5.49	37.05	6.75	NS	0.6
ENSG00000163932	**PRKCD**	4.08	39.89	9.77	2.60E-03	1.7	3.13	29.98	9.57	NS	1.3
ENSG00000010438	**PRSS3**	1.82	0.05	0.03	3.00E-04	0.1	0.35	0.29	0.82	1.10E-03	0.7
ENSG00000178226	**PRSS36**	0.77	0.22	0.29	0.00E+00	0.5	1.03	0.20	0.19	0.00E+00	0.5
ENSG00000080815	**PSEN1**	5.02	44.54	8.88	8.70E-03	2.8	4.04	56.55	13.99	7.30E-03	2.6
ENSG00000240065	**PSMB9**	4.12	0.74	0.18	6.12E-06	0.6	2.76	0.22	0.08	0.00E+00	0.4
ENSG00000183155	**RABIF**	3.09	23.29	7.53	NS	2.0	3.68	28.95	7.86	NS	2.9
ENSG00000132155	**RAF1**	14.97	41.14	2.75	3.70E-03	0.8	16.14	50.00	3.10	NS	1.0
ENSG00000172575	**RASGRP1**	0.36	1.62	4.50	NS	1.0	0.06	3.83	62.17	NS	2.0
ENSG00000173653	**RCE1**	3.07	27.72	9.02	5.10E-03	1.2	2.92	29.77	10.18	7.20E-03	1.3
ENSG00000177105	**RHOG**	9.23	52.91	5.73	NS	1.6	7.41	50.54	6.82	NS	1.8
ENSG00000063978	**RNF4**	4.34	35.57	8.20	6.80E-03	0.8	3.97	41.59	10.48	NS	0.9

ENSG00000189171	**S100A13**	13.43	128.35	9.56	2.60E-03	5.3	6.82	107.30	15.72	NS	3.9
ENSG00000163220	**S100A9**	6.56	114.91	17.52	NS	955.6	2.82	77.74	27.57	NS	1385.4
ENSG00000104112	**SCG3**	0.01	11.83	1606.74	7.94E-07	0.2	0.02	15.16	803.11	1.73E-06	0.3
ENSG00000197249	**SERPINA1**	3218.49	2.16	0.00	2.41E-08	28.9	2099.11	11.57	0.01	6.39E-07	23.5
ENSG00000197632	**SERPINB2**	0.01	0.98	110.63	2.78E-06	2.1	0.01	1.56	205.10	NS	#DIV/0!
ENSG00000117601	**SERPINC1**	2139.15	0.79	0.00	1.82E-09	#DIV/0!	2028.29	11.99	0.01	5.63E-07	20.7
ENSG00000106366	**SERPINE1**	6.63	623.82	94.12	2.00E-04	70.5	17.41	294.22	16.90	3.80E-03	30.8
ENSG00000149131	**SERPING1**	1068.32	133.31	0.12	1.16E-06	4.3	759.02	93.00	0.12	0.00E+00	2.4
ENSG00000111252	**SH2B3**	7.34	18.39	2.50	NS	0.6	5.62	16.72	2.98	NS	0.6
ENSG00000077463	**SIRT6**	4.66	21.64	4.64	NS	1.8	4.05	21.69	5.36	NS	1.7
ENSG00000107742	**SPOCK2**	1.29	1.17	0.91	3.00E-03	1.2	0.52	3.36	6.48	NS	4.5
ENSG00000197122	**SRC**	2.40	75.51	31.40	0.00E+00	4.5	1.41	65.12	46.21	1.00E-04	3.1
ENSG00000103496	**STX4**	12.60	19.85	1.58	NS	1.2	12.11	19.95	1.65	NS	1.2
ENSG00000105825	**TFPI2**	0.44	84.87	192.84	NS	9.0	0.35	20.34	57.72	NS	2.2
ENSG00000102265	**TIMP1**	50.91	148.58	2.92	NS	2.8	17.47	146.55	8.39	NS	2.6
ENSG00000035862	**TIMP2**	11.84	80.83	6.83	NS	3.2	4.89	87.12	17.81	NS	3.1
ENSG00000187045	**TMPRSS6**	130.48	0.11	0.00	1.77E-07	3.4	135.29	0.84	0.01	1.80E-07	10.4
ENSG00000118503	**TNFAIP3**	4.21	21.66	5.14	NS	5.2	4.34	16.37	3.77	NS	7.1
ENSG00000101557	**USP14**	7.87	78.54	9.98	3.90E-03	1.1	8.76	85.26	9.73	7.50E-03	1.0
ENSG00000135655	**USP15**	4.69	9.79	2.09	7.00E-04	1.6	5.24	11.03	2.10	1.90E-03	1.6
ENSG00000156256	**USP16**	6.72	23.12	3.44	NS	1.2	5.72	21.92	3.83	NS	1.2
ENSG00000138592	**USP8**	2.01	14.30	7.13	NS	1.3	2.14	13.98	6.53	NS	1.2
ENSG00000175073	**VCPIP1**	1.99	17.88	8.98	NS	1.8	2.09	14.44	6.92	NS	1.6
ENSG00000015285	**WAS**	4.30	0.80	0.19	1.00E-04	2.2	1.46	1.16	0.79	1.00E-04	2.0
ENSG00000108039	**XPNPEP1**	4.50	49.84	11.08	1.00E-03	2.0	3.64	45.85	12.59	5.90E-03	1.5
ENSG00000148516	**ZEB1**	2.57	2.74	1.06	1.00E-03	3.5	1.86	5.86	3.16	NS	5.6
ENSG00000169946	**ZFPM2**	0.35	0.27	0.80	4.60E-03	2.7	0.17	3.11	18.09	NS	26.1

a. Genes with equal or greater expression in Li-HLCs compared to parental liver highlighted in black.
b. Genes induced during the differentiation (Li-HLCs/Li-iPSCs > 1) highlighted in grey.
