# Supplementary material for: Liver biopsy derived induced pluripotent stem cells provide unlimited supply for the generation of hepatocyte-like cells
Source: PLoS One. 2019 Aug 29;14(8):e0221762. doi: 10.1371/journal.pone.0221762 (PMC6715171; doi:10.1371/journal.pone.0221762)
Supplement: S8 Table — (PDF) [file pone.0221762.s015.pdf]

S8 Table. Gene expression comparison for 27 liver-specific transcription factors between Li-HLCs and parental livers.

		Liver	C101			C496		
Ensembl ID	Gene symbol	Expr.[a]	Expr.[a]	Fold change[b]	p-value[c]	Expr.[a]	Fold change[b]	p-value[c]
ENSG00000111206	**FOXM1**	0.22	45.14	204.49	0.00E+00	62.12	281.4	0.00E+00
ENSG00000177606	**JUN**	4.11	138.65	33.76	1.00E-04	97.94	23.8	6.00E-04
ENSG00000164379	**FOXQ1**	0.07	1.21	18.34	3.00E-04	2.92	44.2	0.00E+00
ENSG00000008083	**JARID2**	1.78	29.81	16.72	9.00E-04	34.39	19.3	1.00E-03
ENSG00000178919	**FOXE1**	0.01	0.08	12.06	2.30E-03	0.09	13.8	N.S.
ENSG00000141448	**GATA6**	3.39	17.06	5.04	N.S.	65.46	19.3	N.S.
ENSG00000273611	**ZNHIT3**	1.89	8.74	4.63	N.S.	9.81	5.2	N.S.
ENSG00000173039	**RELA**	12.64	47.48	3.76	N.S.	35.97	2.8	N.S.
ENSG00000130700	**GATA5**	0.22	0.44	1.98	8.00E-03	2.92	13.1	1.40E-03
ENSG00000132570	**PCBD2**	3.15	4.30	1.37	7.00E-04	4.14	1.3	7.00E-04
ENSG00000100219	**XBP1**	225.06	298.86	1.33	1.00E-04	269.89	1.2	1.00E-04
ENSG00000103241	**FOXF1**	0.82	0.20	**0.24**	1.30E-03	1.01	1.2	N.S.
ENSG00000164749	**HNF4G**	4.19	0.52	**0.13**	1.92E-06	2.71	**0.6**	4.00E-04
ENSG00000119547	**ONECUT2**	4.81	0.44	**0.09**	2.43E-06	1.32	**0.3**	1.00E-04
ENSG00000129514	**FOXA1**	13.54	1.20	**0.09**	2.45E-06	16.52	1.2	2.50E-03
ENSG00000164736	**SOX17**	0.42	0.03	**0.08**	4.57E-06	16.72	39.7	N.S.
ENSG00000170608	**FOXA3**	47.92	3.49	**0.07**	1.63E-06	17.85	**0.4**	3.00E-04
ENSG00000117707	**PROX1**	25.43	1.77	**0.07**	4.58E-06	3.76	**0.1**	0.00E+00
ENSG00000169856	**ONECUT1**	1.32	0.07	**0.05**	3.00E-04	0.19	**0.1**	6.00E-04
ENSG00000245848	**CEBPA**	139.75	6.29	**0.05**	2.90E-07	20.74	**0.1**	1.56E-06
ENSG00000136574	**GATA4**	8.21	0.30	**0.04**	5.69E-06	32.07	3.9	N.S.
ENSG00000275410	**HNF1B**	2.22	0.06	**0.03**	9.24E-07	10.59	4.8	N.S.
ENSG00000136630	**HLX**	5.59	0.10	**0.02**	1.44E-08	0.24	**0.0**	7.19E-08
ENSG00000125798	**FOXA2**	13.77	0.12	**0.01**	3.13E-07	28.47	2.1	N.S.
ENSG00000101076	**HNF4A**	97.01	0.57	**0.01**	3.22E-08	32.79	**0.3**	4.00E-04
ENSG00000152804	**HHEX**	46.58	0.18	**0.00**	4.05E-08	0.50	**0.0**	4.38E-06
ENSG00000135100	**HNF1A**	13.94	0.04	**0.00**	2.68E-06	1.34	**0.1**	0.00E+00

a.: Gene expression shown as averaged Transcript Per Million (TPM)
b.: Fold change calculated between patient Li-HLCs and parental liver
c.: p-value calculated by a Limma contrast between patient Li-HLCs and grouped parental livers.
