# Supplementary material for: Liver biopsy derived induced pluripotent stem cells provide unlimited supply for the generation of hepatocyte-like cells
Source: PLoS One. 2019 Aug 29;14(8):e0221762. doi: 10.1371/journal.pone.0221762 (PMC6715171; doi:10.1371/journal.pone.0221762)
Supplement: S9 Table — (PDF) [file pone.0221762.s016.pdf]

S9 Table. Genes identified by gene and sample clustering as cluster 2.

		Liver	Li-HLC		Fi-HLC	
**Ensembl ID**	**Gene symbol**	**Expr.[a]**	**Expr.[a]**	**Fold change[b]**	**Expr.[a]**	**Fold change[b]**
ENSG00000276850	**#N/A**	0.23	27.98	121.70	20.26	88.14
ENSG00000120457	**KCNJ5**	0.19	21.29	114.47	2.30	12.39
ENSG00000171631	**P2RY6**	0.36	95.86	263.06	11.12	30.51
ENSG00000214049	**UCA1**	0.17	89.20	511.77	0.08	0.47
ENSG00000203857	**HSD3B1**	0.33	120.53	360.16	18.65	55.74
ENSG00000135346	**CGA**	0.09	303.54	3494.28	355.73	4095.07
ENSG00000088726	**TMEM40**	0.03	58.84	1785.28	4.04	122.48
ENSG00000137869	**CYP19A1**	0.01	12.45	2007.72	7.77	1252.93
ENSG00000102243	**VGLL1**	0.04	102.81	2555.71	12.54	311.82
ENSG00000163993	**S100P**	0.15	77.18	521.20	31.24	210.95
ENSG00000138271	**GPR87**	0.03	92.51	3042.08	16.92	556.51
ENSG00000113196	**HAND1**	0.01	254.08	18034.88	379.99	26971.94
ENSG00000128422	**KRT17**	0.03	50.83	1949.42	12.85	492.70
ENSG00000140873	**ADAMTS18**	0.01	41.42	7922.03	11.92	2279.03
ENSG00000115221	**ITGB6**	0.04	229.31	5782.37	10.88	274.34
ENSG00000164764	**SBSPON**	0.08	7.09	91.49	22.55	290.87
ENSG00000169435	**RASSF6**	0.19	8.03	43.04	2.56	13.72
ENSG00000168702	**LRP1B**	0.26	1.22	4.77	2.97	11.63
ENSG00000108576	**SLC6A4**	0.03	3.68	143.78	2.29	89.42
ENSG00000144452	**ABCA12**	0.01	5.03	746.16	1.40	207.58
ENSG00000197616	**MYH6**	0.01	13.10	1162.43	5.51	489.31
ENSG00000179148	**ALOXE3**	0.01	3.97	609.07	0.64	98.84
ENSG00000180318	**ALX1**	0.02	10.01	590.62	3.84	226.46
ENSG00000242950	**ERVW-1**	0.01	5.03	716.59	5.31	756.55
ENSG00000169550	**MUC15**	0.01	6.38	873.80	8.05	1103.04
ENSG00000280241	**#N/A**	0.04	28.04	759.26	2.32	62.78
ENSG00000171462	**DLK2**	0.01	16.66	1521.67	4.10	374.39
ENSG00000016082	**ISL1**	0.01	13.56	1596.47	11.94	1405.25
ENSG00000064195	**DLX3**	0.01	13.88	1538.61	6.10	676.62
ENSG00000069812	**HES2**	0.00	6.00	1325.33	0.92	203.76
ENSG00000108932	**SLC16A6**	0.28	11.98	43.15	5.63	20.29
ENSG00000012124	**CD22**	0.34	8.58	25.46	1.52	4.51
ENSG00000272405	**#N/A**	0.03	14.70	442.76	5.55	167.27
ENSG00000143217	**NECTIN4**	0.03	17.71	593.97	7.68	257.74
ENSG00000166546	**BEAN1**	0.06	3.30	58.83	0.70	12.55
ENSG00000120149	**MSX2**	0.03	83.05	2505.73	82.12	2477.81
ENSG00000125872	**LRRN4**	0.03	65.32	2448.08	30.93	1159.28
ENSG00000173376	**NDNF**	0.24	33.95	139.64	15.34	63.09
ENSG00000175318	**GRAMD2**	0.01	39.12	3147.25	5.87	472.39
ENSG00000145681	**HAPLN1**	0.01	106.72	10598.24	85.78	8518.87
ENSG00000158270	**COLEC12**	0.10	66.19	633.16	85.10	814.09
ENSG00000224940	**PRRT4**	0.08	17.90	228.13	3.75	47.84
ENSG00000223573	**TINCR**	0.07	14.23	213.46	6.11	91.72
ENSG00000133519	**ZDHHC8P1**	0.10	10.24	103.92	12.65	128.41
ENSG00000276971	**#N/A**	0.20	46.54	231.65	15.05	74.92
ENSG00000005001	**PRSS22**	0.24	40.58	171.49	15.12	63.92
ENSG00000150556	**LYPD6B**	0.18	38.48	219.72	11.88	67.86
ENSG00000167874	**TMEM88**	1.27	193.34	152.78	465.63	367.95
ENSG00000132854	**KANK4**	2.30	65.97	28.72	19.07	8.30
ENSG00000183018	**SPNS2**	4.02	67.57	16.81	14.14	3.52
ENSG00000107485	**GATA3**	0.56	196.42	349.51	45.20	80.43
ENSG00000165125	**TRPV6**	0.16	25.33	161.06	7.46	47.44
ENSG00000134258	**VTCN1**	0.35	473.10	1360.37	122.62	352.58
ENSG00000137203	**TFAP2A**	0.03	53.68	2008.17	16.93	633.25
ENSG00000181634	**TNFSF15**	0.10	73.81	731.51	4.03	39.94
ENSG00000148053	**NTRK2**	0.25	32.29	129.21	13.50	54.03

a.: Gene expression shown as averaged Transcript Per Million (TPM)
b.: Fold change of Li- and Fi-HLCs vs. Liver
