# Supplementary material for: Liver biopsy derived induced pluripotent stem cells provide unlimited supply for the generation of hepatocyte-like cells
Source: PLoS One. 2019 Aug 29;14(8):e0221762. doi: 10.1371/journal.pone.0221762 (PMC6715171; doi:10.1371/journal.pone.0221762)
Supplement: S10 Table — (PDF) [file pone.0221762.s017.pdf]

S10 Table. GO gene sets significantly enriched in the gene with a divergent expression compared to all other samples analyzed (Figure 8).

Gene Set Name	# Genes[a]	% of Gene set[b]	p-value	FDR q-value
GO ORGAN MORPHOGENESIS	10	1.2%	1.39E-08	6.25E-05
GO TISSUE DEVELOPMENT	10	0.7%	3.13E-06	1.40E-03
GO POSITIVE REGULATION OF GENE EXPRESSION	10	0.6%	1.00E-05	2.49E-03
GO REGULATION OF MULTICELLULAR ORGANISMAL DEVELOPMENT	9	0.5%	5.09E-05	7.87E-03
GO REGULATION OF TRANSCRIPTION FROM RNA POLYMERASE II PROMOTER	9	0.5%	8.37E-05	9.88E-03
GO CELLULAR RESPONSE TO ORGANIC SUBSTANCE	9	0.5%	1.09E-04	1.13E-02
GO TISSUE MORPHOGENESIS	8	1.5%	7.80E-08	1.28E-04
GO EPITHELIUM DEVELOPMENT	8	0.9%	5.65E-06	1.95E-03
GO ANATOMICAL STRUCTURE FORMATION INVOLVED IN MORPHOGENESIS	8	0.8%	6.20E-06	1.99E-03
GO POSITIVE REGULATION OF MULTICELLULAR ORGANISMAL PROCESS	8	0.6%	9.05E-05	1.04E-02
GO TRANSCRIPTION FROM RNA POLYMERASE II PROMOTER	7	1.0%	9.83E-06	2.49E-03
GO CIRCULATORY SYSTEM DEVELOPMENT	7	0.9%	1.70E-05	3.62E-03
GO EMBRYO DEVELOPMENT	7	0.8%	3.79E-05	6.53E-03
GO POSITIVE REGULATION OF TRANSCRIPTION FROM RNA POLYMERASE II PROMOTER	7	0.7%	7.85E-05	9.88E-03
GO CELLULAR RESPONSE TO ENDOGENOUS STIMULUS	7	0.7%	8.04E-05	9.88E-03
GO MORPHOGENESIS OF AN EPITHELIUM	6	1.5%	3.77E-06	1.54E-03
GO SKELETAL SYSTEM DEVELOPMENT	6	1.3%	7.87E-06	2.21E-03
GO EMBRYO DEVELOPMENT ENDING IN BIRTH OR EGG HATCHING	6	1.1%	2.39E-05	4.46E-03
GO NEGATIVE REGULATION OF TRANSCRIPTION FROM RNA POLYMERASE II PROMOTER	6	0.8%	1.18E-04	1.18E-02
GO CARDIAC CHAMBER MORPHOGENESIS	5	4.8%	8.54E-08	1.28E-04

a. Number of significant genes overlapping with the Molecular Signatures Database (MSigDB) GO gene sets
b. Percentage of the GO gene set represented by the genes in overlap
