# Supplementary material for: Liver biopsy derived induced pluripotent stem cells provide unlimited supply for the generation of hepatocyte-like cells
Source: PLoS One. 2019 Aug 29;14(8):e0221762. doi: 10.1371/journal.pone.0221762 (PMC6715171; doi:10.1371/journal.pone.0221762)
Supplement: S1 File — (PDF) [file pone.0221762.s018.pdf]

S1 File. Materials and Methods

Established Induced Pluripotent Stem Cell lines

Fibroblast-derived induced pluripotent stem cells were acquired from ATCC (ref. *ACS-1011* and *ACS-1007, ATCC* Manassas, Virginia, US). Fibroblast-derived induced pluripotent stem cell line 33D6 was described previously [1].

Primary cell complete culturing medium composition

Dulbecco's MEM medium [DMEM], ref. 31885; 10% fetal bovine serum [FBS], ref. 10270106; 1X MEM Non-Essential Amino Acids [NEAA], ref. 11140; 1X GlutaMAX, ref. 35050061; 100 units/mL Penicillin-Streptomycin [Pen-Strep], ref. 15140122; 10 mM HEPES, ref. 15630080; all from Thermo Fisher Scientific, and 10μM Y27632 ROCK inhibitor, ref. ACS-3030, ATCC Manassas, Virginia, US

Primary cell basal medium composition

DMEM; 10% FBS; 1X MEM NEAA; 1X GlutaMAX; Pen-Strep; 10 mM HEPES; 1% DMSO.

Primary Human Hepatocytes

Primary Human Hepatocytes (PHHs) were isolated from surplus liver resection tissue obtained from patients at the Basel University Hospital with approval from the Ethics Committee of North Western Switzerland (authorization number EKNZ 2014-362). Written informed consent was obtained from all patients enrolled in this study. PHHs were isolated as previously described by Krieger *et al.*[2].

Reprogramming of PLCs into induced pluripotent stem cells

For reprogramming PLCs into iPSCs, PLCs were thawed and seeded in a coted 6-well culture dish and expanded for a total of 45 days to obtain enough cells. 150'000 cells were seeded in 3 wells of a coated 6 multi-well plate 24 hours before the infection, and cultured in antibiotic free conditions. Cells were then reprogrammed with the Sendai Reprogramming Kit (Cytotune iPS-2.0, ref. A16517 Thermo Fisher Scientific) following the manufacturer's instructions and at a MOI of 5, 5 and 3 respectively for SeV-KOS, SeV-hc-MYC and SeV-hKLF4 viral vectors. Twenty-four hours post-infection, medium was replaced by basal ES Cell-medium (Supplementary Materials and Methods) supplemented with Pen-Strep (ref. 15140122, from Thermo Fisher Scientific). Cells were cultured for 8 days, replacing medium every 48 hours. At day 8, culturing medium was replaced by Essential 8 Medium (E8, ref. A1517001, Thermo Fisher Scientific). iPSC colonies emerged by day 12 to 18 post-infection. Reprogramming efficiency was evaluated at day 21 (# emerging colonies/# seeded cells*100). Pluripotent-like cell colonies were expanded together with somatic cells for further 10 days after the colonies emerged, and then passaged using ReLeSR (ref. 05872, Stem Cell Technologies) accordingly to the manufacturer's instructions, without a clonal selection. After two additional passages, E8 medium was replaced by mTESR1 medium (ref. 05852, StemCell Technologies). iPSCs were kept in culture for at least 20 weeks, splitting them at a 1 to 6 ratio on a weekly base. Expression of reprogramming xenogenes was assessed by q-PCR using specific primers (Supplementary Table 2). Xenogene expression-free cells were used for further experiments.

Teratoma formation assay

In vivo experiments were approved by the Basel Cantonal Veterinary office (license number 2266). NOD/SCID mice (NOD.Cg-Prkdcscid Il2rgtm1Wjl/SzJ) from Charles River Laboratories (Sulzfeld, Germany) were bred and maintained under pathogen-free conditions. Mice (8–10-week old female, two per each cell line assessed) were injected subcutaneously in the flank with titrated cell numbers (~1e10^6). Matrigel VC (BD Biosciences) diluted 1:1 in DPBS was used as vehicle. Teratoma formation was monitored twice a week by caliper measurements. Tumor volumes (in mm^3) were determined according to the formula (length x $width^2$)/2. Animals were sacrificed 50 days post-injection. Formalin fixed paraffin embedded teratoma were used for histological analysis by the resident pathologist.

iPSC differentiation into hepatocyte-like cells (HLC)

Differentiation of iPSC into HLC was performed as previously described [1] with the following modifications: hepatocyte maturation basal medium (L15, 10% fetal calf serum) was replaced by HepatoZYME-SFM medium (ref. 17705-021, Thermo Fisher Scientific).

RNA extraction and RT-q-PCR

Total RNA was purified from cells and from liver tissues using the TRIzol reagent (ref. 15596018, Thermo Fisher Scientific) according to the manufacturer's instructions. Purified total RNA was then treated with DNase I (ref. AM1906, DNA-free DNA Removal Kit, Thermo Fisher Scientific) to remove contaminating DNA.

500ng of total RNA was reverse transcribed using the High-Capacity cDNA Reverse Transcription Kit (ref. 4368813, Thermo Fisher Scientific) and used for q-PCR

experiments. q-PCR experiments were performed using FastStart Universal SYBR Green Master (ref.19317900, Roche Diagnostic, Mannheim, DE) and target-specific primes (Supplementary Table 2). The specificity of all the primers was verified by sequencing the PCR products.

Immunocytochemistry

For immune-detection of proteins, adherent cells in a multi-well plate were washed twice with DPBS and then fixed with 4% formaldehyde in DPBS for 30 minutes at room temperature. Formalin was removed and cells washed again twice with DPBS. Cells were incubated with blocking buffer (3% bovine serum albumin [BSA], 3% milk and 0.3% Triton X in DPBS) for 30 minutes at room temperature. The primary antibody was diluted 1:100 or 1:50 in blocking buffer and incubated over-night at 4°C. Cells were washed three times 10 min with DPBS on a rocking shaker. Secondary antibody (goat anti-rabbit Alexa-Fluor 546, ref. A-11003 or goat anti-rabbit Alexa-Fluor 488, ref. A-11034; both from Thermo Fisher Scientific) was diluted 1:500 in DPBS and incubated for 1 hour at room temperature. Nuclei were counterstained with DAPI.

Frozen PLCs were thawed and seeded into a coated culture dish, and expanded for ~ 30 days before they were trypsinized and transferred into a coated multi chamber slide (Ibidi µ-Slides 8 well, ref. 80826). Twenty-four hours later, the cells were fixed, blocked and immunostained as described above.

Pictures were acquired on a Nikon Eclipse Ti-E inverted fluorescence microscope or in alternative, fluorescent pictures were acquired by a laser scanning confocal microscope (Zeiss LSM-710).

Image analysis

To evaluate the frequency of positive cells, five random fields (based on DAPI staining), representing at least 300 cells each were acquired and analyzed using Cell Profiler [3]. A custom pipeline has been developed to identify nuclei (DAPI), segment the cell cytoplasm, identify the specific staining signal and discriminate it from the background allowing to determine the frequency of positive cells for each marker.

Antibodies

Mouse anti-HNF4A, ref. ab55223; mouse anti-HNF4A, ref. ab41898; rabbit anti-Albumin, ref. ab207327; rabbit anti-SOX17, ref. ab224637; rabbit anti-Desmin, ref. ab32362; rabbit anti-TBX3, ref. ab99302; rabbit anti-MESP1, ref. ab129387; rabbit anti-FOXA2, ref. ab 108422; rabbit anti-WT1, ref. ab89901; Rabbit anti-CXCR4, ref. ab124824; and mouse anti-HSA, ref. ab190706, Abcam plc, Cambridge, UK.

Rabbit anti-E-Cadherin [24E10], ref. 3195S and rabbit anti-Vimentin [D21H3], ref. 5741S, Cell Signaling Technology, Leiden, NL.

Rabbit anti-GATA4, ref. PA1-102X, Thermo Fisher Scientific, Waltham, MA USA.

Mouse anti-Cytokeratin 19, ref. PA0799, Novocastra, Biosystems AG, CH.

Cy3- mouse anti-Actin [1A4], ref. C6198, Sigma-Aldrich, Buchs, CH. Mouse anti-TE7, ref. NBP2-50082, Novus Biologicals, Littleton, CO, USA.

Emerald Green Fluorescent Protein Sendai virus reporter assay

To verify if hPLCs were amenable of infection by the Sendai virus, the CytoTune Emerald Green Fluorescent Protein (EmGFP) Sendai Fluorescence Reporter assay (ref. A16519, Thermo Fisher Scientific) was used. Primary liver cells were prepared as follows: roughly 150’000 cells were seeded in each well of a 6 multi-well plate, previously coated as described for PLCs culture and expansion, and cultured for 24

hours in basal ES Cell-medium (DMEM; 10% ES Cell-Qualified FBS, ref. 16141061 Thermo Fisher Scientific; 1X MEM NEAA; 1X GlutaMAX; 10 mM HEPES). The CytoTune EmGFP Sendai virus was diluted for an estimated multiplicity of infection (MOI) of 1, 3 and 6 in culturing medium. Cells were washed twice with DPBS and then incubated with infection medium over night. Cells were washed twice with DPBS, cultured in basal ES Cell-medium for 48 hours and monitored for the GFP expression by an inverted fluorescence microscope. Cells were trypsinized and resuspended in MACS buffer (ref. 130-091-221, Miltenyi Biotec). The efficiency of infection, calculated as frequency of GFP positive cells, was evaluated by fluorescence-activated cell sorting analysis.

TRA-1-60 live staining

Live iPSCs were washed twice in sterile DPBS. Monoclonal mouse anti-TRA-1-60 antibody (ref. NB100-730SS, Novus Biologicals) was diluted 1:50 in plain DMEM/F12 medium (ref. 31330, Thermo Fisher Scientific), added to iPSCs and incubated at 37°C in a cell culture incubator for 30 minutes. Cells were washed twice in DPBS and incubated with secondary antibody (1/500 goat anti-mouse Alexa-Fluor 546 in plain medium, ref. A-11003 Thermo Fisher Scientific) for 30 minutes at 37°C in a cell culture incubator. Cells were washed again with DPBS and imaged with a Zeiss Axiovert 1 fluorescence microscope. DPBS was removed and replaced by fresh culture medium.

TRA-1-60 positive cell selection

10^6 to 10^8 iPSCs were selected by the EasySep Human iPS Cell TRA-1-60 Positive Selection Kit (ref. 18166, StemCell Technologies) according to the manufacturer's instructions. After selection, cells were reseeded on a coated vessel and expanded before further experiments.

iPSC culture maintenance

All the iPSC lines were cultured in maintenance culturing medium (mTESR1, ref. 05852, StemCell Technologies) if not differently specified, and on a thin layer of reduced growth factor basement membrane matrix (Geltrex LDEV-Free, ref. A1413301 Thermo Fisher Scientific). iPSC were passaged using ReLeSR (ref. 05872, Stem Cell Technologies) according to the manufacturer's instructions and using an incubation time of 3 minutes at room temperature. Cells were split on a weekly base and in a ratio of 1 to 6. iPSCs were alternatively frozen in Stem Cell Freezing Media (ref. ATCC ACS-3020, ATCC Manassas, Virginia, US).

Fibroblast derived iPSC lines (*33D6*, *ACS-1011* and *ACS-1007*) were provided at a passage number ≥ 50, patient derived iPSC lines (C101 and C496) have been used between passage 25 and passage 40 for all experiments described in this manuscript.

Elisa assay and Urea production assay

To quantify the secretion of Albumin, Alpha-fetoprotein (AFP) and Alpha 1-antitrypsin (A1AT) we performed ELISA assays on cell culture supernatants from iPSC were seeded at 100'000 cells per well in a 24-well plate in mTERS1 medium. iPSCs were then differentiated for 16 days as described in the manuscript before supernatant harvest. PHH were seeded at 100% confluency in a 24-well plate before supernatant harvest. Supernatants were analyzed using the Human Albumin ELISA kit (AssayMax, ref. EA3201-1), Human alpha-Fetoprotein ELISA kit (AssayMax, ref. EF6011-1) and Human alpha-1-Antitrypsin ELISA kit (AssayMax, ref. EA5001-1), following the manufacturer's instructions.

Urea secretion was analyzed with an urea production assay (Sigma Aldrich, ref. MAK006-1KT) following the manufacturer's instructions. All the assays were performed in duplicate on supernatants from independent cell cultures.

Cytochrome P450 1A2 (CYP1A2) and 3A4 (CYP3A4) activity assay

To measure the Cytochrome P450 1A2 (CYP1A2) and 3A4 (CYP3A4) activities, we used the P450-Glo Assays (Promega, respectively ref. V8771 and V9001), following the manufacturer's instruction, including the CellTiter-Glo Luminescent Cell Viability Assay (Promega, ref. G7570) to normalize P450-Glo Assay values to cell number.

Oil-red-O and Periodic Acid Schiff staining

To visualize lipid droplets and glycoproteins or polysaccharides accumulation in HLCs, we respectively used Oil Red O (ORO) and Periodic Acid-Schiff stains (PAS).

ORO stock solution was prepared as follow: ORO powder (ref. O0625, Sigma-Aldrich) was diluted at 0.5% (W/V) in isopropyl alcohol and filtered through a paper filter. ORO working solution was obtained by adding 20 mL of distilled water to 30 mL of ORO stock solution. Cells were fixed for 30 minutes at room temperature with 4% formaldehyde, washed twice with DPBS and then with 60% isopropyl alcohol. Cells were then incubated 15 minutes with ORO working solution and then washed again with 60% isopropyl alcohol, monitoring the stain development with a microscope. Nuclei were then counterstained with Hematoxylin. Images were acquired at an inverted Zeiss Axiovert 1 microscope.

For PAS staining we used the Periodic Acid-Schiff Kit (ref. 395B, Sigma-Aldrich) following the instructions from the manufacturer.

RNA-seq and gene expression profiling

RNA libraries were prepared using the Illumina TruSeq Stranded mRNA Sample Preparation protocol (ref. RS-122-2101, Illumina, San Diego CA, US) and sequenced using the Illumina HiSeq2500 platform. Samples were sequenced in paired-end mode

to a length of 2x76 base pairs. Images from the instrument were processed to generate FASTQ sequence files. Read quality was assessed by running FastQC (version 0.10). Sequencing reads showed excellent quality, with a mean Phred score greater than 30 for all base positions. A total number of 1'381 billion 76-bp read pairs (e.g. from a minimum of 40 million to a maximum of 65 million read pairs per sample) were mapped to the Homo sapiens genome (GRCh38) and the human gene transcripts from Ensembl v76 [4] by using an in-house gene quantification pipeline [5]. On average, more than 95% of the total reads were mapped to the exons and junctions (expressed reads). Genome and transcript alignments were used to calculate gene counts based on Ensembl gene IDs. The RNA-sequencing data have been deposited at the European Genome-Phenome Archive (EGA, https://ega-archive.org), under accession number EGAS00001002676. The data is accessible to the research community via the controlled access procedure of the EGA [6].

Raw-counts data were used for the following analysis, using the web utility MeV [7]. Briefly, data were normalized according to the DESeq method [8] and then used for differential gene expression analysis (Limma contrast analysis [9]), for principal component analysis (PCA) and for gene and sample clustering analysis.

Pathways analysis was performed using the web tool from the Broad Institute for computing the overlaps between custom gene sets (differentially expressed genes) and gene sets in the Molecular Signatures Database (MSigDB 3.0) [10]. Liver-specific pathways were selected from the Hallmark gene sets (H), based on the canonical liver metabolisms. Alternatively C2 (curated gene sets) and C5 (Gene Ontology) were also used. Alternatively, raw-counts data were converted into Transcript-Per-Million (TPM) [11] and used for gene expression profiling and correlation analysis. Venn diagrams were generated by the web utility Venny [12].

References

[1] Sullivan GJ, Hay DC, Park IH, Fletcher J, Hannoun Z, Payne CM, et al. Generation of functional human hepatic endoderm from human induced pluripotent stem cells. Hepatology (Baltimore, Md) 2010;51:329-335.

[2] Krieger SE, Zeisel MB, Davis C, Thumann C, Harris HJ, Schnober EK, et al. Inhibition of hepatitis C virus infection by anti-claudin-1 antibodies is mediated by neutralization of E2-CD81-claudin-1 associations. Hepatology (Baltimore, Md) 2010;51:1144-1157.

[3] Carpenter AE, Jones TR, Lamprecht MR, Clarke C, Kang IH, Friman O, et al. CellProfiler: image analysis software for identifying and quantifying cell phenotypes. Genome biology 2006;7:R100.

[4] Cunningham F, Amode MR, Barrell D, Beal K, Billis K, Brent S, et al. Ensembl 2015. Nucleic acids research 2015;43:D662-669.

[5] Schuierer S, Roma G. The exon quantification pipeline (EQP): a comprehensive approach to the quantification of gene, exon and junction expression from RNA-seq data. Nucleic acids research 2016;44:e132.

[6] Lappalainen I, Almeida-King J, Kumanduri V, Senf A, Spalding JD, Ur-Rehman S, et al. The European Genome-phenome Archive of human data consented for biomedical research. Nature genetics 2015;47:692-695.

[7] Howe EA, Sinha R, Schlauch D, Quackenbush J. RNA-Seq analysis in MeV. Bioinformatics (Oxford, England) 2011;27:3209-3210.

[8] Kadota K, Nishiyama T, Shimizu K. A normalization strategy for comparing tag count data. Algorithms for molecular biology : AMB 2012;7:5.

[9] Ritchie ME, Phipson B, Wu D, Hu Y, Law CW, Shi W, et al. limma powers differential expression analyses for RNA-sequencing and microarray studies. Nucleic acids research 2015;43:e47.

[10] Liberzon A, Birger C, Thorvaldsdottir H, Ghandi M, Mesirov JP, Tamayo P. The Molecular Signatures Database (MSigDB) hallmark gene set collection. Cell systems 2015;1:417-425.

[11] Wagner GP, Kin K, Lynch VJ. Measurement of mRNA abundance using RNA-seq data: RPKM measure is inconsistent among samples. Theory in biosciences = Theorie in den Biowissenschaften 2012;131:281-285.

[12] Oliveros JC. Venny. An interactive tool for comparing lists with Venn's diagrams. 2007-2015 [cited; Available from: http://bioinfogp.cnb.csic.es/tools/venny/index.html
